# Effect of cellular nutrient economy on the evolution of genome size in phytoplankton

**DOI:** 10.1126/sciadv.aee2207

**Published:** 2026-07-08

**Authors:** Carlos Caceres, Marc Krasovec, Olivier Crispi, Sebastien Gourbiere, Gwenael Piganeau

**Affiliations:** ^1^ Sorbonne University, University of Perpignan via Domitia, CNRS, LBBM, Banyuls-sur-Mer, France.; ^2^ CNRS, Laboratoire Génome et Développement des Plantes, University of Perpignan via Domitia, LGDP, Perpignan, France.; ^3^ CNRS, Sorbonne University, LOMIC, Banyuls-sur-Mer, France.

## Abstract

The origin of genome size variation remains a central question in evolutionary biology. While energetic costs have been proposed to influence genome size through selection on insertions and deletions (indels), nutrient availability may be a more relevant constraint in primary producers such as phytoplankton. We derived an expression for the selection coefficient of indels based on the phosphorus and nitrogen costs of nucleotides and the cellular nutrient requirements. Selection coefficient estimates indicate that natural selection dominates over genetic drift and favors the fixation of mutations that reduce genome size in phytoplankton with low nutrient requirements. Model predictions are supported by comparative genomics and metagenomic analyses. Together, this model provides a rigorous quantitative framework for understanding genome size evolution, particularly in small cells and oligotrophic environments, highlighting how nutrient limitation drives genome streamlining.

## Teaser

A nutrient cellular economy model explains how phosphorus and nitrogen limitation shapes the evolution of genome size.

## INTRODUCTION

Genome size spans more than five orders of magnitude, from 1.3 Mb in free-living prokaryotes to more than 100 Gb in certain eukaryotes (*1*, *2*). Such a variation is primarily driven by differences in the size of noncoding regions (*3*–*5*). Present genome sizes result from past genome expansion and compaction dynamics driven by molecular processes generating insertion and deletion (“indel”) mutations and evolutionary processes shaping their fixation (*3*). Here, we use indel in a broad sense to refer to any mutation generating a genome size variation, which includes deletions, insertions, duplications, hybridizations, or horizontal gene transfers.

Many post hoc explanations for genome size diversity have been suggested (*5*, *6*). Previous work showed that the relative energetic cost of gene-size insertions was high enough to be selected against, especially in small free-living bacteria (*7*, *8*). Alternatively, the “streamlining hypothesis” stresses that small genome sizes may have evolved to minimize the material cost of replication, as nucleotides contain carbon, nitrogen (N), and phosphorus (P), leading to the selection of species with smaller genomes in resource-limited terrestrial and aquatic environments (*1*, *9*–*14*). However, this hypothesis now lacks a quantitative theoretical framework that links cellular nutrient requirements to the fixation probability of indels, which depends on both natural selection and genetic drift (*3*, *4*). Such a framework requires estimating the selection coefficients*, s*, of indel mutations. Here, we provide selection coefficient estimations based on a model that explicitly links the molecular changes in the number of nitrogen and phosphorus atoms in an indel to the resulting difference in growth rate between the mutant and the wild type. In this way, the selection coefficients of indels are quantitatively estimated as a function of the changes in cellular nutrient requirements they cause. To validate our theoretical framework, we also perform comparative genomic and metagenomic analyses, which provide empirical support for our *s* estimates.

Phytoplankton constitute a diverse group of photosynthetic organisms that provide key ecosystem services (*15*) and offer a particularly suitable framework to investigate the influence of nutrients on genome size evolution. Phytoplankton include some of the smallest genomes among free-living prokaryotes and eukaryotes: <2-Mb genomes in *Prochlorococcus* (*16*, *17*) and 13-Mb genomes in *Ostreococcus* (*18*–*20*). Moreover, phytoplankton inhabit ecosystems that show a wide range of nutrient availability. Low N and P levels limit phytoplankton growth in vast areas of the ocean, driving the evolution of traits that enhance nutrient uptake and utilization (*21*–*24*). Furthermore, and crucially, the growth rate of phytoplankton can be described as a function of the intracellular nutrient content, or quota, of the most limiting nutrient by using the quota model (*25*). The model’s key parameter, *Q*_min_, is the minimum quota and has been estimated for several phytoplankton species (*26*). Phytoplankton thus provide a unique opportunity to estimate selection coefficients of indel mutations based on the changes that they cause in cellular nitrogen and phosphorus content.

## RESULTS

### Noncoding regions drive between-species genome size variations in phytoplankton

**Fig. 1.**
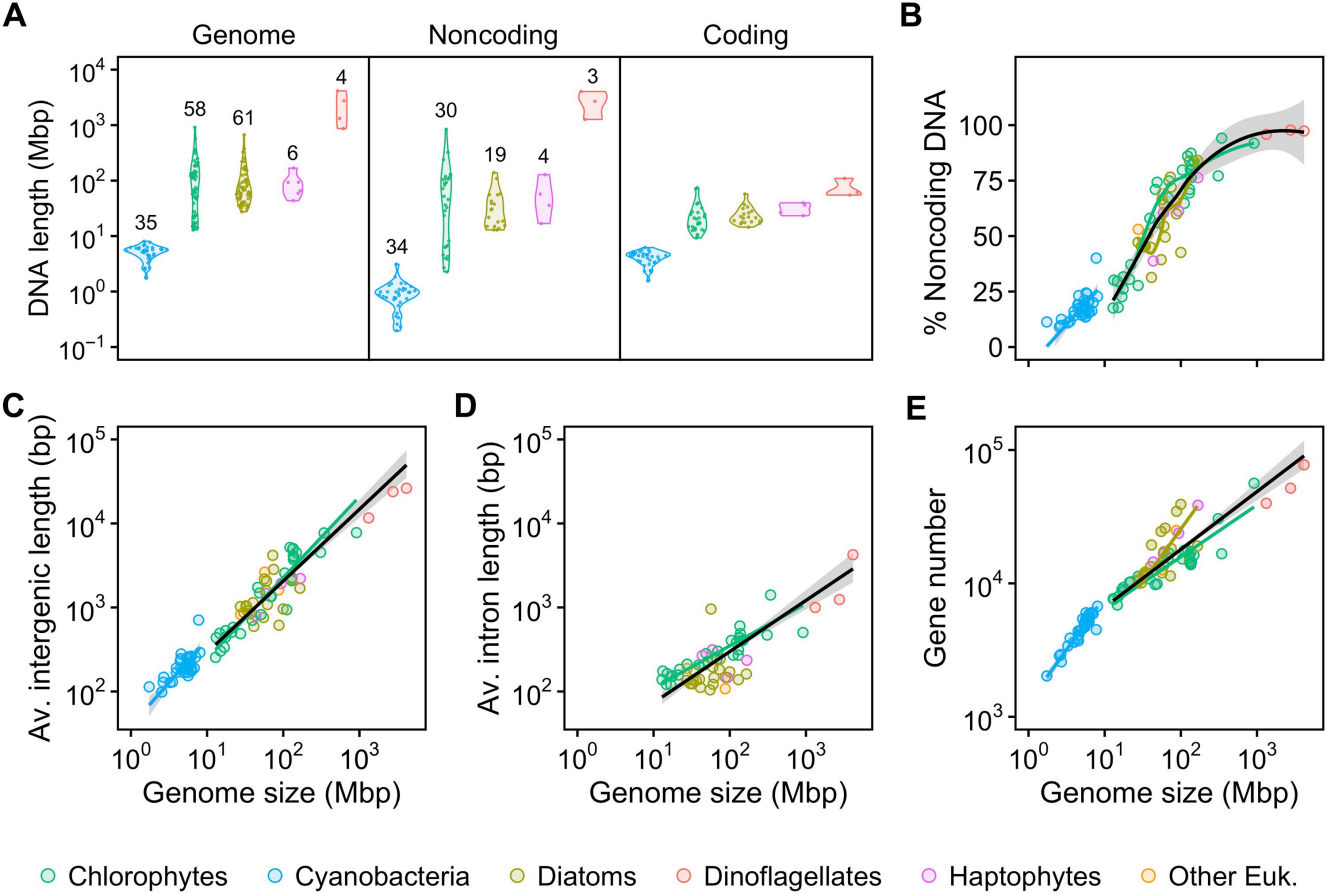
Genome size variation in relation to noncoding and coding regions. (**A**) Violin plots of the size distribution of total genome, noncoding, and coding regions across phyla. Numbers indicate the number of observations per group; values for coding and noncoding regions are identical. (**B**) Relationship between genome size and the percentage of noncoding DNA. (**C**) Relationship between genome size and average intergenic region length. (**D**) Relationship between genome size and average intron length. (**E**) Relationship between genome size and gene number. Each point represents a different species. Black lines indicate the best-fit regression across all eukaryotic species, with 95% confidence intervals shown in gray. Best-fit lines for individual taxonomic groups are shown when the slope is significantly different from zero and the number of observations exceeds four. Local regression was used for (B) instead of standardized major axis (SMA) regression. Statistics for the SMA regressions are provided in table S3.

To investigate genome size variations in phytoplankton, we analyzed 229 genomes from 35 bacteria and 142 eukaryotic species (data S1), spanning six of the seven major photosynthetic lineages (*27*). Cyanobacterial genomes range from 1.6 to 8 Mb, whereas eukaryotic genomes span from 13 Mb in *Ostreococcus tauri* (chlorophyte) to 4.1 Gb in *Prorocentrum cordatum* (dinoflagellate) (Fig. 1A and fig. S1A). In eukaryotes, genome size variation is primarily driven by differences in noncoding DNA content, ranging from 2.2 Mb (17.6%) in *O. tauri* to more than 4 Gb (97.4%) in *P. cordatum* (Fig. 1, A and B, and fig. S1A). Both intergenic and intronic regions contribute substantially to genome size, averaging 41 and 17% of eukaryotic genomes, respectively; in 50 of 59 (85%) annotated species, intergenic DNA exceeds intronic DNA (fig. S1B). Genome size correlates positively with the average size of both intergenic and intronic regions (Fig. 1, C and D). Lineage-specific patterns are also evident. In diatoms, coding and noncoding DNAs contribute similarly to genome length (45 and 55% on average, respectively), whereas chlorophytes with similar genome sizes have a higher fraction of noncoding DNA (72 and 28%; Fig. 1, A and B, and fig. S1A). However, diatom genomes have a higher proportion of intergenic regions than chlorophytes with a similar genome size [analysis of covariance (ANCOVA), *F*_1,33_ = 19.97, *P* < 0.001; fig. S1B], reflecting a higher gene number and fewer introns per gene (ANCOVA, genes: *F*_1,33_ = 6.58, *P* < 0.05, Fig. 1E; introns per gene: *F*_1,33_ = 100.58, *P* < 0.001). Together, genome sizes in unicellular phytoplankton span four orders of magnitude, a variation that largely reflects differences in noncoding DNA content. This pattern is consistent with previous datasets spanning both unicellular and multicellular organisms (*4*, *5*, *28*–*30*).

### Estimates of selection coefficients of indels

Organismal growth is often constrained by the availability of nutrients. In phytoplankton, the quota model (*25*) is widely used to describe growth rate as a function of the intracellular content (i.e., the quota, *Q*) of the limiting nutrient. The growth rate increases as *Q* rises above the minimum quota (*Q*_min_), which is the quota at which the growth rate equals zero, also referred to as subsistence quota. Building on this framework, we derived the selection coefficients of indel mutations occurring in noncoding regions from their associated nutrient cost (Δ*Q*_min_) in 114 species with available measurements of *Q*_min_ (data S2, fig. S2, and table S1) (*26*, *31*–*34*)

$$s=\left(\mu_{m}-\mu_{\text{wt}}\right)\;\frac{\text{ln}2}{\mu_{m}}=-\frac{∆Q_{\text{min}}}{Q_{\text{wt}}}\;\text{ln}2$$ (1)

where $\mu_{\text{wt}}$ is the growth rate of wild type, $\mu_{m}$ is the growth rate of mutant, $Q_{\text{wt}}$ is the quota of wild type, and $∆Q_{\text{min}}$ is the nutrient cost of an indel mutation.

**Fig. 2.**
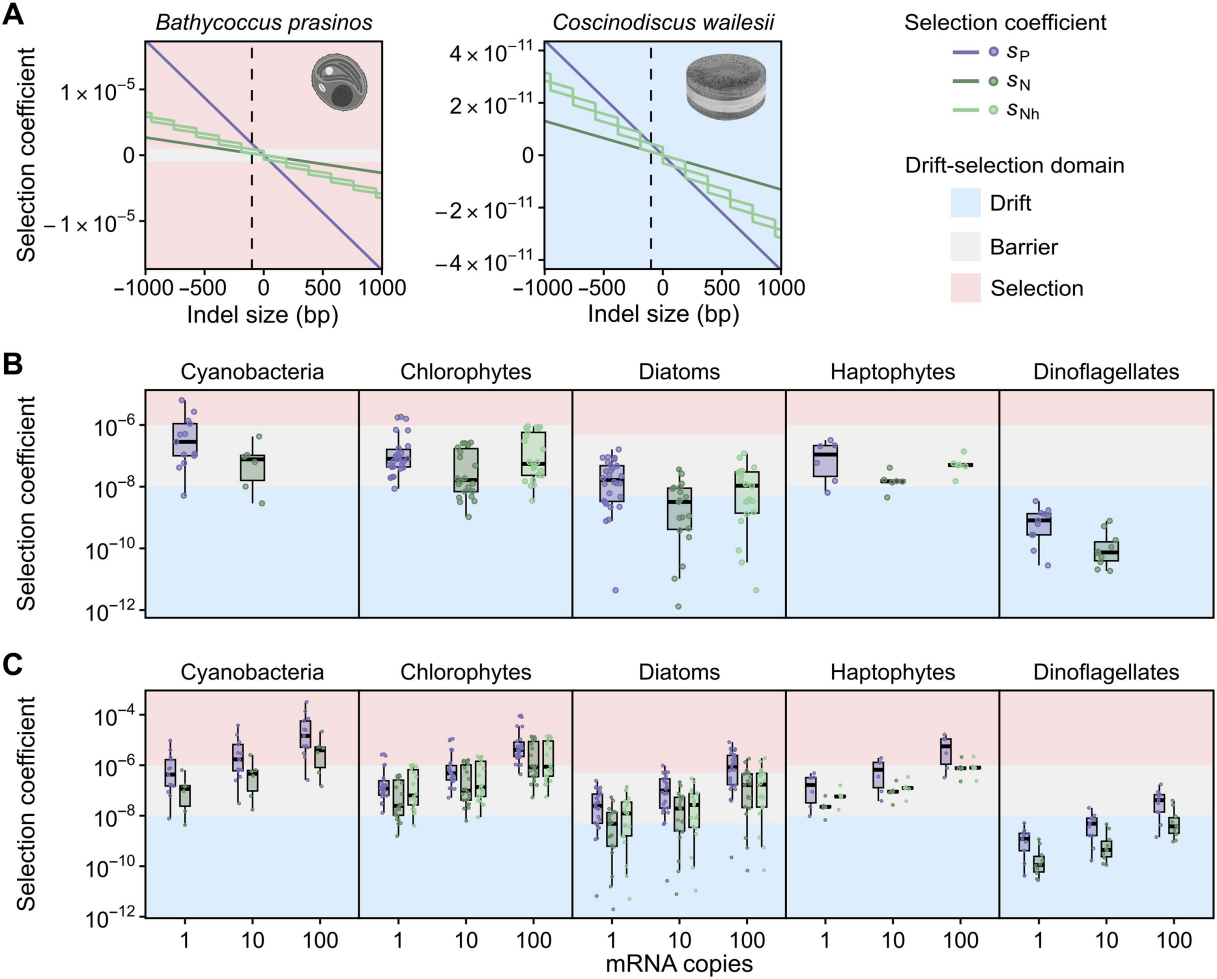
Selection coefficient estimates from available minimum quotas in phytoplankton. (**A**) Selection coefficients as a function of the size of indels in nontranscribed regions for the chlorophyte *B. prasinos* (low minimum quotas for both P and N: *Q*_minP_ = 0.09 fmol of P per cell and *Q*_minN_ = 2.14 fmol of N per cell) and the diatom *C. wailesii* (large *Q*_min_: *Q*_minP_ = 3.50 × 10^4^ fmol of P per cell and *Q*_minN_ = 4.40 × 10^5^ fmol of N per cell). Selection coefficients were calculated using Eq. 1 with *Q* = 1.5 × *Q*_min_ and reflect the impact of indels on P (*s*_P_) or N (*s*_Nh_ and *s*_N_, with or without considering the N content of histones) requirements under P- or N-limiting conditions, respectively. Panel background colors indicate the likelihood that the selection coefficient is too low (blue: drift domain) or high enough (pink: selection domain) for natural selection to effectively drive the fixation of a given indel. The barrier domain (gray) depends on the exact value of *N*_e_, which is between 10^6^ and 10^8^ in phytoplankton. The vertical dashed line marks a 100–base pair (bp) deletion, with the corresponding selection coefficients represented in (B). Similar plots for 114 species, including transcribed regions, are available in fig. S3. (**B**) Selection coefficients for 100-bp deletions in nontranscribed regions. Each point represents one species. A version including species identities is provided in fig. S4. (**C**) Selection coefficients for 100-bp deletions in transcribed regions at different expression levels (i.e., number of mRNA copies). Species identities are shown in fig. S5. N. Romero provided the illustrations of phytoplankton species in (A).

We focused on noncoding indels because both our results and previous studies show that they are the main drivers of genome size variation in eukaryotes. The selection coefficients were derived for the effect of indels on phosphorus (*s*_P_) and nitrogen (*s*_N_) cellular requirements under P or N limitation, respectively, assuming no effect of indels on cell functions (see Materials and Methods). According to Eq. 1, these coefficients reflect the relative nutrient cost of an indel with respect to the total cellular budget, Δ*Q*_min_/*Q* (i.e., its fractional cost)*.* Lynch and Marinov (*7*) used a similar expression based on the fractional cellular energy budget to estimate the energetic cost of gene duplication. As expected, larger indels lead to higher absolute values of the selection coefficient, with deletions resulting in positive *s* values and insertions in negative ones (Fig. 2A and fig. S3). Higher absolute *s* values are also promoted by elevated levels of expression (i.e., number of copies of mRNA, *c*_mRNA_), which amplify the effect of indels (see Eqs. 5 and 6 in Materials and Methods; fig. S3).

For eukaryotes, DNA-packaging histones incur a nonnegligible N cost. When this cost is incorporated into selection coefficient estimates (*s*_Nh_), the relationship with indel size remains monotonic but shows sharp changes whenever a nucleosome is added or removed. The same indel size may lead to two different *s*_Nh_ values, depending on whether a nucleosome is gained or lost (Fig. 2A). Thus, *s*_Nh_ depends not only on indel size but also on chromosomal context, as nucleosome density vary across loci (*35*).

The estimated selection coefficients allow us to compare the fixation probability of nontranscribed indels (i.e., *c*_mRNA_ = 0) of equal size across species (Fig. 2, A and B, and figs. S3 and S4). In the case of 100–base pair (bp) deletions, selection coefficients *s*_P_, *s*_N_, and *s*_Nh_ range from 10^−12^ to 10^−6^, spanning six orders of magnitude (Fig. 2B and fig. S4). Equation 1 shows that this between-species variation in *s* values is driven by *Q*_min_ when there is nutrient limitation (*Q* close to *Q*_min_). Thus, using the empirical relationships between *Q*_min_ and cell volume (*26*), *s* can be approximated from cell volume. One of the best-known predictions of population genetics theory is that the minimum |*s*| value required for selection to be effective depends on the inverse of the effective population size, *N*_e_, such that |*s*| > 1/*N*_e_ (|*s*| > 1/2*N*_e_ in diploids) (*36*). In phytoplankton, *N*_e_ ranges from 10^6^ to 10^8^ and is typically around 10^7^ (table S2) (*8*). Accordingly, we consider selection effective when |*s*| > 10^−7^ for haploids and |*s*| > 5 × 10^−8^ for diploids. In the case of 100-bp indels, these thresholds are met in 31 of 97 species analyzed for *s*_P_, 9 of 68 for *s*_N_, and 12 of 52 for *s*_Nh_ (Fig. 2B and fig. S4). In taxa with low *Q*_min_, including *Prochlorococcus*, *Synechococcus*, *Chloropicon*, Mamiellales, or *Phaeodactylum tricornutum*, 100-bp indel mutations have selection coefficients such that |*s*| > 10^−7^ (or |*s*| > 5 × 10^−8^ in *P. tricornutum*) (fig. S4). Selection coefficients may even surpass the conservative threshold of 10^−6^ for longer indels or for those occurring within introns of highly expressed genes (Fig. 2C and fig. S5). In contrast, in taxa with intermediate quotas (e.g., *Chlamydomonas*, *Asterionella formosa*, and *Gephyrocapsa huxleyi*), indels between 100 and 1000 bp are required to reach selection coefficients on the order of |*s*| ≈ 10^−7^ (fig. S3). These values exceed 1000 bp in taxa with higher *Q*_min_ (e.g., *Trichodesmium*) and approach 1 Mb in the largest *Q*_min_ taxa, such as *Coscinodiscus wailesii* and large dinoflagellates. Therefore, our selection coefficient estimates show that P and N limitation can significantly influence selection on indel mutations in the subset of noncoding regions that are dispensable, i.e., those with little or no impact on cellular function, particularly in phytoplankton species with low cellular P and N requirements.

### Stronger selection coefficients associated with P as compared to N limitation

Overall, phosphorus requirements exert a stronger selective pressure on indels than nitrogen requirements (Fig. 2B). Across all species, the selection coefficient associated with phosphorus cellular requirements, *s*_P_, exceeded that associated with nitrogen, *s*_N_. Moreover, in 30 of the 39 species, it was also higher than *s*_Nh_ (fig. S4). The relative impact of P versus N limitation on the selection of a given indel can be assessed by comparing the respective Δ*Q*_min_/*Q*_min_ ratios for P and N. Consequently, the relative impact of P versus N limitation will be higher in taxa with elevated N requirements in relative terms, such as cyanobacteria and chlorophytes, whereas it will be lower in groups with low cellular N:P ratios, such as diatoms and haptophytes (*37*). The nine species with *s*_P_ *< s*_Nh_—six diatoms, two raphidophytes, and one chlorophyte, all diploids except the chlorophyte—have *Q*_minN_/*Q*_minP_ < 13. In transcribed regions, *s*_P_ more frequently exceeds *s*_Nh_ (Fig. 2C and figs. S3 and S5) because higher mRNA copy numbers amplify the P (and N) costs associated with nucleotides, whereas histone-associated N remains unaffected. In addition, the *s* values are nonlinearly affected by the actual quota and growing conditions; *s* is maximum when *Q* approaches *Q*_min_, under extreme nutrient limitation and low growth, and decreases as *Q* increases and nutrient limitation is alleviated (Eq. 1 and fig. S6). Therefore, for most species, except those with low *Q*_minN_/*Q*_minP_ ratios, environments where P is the primary limiting nutrient exert stronger selection for genome reduction than those limited by N.

### Estimation of the fate of duplications

Duplications represent a major form of insertion-type mutations (*4*). To investigate the fate of duplications, we compared the estimates of the minimum length of indels required for selection to be effective, *l*_min_, with the distribution of noncoding region lengths. In eukaryotic species with available *N*_e_ values, *l*_min_ in nontranscribed regions ranged from 6 to 628 bp under P limitation and from 33 to 1235 bp under N limitation, with the lowest values observed in *Ostreococcus* (fig. S7). In *Prochlorococcus*, *l*_min_ was even smaller, only 1 bp under P limitation. In most eukaryotic species, the *l*_min_ estimates, calculated assuming *N*_e_ = 10^8^ and *N*_e_ = 10^6^, are similar or lower than the median sizes of intergenic regions (fig. S7). In most species, the 90th and, especially, the 99th percentile of intergenic region lengths exceeded *l*_min_, which means that intergenic regions larger than *l*_min_ are infrequent. For transcribed regions, *l*_min_ was one-third lower than for nontranscribed regions assuming *c*_mRNA_ = 1 and, in general, also matched the median intron length (fig. S7). This alignment between *l*_min_ and the lengths of noncoding regions suggests that, on average, duplications larger than the median size of noncoding regions are subject to effective purifying selection, or conversely, that deletions removing such duplicated segments may be positively selected.

### Positive relationship between the size of genomic regions and *Q*_min_

**Fig. 3.**
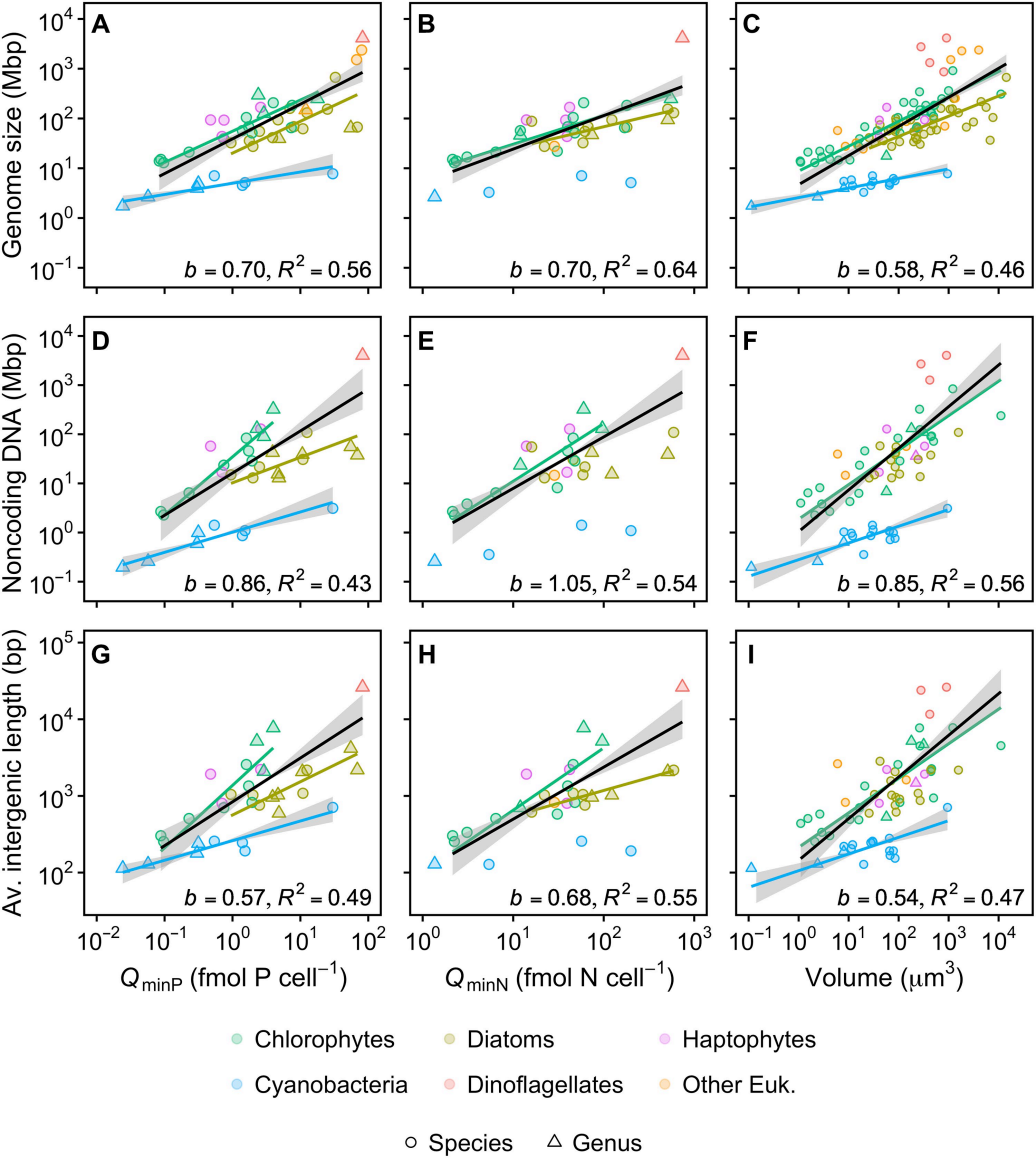
Relationship between genomic traits and *Q*_minP_, *Q*_minN_, and cell volume on a log-log scale. (**A** to **C**) Genome size. (**D** to **F**) Total noncoding DNA. (**G** to **I**) Average size of intergenic regions. Each point represents a different species or genus. Point color and shape indicate taxonomic affiliation and taxonomic level, respectively. Note that the sets of species and the *x*-axis scales are not the same for *Q*_minP_, *Q*_minN_, and cell volume. Black lines represent best-fit SMA regressions when all eukaryotic taxa are pooled together, with slope coefficient, *b*, and coefficient of determination (*R*^2^) reported at the bottom of each panel. Gray shading indicates the 95% confidence interval for regressions of cyanobacteria and eukaryotes. Best-fit regressions for individual taxonomic groups are shown only when slopes were significantly different from zero and the number of observations exceeded four.

A corollary expectation of higher selection coefficients against insertions (or in favor of deletions) in species with low *Q*_min_ is a positive relationship between the current size of genomic regions and *Q*_min_. Genome sizes, as well as the sizes of the different genomic regions (noncoding, coding, intergenic, and intronic), correlate positively with both *Q*_minP_ and *Q*_minN_ measures across eukaryotic species and genera (Fig. 3, fig. S8, and table S4). These correlations remain significant after excluding the large genome outlier observation of the dinoflagellate *Prorocentrum*. The particularly large genomes of dinoflagellates and species such as *Euglena gracilis* might result, in part, from their mixotrophic behavior, which combines phototrophy and phagotrophy and alleviates N and P limitation. Correlations also remain significant when focusing on species-level data (*P* < 0.05, except for the average intron length). Moreover, significant relationships were observed for chlorophytes, cyanobacteria, and, in the case of some DNA regions, diatoms (Fig. 3, fig. S8, and table S4). To ensure that these positive relationships were not solely driven by the fact that *Q*_min_ will increase with the actual P and N content of DNA and thus genome length, we repeated the analyses using *Q*_min_ values corrected by subtracting the N and P content of the focal genome region (fig. S9 and table S5). All relationships remained significant after correction, indicating that DNA nitrogen and phosphorus content alone does not fully explain the positive relationship between genome region lengths and *Q*_min_*.*

Building on the long-standing interest in the relationship between genome size and cell volume (*5*, *38*–*42*), we investigated the association between the size of DNA regions and cell volume. The slope coefficients relating DNA regions size to cell volume were positive and similar to those observed for the relationships between DNA regions with *Q*_min_, particularly *Q*_minP_ (Fig. 3, fig. S8, and table S6). This is as expected because cell volume and *Q*_min_ are positively correlated (*26*). Furthermore, we estimated the variance in genome size and DNA regions size explained by *Q*_min_ and cell volume using partial regression analysis (table S7). Most of the explained variation in genome and DNA region sizes is shared between *Q*_min_ and cell volume, while the proportion explained exclusively by cell volume is minor and often lower than that explained by *Q*_min_ alone (table S7). Thus, our model and these analyses suggest that cellular nutrient requirements, rather than cell volume per se, are the primary drivers of the relationship between genome size and cell volume in phytoplankton. Nevertheless, this does not exclude the possibility that selection for smaller cell volumes, which increase the surface-to-volume ratio and maximize nutrient uptake in oligotrophic environments (*43*), may ultimately affect *Q*_min_ and indirectly promote genome reduction.

### Observed intragenomic variations in intron and intergenic region lengths

**Fig. 4.**
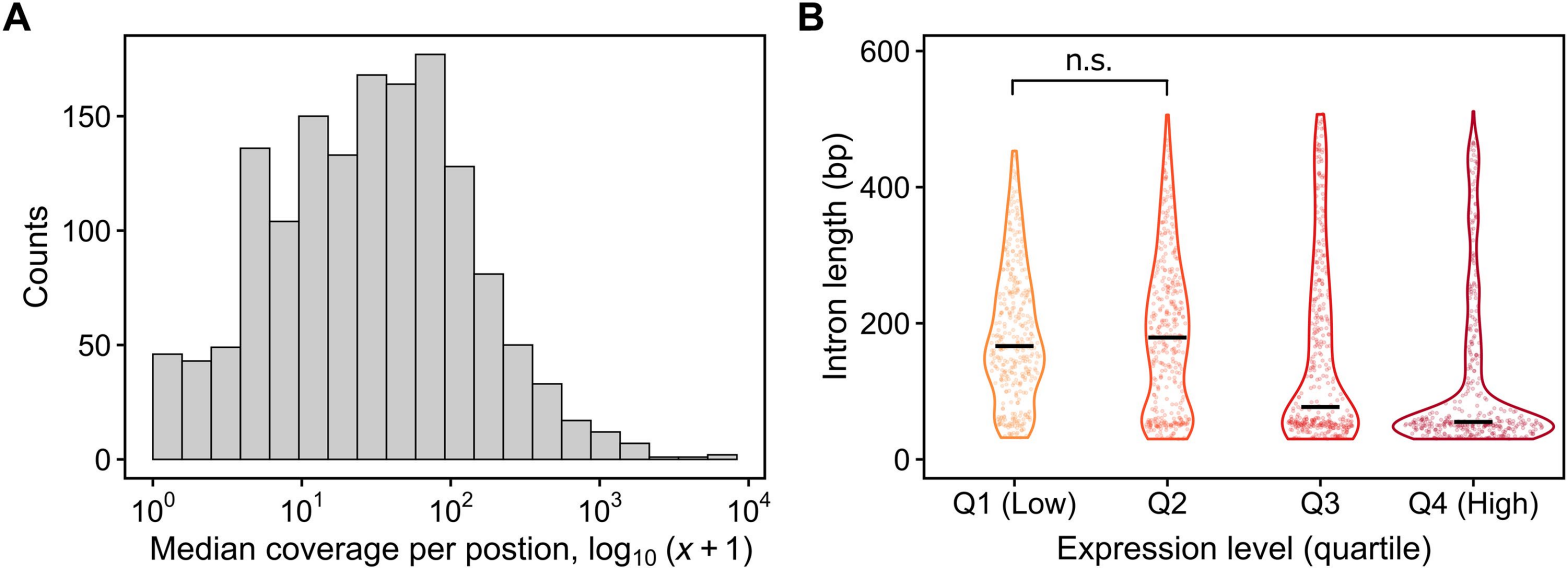
Transcription rates of introns in *B. prasinos*. (**A**) Distribution of median RNA coverage per position across introns. (**B**) Length of introns grouped into quartiles based on their median RNA coverage per position. Black lines show median values. Pairwise Wilcoxon tests showed all comparisons significant except between Q1 and Q2 (table S8). n.s., not significant.

The model further predicts that selection coefficients increase with transcription rate (*s* increases with *c*_mRNA_; Fig. 2C). As a result, shorter deletions are more likely to become fixed, whereas shorter insertions are more likely purged, in highly transcribed regions. This leads to a negative correlation between intron length and transcription rate. Introns in the higher transcription-rate class are significantly shorter than those in regions with lower transcription rates in both *Bathycoccus* and *Ostreococcus* (Fig. 4, fig. S10, and table S8). Transcriptional activity was detected in up to 99% of intergenic sites in these compact genomes, with transcription rates spanning four orders of magnitude (fig. S11). Intergenic region size decreases with increasing expression, although an increase is observed in the highest expression class, consistent with the presence of functional elements in these regions (fig. S11 and table S8). The elemental cost of mRNA thus provides an indirect explanation for the origin of intragenomic variation in intron (*44*) and, to a lesser extent, in intergenic length.

### Indirect evidence of selection against insertions in more oligotrophic environments

**Fig. 5.**
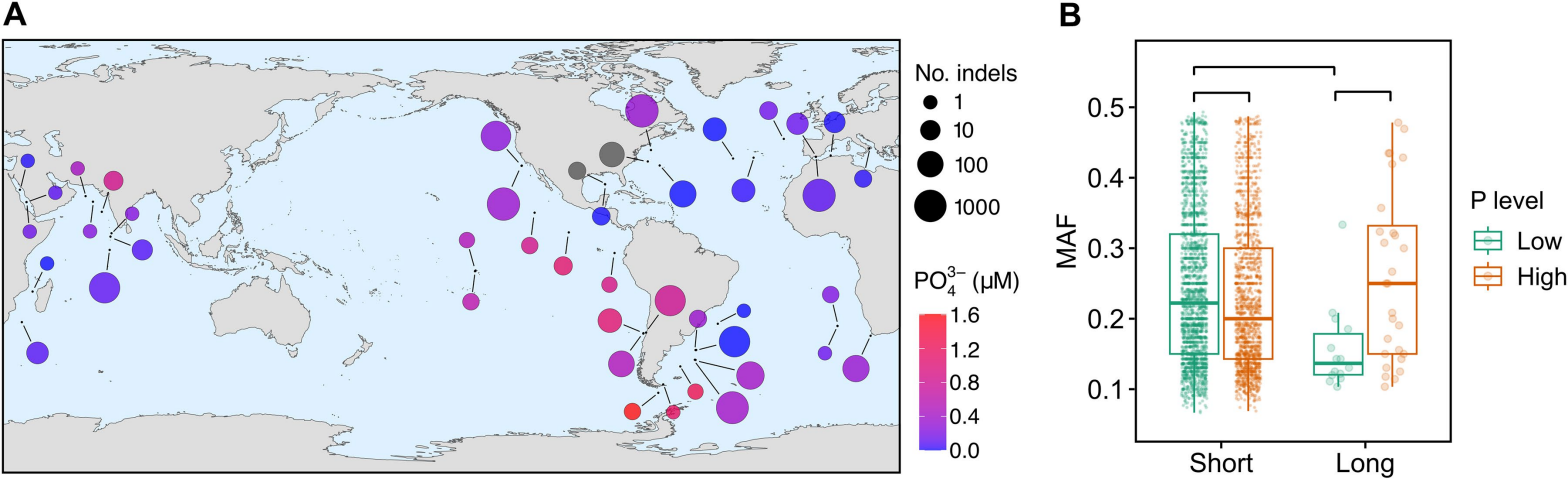
Worldwide distribution of metagenomes with indel polymorphisms and MAF of insertions in low and high phosphate environments. (**A**) Worldwide distribution of metagenomes containing polymorphisms for the six target species belonging to three genera of Mamiellales. Gray circles are shown when phosphate concentrations are not available. (**B**) MAF of short (<10 bp) and long (≥10 bp) insertions in low (green) and high (orange) phosphate concentration environments. Results of the pairwise Wilcoxon tests corresponding to (B) are provided in table S9.

Selection may also leave signatures on mutations still segregating within natural populations (*45*). Under prevalent limiting nutrient conditions, we expect insertions and deletions to be more and less deleterious, respectively, and all the more so in longer indels and oligotrophic waters. This should result in a lower minor allele frequency (MAF) of insertions as compared to deletions, as well as a lower MAF in longer insertions (*46*). We tested these predictions by leveraging metagenomic and associated environmental data from the TARA Oceans pan-oceanic survey (*47*, *48*), focusing on species whose presence has been documented at 68 of the 133 sampling stations (*49*). By mapping reads against the reference genomes, we identified 8260 indels with a coverage of ≥20 reads, located in intergenic regions, and uniquely present in one metagenome (Fig. 5A). Of these indels, 4871 (59%) and 2100 (25%) were affiliated to the cosmopolitan species *Bathycoccus prasinos* and *Ostreococcus lucimarinus* (fig. S13, A and B). Consistent with our hypothesis, the MAF of long insertions (≥10 bp) was significantly lower than the MAF of short insertions in lower P (*W* = 23,069, *P* < 0.001; Fig. 5B) and lower N (*W* = 24,242, *P* < 0.05; fig. S12B) environments, but not in higher nutrient environments (table S9). Moreover, the MAF of long insertions was markedly lower in low-phosphate waters compared with high-phosphate waters (Wilcoxon, *W* = 91.5, *P* < 0.01; Fig. 5B). The MAF comparisons for long insertions between environments with lower and higher N concentrations showed similar trends, although they were not statistically significant (fig. S12 and table S9). However, we did not observe the expected higher MAF for long deletions in low than high nutrient waters, likely because of the negative effect of these long deletions on cell functions in compacted genomes such as those of small Mamiellales (fig. S12). Similar results were observed when analyses were performed separately for *B. prasinos* and *O. lucimarinus* or when using median MAF values per metagenome (figs. S13 and S14 and table S9). Thus, these results are consistent with stronger selection against long insertions in nutrient-poor environments, likely mediated by nutrient economy, and suggest that such selection may act over short evolutionary timescales.

## DISCUSSION

We provide a quantitative theoretical framework for the mechanisms underlying genome streamlining observed in some microorganisms inhabiting oligotrophic waters (*1*, *10*, *16*, *50*). This may also be extended to terrestrial communities, where the biomass of plant species with larger genomes and higher ploidy levels increases in high phosphorus and nitrogen soils (*13*, *14*). The positive relationship between genome size and *Q*_min_ reflects the tendency toward genome contraction and expansion across the *Q*_min_ gradient. In taxa with large (e.g., *C. wailesii*) and intermediate (e.g., *G. huxleyi*) nutrient quotas, the selective pressures mediated by N and P limitation can promote and prevent the fixation of long nonfunctional deletions and insertions near 1 Mb or 1000 bp, respectively. However, shorter insertions are more likely to accumulate through genetic drift and thus restrict genome contraction (*30*). In contrast, in small species with low nutrient quotas such as *Ostreococcus*, P constraints impose selective pressures strong enough to purge insertions as short as 6 bp. In *Prochlorococcus*, these constraints are even more stringent, enabling the purging of insertions as short as 1 bp. Our MAF analyses support selection against intergenic insertions equal or longer than 10 bp in Mamiellales from low-nutrient waters (Fig. 5). Thus, in these small taxa, whose genomes are among the most streamlined of all photosynthetic eukaryotes and prokaryotes, further contraction is primarily constrained by the loss of functions that would compromise cellular performance (*16*).

Energy constraints can also promote genome reduction, as energy is required for nucleic acid synthesis and light limitation alternates with nutrient limitation in oceans and lakes (*7*, *51*). However, selective pressures exerted by N and, especially, P limitation may be stronger. To evaluate this, we compared the fractional cost of indels in terms of nutrients (Δ*Q*_min_/*Q*_min_, i.e., under extreme nutrient limitation) and energy required for DNA replication (Δ*E*/*E*), as reported by Lynch and Marinov (*7*). We found that, in eukaryotic species with a cell volume ≤100 μm^3^, the fractional costs in terms of P and N were one order of magnitude and nearly five times higher, respectively, than energy costs. This corresponds to Δ*Q*_minP_/*Q*_minP_ ≈ 2 Δ*Q*_minN_/*Q*_minN_ ≈ 10 Δ*E*/*E* (fig. S15). The differences in selective pressures exerted by N, P, and energy availability on genome contraction underscore how changes in biogeochemical, ecological, or anthropogenic processes shaping resource limitation [e.g., diazotrophy can alleviate N deficiency or shift ecosystems from N to P limitation; (*52*)] can influence the evolution of genome architecture. This is particularly relevant under the current global change scenario, which might exacerbate N limitation in some regions, such as Arctic waters during summer, and promote P limitation across the global ocean (*53*–*56*).

The availability of carbon, the backbone component of biomolecules, including nucleic acids, may also influence indel selection and genome size. However, phytoplankton rarely experience carbon limitation, and its potential effect on genome size is lower than that of nitrogen and phosphorus, given cellular and nucleotide stoichiometry (*57*). In addition, while not explicitly considered in our theoretical framework, nutrients not incorporated into nucleic acids may indirectly shape indel selection by affecting DNA synthesis via biochemical resource co-limitation (*21*, *58*). For example, iron or manganese scarcity can decrease photosynthetic activity and promote energy limitation, thereby affecting indel selection (*59*–*61*). Similarly, the requirements for iron and molybdenum in the N_2_ fixation machinery may facilitate their influence in diazotrophs (*52*, *62*). Therefore, resources beyond N, P, and energy may also modulate the evolution of genome size in phytoplankton.

Our model suggests several evolutionary implications of N and P constraints on genome evolution. First, higher |*s*| values in species with low *Q*_min_ reflect the higher relative costs of nucleotides in terms of N and P, which also occur for energy (*7*). Second, any variation in *Q*_min_, which can be promoted by a given indel or by any other cellular change, affects the strength of nutrient-mediated selective pressures on future mutations. This generates positive feedback on genome size: A large insertion, such as a whole chromosome duplication, reduces the selection efficiency against subsequent insertions while weakening selection for deletions. Third, changes in *Q*_min_ can result from multiple small indels at different loci, not necessarily a single event, and thus enhance effective selection against mutator alleles biased toward insertions. Fourth, relaxed selection in high *Q*_min_ species increases the likelihood of retaining duplications or horizontally transferred sequences, compared to species with low *Q*_min_ species. Thus, neofunctionalization and genome diversification following gene duplication are more likely in high *Q*_min_ species and nutrient-rich environments but limited in small species or ecotypes thriving in oligotrophic environments. Last, our theoretical framework could be extended to functional noncoding and coding regions, as well as to point mutations associated with nutrient costs. It may also be applicable to viruses or multicellular organisms, provided that cellular nutrient quota estimates are available.

## MATERIALS AND METHODS

### Mathematical expression of selection coefficients associated with an indel mutation

The growth of phytoplankton is often constrained by the availability of nutrients. The quota model (*25*, *63*) describes the intrinsic growth rate (μ; per day) as a function of the intracellular content (i.e., the quota, *Q*; moles per cell) of the limiting nutrient

$$\mu =\mu_{\text{inf}}\left(1-\frac{Q_{\text{min}}}{Q}\right)$$ (2)

where *Q*_min_ (moles per cell) is the minimum quota required for growth (also called the subsistence quota) and μ_inf_ is the maximum growth rate reached at a hypothetical infinite *Q*.

The *Q*_min_ of a mutant (*Q*_min,m_) carrying a spontaneous indel mutation can be estimated from the *Q*_min_ of its corresponding wild type (*Q*_min,wt_) and the change in *Q*_min_ relative to the wild-type (Δ*Q*_min_)

$$Q_{\text{min},m}=Q_{\text{min},\text{wt}}+∆Q_{\text{min}}$$ (3)

where Δ*Q*_min_ > 0 for insertions and Δ*Q*_min_ < 0 for deletions. Δ*Q*_min_ can, in turn, be calculated from the change in the number of atoms (Δ*n*_a_) introduced (or removed) by the indel, either phosphorus (Δ*n*_P_) or nitrogen (Δ*n*_N_)

$$∆Q_{\text{min}}=\frac{∆n_{a}}{A}$$ (4)

where *A* is Avogadro’s number. Δ*n*_a_ depends on the number of base pairs in the indel (Δ*n*_bp_) as well as, in the case of transcribed regions, the number of mRNA copies (*c*_mRNA_). Hence, considering that each nucleotide contains one phosphorus (P) atom, the change in the number of P atoms is given by

$$∆n_{P}=∆n_{\text{bp}}\;\left(2+c_{\text{mRNA}}\right)$$ (5)

Note that *c*_mRNA_ = 0 if the mutation occurs in a nontranscribed intergenic region. Thus, Δ*Q*_minP_ = 2/*A* for a 1-bp insertion occurring in a nontranscribed intergenic region. In the case of Δ*n*_N_, differences in the number of nitrogen (N) atoms in the nitrogenous bases must also be considered

$$∆n_{N}=8{\Delta n}_{\text{GC}}+7{\Delta n}_{\text{AT}}+\left(2{\Delta n}_{U}+3{\Delta n}_{C}+5{\Delta n}_{G}+5{\Delta n}_{A}\right)\;c_{\text{mRNA}}$$ (6)

where Δ*n*_GC_ and Δ*n*_AT_ are the numbers of GC and AT base pairs in the DNA, respectively, and Δ*n*_U_, Δ*n*_C_, Δ*n*_A_, and Δ*n*_G_ are the changes in the number of nucleotides of the corresponding transcribed strand. The nitrogen content per nucleotide is as follows: thymine (T) and uracil (U) contain two, cytosine (C) contains three, and guanine (G) and adenine (A) each contain five N atoms. For simplicity, we assumed a cost of 7.5 nitrogen atoms per base pair change in DNA (assuming ${\Delta n}_{\text{GC}}={\Delta n}_{\text{AT}}$) and 3.75 N atoms per nucleotide in mRNA (assuming equal proportions of the four nitrogenous bases in mRNA). If translated codons were to be considered, then the change in nitrogen atoms associated with the effect of the mutation on amino acids would also need to be included.

Changes in the length of noncoding regions can lead to additional changes in N content by affecting the number of proteins required for DNA packaging. In eukaryotic species, we estimated the changes in nitrogen requirements associated with histones resulting from indel mutations and added this to Δ*Q*_minN_ assuming that chromatosomes, composed of one copy of histone H1 and the nucleosome histone octamer (two copies each of H2A, H2B, H3, and H4), occur every 190 bp (*64*). The N content of histones in each chromatosome was calculated from the amino acid sequences of histones in *P. tricornutum*, obtained from the UniProt database (accessions: B7FX66, B7FZS7, B7FWR8, B7G218, and B7FX68), and was equal to 1772 nitrogen atoms. We accounted for the discrete nature of changes in histone N content: A change in genome size does not necessarily result in a corresponding change in the number of nucleosomes. Therefore, unlike changes in nutrients mediated by nucleotide content, histone-associated N changes can arise from indels affecting a few to hundreds of base pairs. Dinoflagellates were excluded from histone N content calculations due to their low histone abundance and a protein-to-DNA ratio in chromatin that is 10 times lower than that of most eukaryotes (*65*, *66*).

Based on Eqs. 2 and 3, the growth rate of a mutant with an indel mutation that has no effect except for changes in P and N requirements (i.e., μ_inf_ remains unaffected) occurring in a noncoding region constantly expressed across the entire *Q* range (i.e., Δ*Q* = Δ*Q*_min_), is given by

$$\mu_{m}=\mu_{\text{inf}}\left(1-\frac{Q_{\text{min},\text{wt}}+∆Q_{\text{min}}}{Q_{\text{wt}}+∆Q_{\text{min}}}\right)$$ (7)

Thus, the quota model provides a means to link changes in nutrient requirements mediated by indel mutations with changes in the growth rate. The mutant selection coefficient per unit of time (*s*_time_; per day) can be estimated from the difference between the intrinsic growth rate of the mutant (μ_m_) and the wild type (μ_wt_) (*34*)

$$s_{\text{time}}=\mu_{m}-\mu_{\text{wt}}=\mu_{\text{inf}}\left(\frac{Q_{\text{min},\text{wt}}}{Q_{\text{wt}}}-\frac{Q_{\text{min},\text{wt}}+∆Q_{\text{min}}}{Q_{\text{wt}}+∆Q_{\text{min}}}\right)$$ (8)

Positive *s* values and, therefore, the potential fixation for mutations mediated by natural selection occur when μ_m_ > μ_wt_ (i.e., Δ*Q*_min_ < 0). Multiplying *s*_time_ by the mutant generation time yields a dimensionless selection coefficient that quantifies selection per generation (*34*)

$$s=s_{\text{time}}\frac{\text{ln}2}{\mu_{m}}=-\frac{∆Q_{\text{min}}}{Q_{\text{wt}}}\;\text{ln}2$$

This dimensionless coefficient is more appropriate for assessing the relative importance of natural selection and genetic drift in the evolution of mutations. Notably, there are differences between the values of *s*_time_ and *s*, as well as in the shape of their relationship with *Q*, particularly when *Q* approaches *Q*_min_ (i.e., when generation time is long) (fig. S6). The inclusion of a mortality rate term (*m*), which we assume to be negligible here (*m* = 0 day^−1^), does not affect Eq. 8. However, it would increase *s* values in Eq. 1, thereby making our *s* estimates conservative. This increase in *s* is low when μ_m_ >> *m* and raises markedly when *m* approaches μ_m_. From Eqs. 1 and 4 to 6, the selection coefficients mediated by the effect of P (*s*_P_) and N (*s*_N_) limitation can ultimately be expressed and estimated as a function of the number of P and N atoms affected by the indel. In the case of N limitation, the changes in the histone N requirements associated with indels can also be taken into account when estimating the selection coefficient (*s*_Nh_).

Selection coefficients could be alternatively estimated using the fitness expression provided in Litchman and Klausmeier (*67*). However, this expression is more complex and includes parameters such as the maximum nutrient uptake rate (*V*_max_) and the half-saturation constant for nutrient uptake (*K*), which are typically unavailable for most species. It is important to note that selection coefficients result from both changes in resource requirements (nutrients in this case, *s*_n_) and effects on cellular functioning (*s*_f_), such that $s=s_{n}+s_{f}$ (*7*). The selection coefficients provided assume that functional effects are negligible (i.e., *s*_f_ = 0). Nevertheless, a subset of noncoding regions is functional; indels in these functional regions would likely be deleterious and consequently affect the estimation of *s*.

### Numerical estimation of selection coefficients and comparison with effective population size

Selection coefficients were calculated by using Eq. 1 for species with available *Q*_min_ values for P or N (i.e., *Q*_minP_ or *Q*_minN_). The impact of selective pressures associated with P and N limitation was compared using *s*_P_, *s*_N_, and *s*_Nh_ values. We did not investigate the variations of *s*_Nh_ arising from intrinsic or environmental factors that influence nucleosome number (e.g., the locus of the mutation and environmental stressors) or histone composition (*68*). We focused on mutants with indel mutations ranging from 1 to 1000 nucleotides. A *Q*_wt_ = 1.5 × *Q*_min,wt_ was used in all *s* calculations; however, the effects of varying *Q*_wt_ on *s* values were also examined. A *Q*_wt_ = 1.5 *Q*_min,wt_ represents an intermediate *Q* value that can occur in nutrient-limited cells and yields conservative *s* values, given that *s* could be 30% higher (fig. S6). Any mechanism affecting *Q*_wt_, such as cellular nutrient excretion or the effect of other nutrients on the uptake of the focal limiting nutrient (*58*, *69*), is implicitly accounted for in *Q*_wt_. Some species of cyanobacteria, pointed out with an asterisk in figs. S4 and S5, can use N_2_ as N source. In these species, the selection coefficients estimated from changes in intracellular nitrogen might be less meaningful, as N_2_ fixation makes N limitation less likely and can shift growth limitation toward energy or other nutrients, such as P or Fe (*52*).

The efficiency of natural selection relative to genetic drift in the evolution and fixation of indels was assessed by comparing the selection coefficient with the inverse of the effective population size, 1/*N*_e_ (*8*, *70*). In haploid organisms, the strength of selection and the random effects of genetic drift are of similar magnitude when |*s*| = 1/*N*_e_ (|*s*| = 1/2*N*_e_ for diploids), and selection dominates over drift when |*s*| > 1/*N*_e_. We used selection coefficients obtained from Eq. 1 because, unlike *s*_time_, they are dimensionless and reflect the per-generation strength of selection. *N*_e_ is also dimensionless and refers to the idealized number of individuals that genetically contribute to the next generation; it is estimated from neutral polymorphism and per-generation mutation rates (*71*).

We also estimated the minimal indel length (*l*_min_) at which selection and genetic drift are of equivalent strength for indel mutations occurring in nontranscribed and transcribed regions. For these *l*_min_ calculations, diploidy was assumed for diatom and raphidophyte species as they exhibit diploid vegetative cells (*72*), as well as for *E. gracilis* (*73*). For all other groups, haploidy was assumed as the dominant life stage, although diploid stages have been observed (*72*). Due to the lack of available *N*_e_ estimates for most phytoplankton species, we used a range of *N*_e_ values from 10^6^ to 10^8^, consistent with previous estimates for unicellular algae (table S2). For transcribed regions, *c*_mRNA_ = 1 was assumed on the basis of transcriptomic studies in the yeast *Schizosaccharomyces pombe* (*74*). Last, *l*_min_ values for nontranscribed and transcribed regions were compared with the observed intergenic and intronic length percentiles (1st, 10th, 50th, 90th, and 99th) in species with available General Feature Format (GFF3) annotation files.

### Genome analyses

Genomic features of phytoplankton species were estimated from the genome FASTA and GFF3 files downloaded from the National Center for Biotechnology Information (NCBI). We downloaded 229 FASTA files and 114 GFF3 files, which were not available for all assemblies, corresponding to 177 and 93 different species, respectively. The dataset included cyanobacteria and a wide diversity of eukaryotic lineages, with chlorophytes and diatoms being the most abundant groups (fig. S1 and data S1).

Genome size was inferred from the total number of nucleotides in the genome assemblies, except for *Effrenium voratum*, for which genome size was estimated from the last 3′ position in the GFF3 file. All other genomic features were extracted from the GFF3 annotation files. The number of genes was calculated as the sum of all elements labeled as “gene” or just “CDS” without an associated gene identifier. Pseudogenes were not counted as “genes.” The total coding sequence length was estimated as the sum of the lengths of all nonoverlapping intervals labeled as CDS (excluding pseudogenes), representing protein-coding sequences but excluding untranslated regions. The total length of noncoding sequences was estimated by subtracting the total coding region length from the overall genome size. We also calculated the total, average, and 1st, 10th, 50th, 90th, and 99th percentile sizes of intergenic and intronic region sizes, as well as the number of introns per gene. Intergenic regions were defined as noncoding regions located outside genes, whereas introns were defined as intervals within genes but outside exons (or CDS intervals when exon information was absent). Intron size estimates excluded overlapping intron positions resulting from the alternative splicing variants. All genomic traits were calculated in R version 4.1.0 using the following packages: ape, seqinr, GenomicRanges, and tidygenomics (*75*–*79*). When multiple assemblies or GFF3 files were available for a given species, genomic traits were calculated separately for each, and the median values were used for downstream analyses.

### Minimum quotas and cell volumes datasets

Most of data on cell volumes and minimum quotas (*Q*_minP_ and *Q*_minN_) for phosphorus (P) and nitrogen (N) for different phytoplankton species were compiled from the literature (data S2) (*26*, *31*–*33*). For *Q*_minN_, we focused on measurements using nitrate as the N source, because these data were available for more species than measurements using ammonium. In addition, we supplemented the dataset with our measurements of *Q*_minP_ and *Q*_minN_, for *O. tauri* (RCC4221), *Ostreococcus mediterraneus* (RCC2590), *B. prasinos* (RCC4222), *Micromonas commoda* (RCC827), and *Pyramimonas* sp. (RCC6848) (table S1; see the next section). In the case of cell volumes, we also used the dataset of Finkel *et al*. (*80*). When cell volumes were not available in any of the previous references, we used values reported in other studies (data S3). For *B. prasinos*, *Chlamydomonas reinhardtii*, *Chlamydomonas incerta*, *Chlamydomonas schloesseri*, *E. voratum*, *Haematococcus lacustris*, *Nannochloropsis salina*, *O. mediterraneus*, and *Pseudo-nitzschia multistriata*, cell volumes were estimated from cell dimensions and shapes. The dataset of species cell volumes, including their reference source and estimation procedures, is provided in data S3.

### *Q*_min_ and μ_max_ measurements

We measured *Q*_minP_, *Q*_minN_, μ_maxP_, and μ_maxN_ for *O. tauri* (RCC4221), *O. mediterraneus* (RCC2590), *B. prasinos* (RCC4222), *M. commoda* (RCC827), and *Pyramimonas* sp. (RCC6848). Batch cultures (90 ml) were grown in L1 medium with a reduced concentration of phosphorus or nitrate to induce P or N limitation, respectively. For cultures intended to measure *Q*_minP_, 2 μM P was used instead of 36.2 μM P (N:P ratio = 440), whereas, for cultures used to measure *Q*_minN_, 40 μM N was used instead of 882 μM N (N:P ratio = 1.10). Three replicates were used for each species-nutrient combination. An additional flask with complete L1 medium was included as a control to confirm the occurrence of nutrient limitation. Cultures were inoculated with 20,000 cells ml^−1^, except *Pyramimonas* sp., which was inoculated with 500 cells ml^−1^. A mix of antibiotics was added to prevent bacterial growth (*81*). Cultures were incubated at 20°C under continuous light. Cell counts were performed with a FACSCanto II cytometer [three-laser, eight-color (4-2-2), BD-Biosciences] equipped with a 20-mW 488-nm coherent sapphire solid-state blue laser. Sample volumes and cell concentrations were determined using Becton-Dickinson Trucount beads. Phytoplankton were enumerated on the basis of side scatter and red fluorescence (>670 nm) from chlorophyll pigments. Data were acquired with BD FACSDiva software (BD Biosciences).

Daily intrinsic growth rates (μ; per day) were estimated assuming exponential growth

$$\mu =\frac{1}{∆t}\;\text{ln}\left(\frac{N_{t}}{N_{0}}\right)$$ (9)

where *N*_t_ and *N*_0_ denote cell concentrations (cells per milliliter) at the end and the beginning of the time interval, respectively. Aliquots (10 ml) were filtered when cultures reached the stationary phase, defined as the first or second day when the average μ of the three replicates was <0.10 day^−1^ (fig. S2). Filtration was performed using 25-mm precombusted glass fiber filters of type GF/F (type GF/A filters were used for *Pyramimonas* sp.) that were washed with 10 ml of artificial seawater (ASW). Blanks consisted of precombusted filters washed with 10 ml of ASW. Filters were digested in boric acid and potassium persulfate (*82*), and dissolved inorganic P and N in the digested solution were measured using a SkalarSA5000 autoanalyzer. The *Q*_minP_ and *Q*_minN_ (femtomoles per cell) were estimated from the nutrient content (femtomoles of P or N, respectively) of the digested culture solution (*Nut*_c_) after subtracting the amount of nutrient in the digested blank solution (*Nut*_b_) (table S1)

$$Q_{\text{min}}=\frac{{\mathrm{Nut}}_{c}-{\mathrm{Nut}}_{b}}{N\;V}$$ (10)

where *N* is the cell concentration in the culture and *V* is the filtered culture volume (milliliters).

The maximum intrinsic growth rates μ_maxP_ and μ_maxN_ were estimated from cultures in which P and N were the limiting nutrients, respectively. Regression slopes of the natural log-transformed cell abundance against time during the exponential growth phase were calculated for each replicate, and the resulting slope values were averaged (table S1).

### Analyses of the relationship between genome size, *Q*_min_, and cell volume

Datasets containing genomic region lengths (data S1), *Q*_min_ (data S2), and cell volume (data S3) were merged at the species level to analyze the relationship between genomic and phenotypic traits. Synonyms (homotypic names) were identified using AlgaeBase, and the *Q*_min_ values measured for strains labeled as *Selenastrum capricornutum* were merged with genomic region lengths from *Raphidocelis subcapitata*, as these strains were frequently misnamed and actually correspond to *R*. *subcapitata* (*83*). Median values were used for species with multiple observations. For species lacking phenotypic or genomic data, median genus-level values were used when available.

Based on the N and P content of nucleotides, we hypothesize the occurrence of positive correlations between genomic traits and *Q*_min_. These correlations could arise through two nonexclusive mechanisms, which imply opposite directions of causation. On the one hand, the interspecific variation in *Q*_min_ may be a consequence of differences in the size of DNA regions. All else being equal, species with short DNA regions necessarily contain less P and N in DNA, which ultimately contributes to saving nutrients and maintaining a low *Q*_min_. On the other hand, according to Eq. 1, interspecific changes in *Q*_min_ may drive changes in the size of DNA regions. In species with low *Q*_min_, selection will be more efficient to maintain short DNA regions, as a given silent deletion or insertion would experience stronger selection pressure, either positive or negative, respectively, than in species with high *Q*_min_. These two mechanisms are both mediated by the N and P content of nucleotides and can be seen as two sides of the same coin. To remove the direct effect of DNA region size on *Q*_min_ and assess the occurrence of the second mechanism (impact of *Q*_min_ on DNA length), we estimated the relationship between the size of DNA regions and *Q*_min_ after subtracting the P and N content attributable to the DNA region itself.

To analyze the relationship between genome size (and the sizes of the different genome compartments) and cell volume, we hypothesized that the relationship is largely noncausal and arises primarily from the correlation between *Q*_min_ and cell volume (*26*). To test this hypothesis, we estimated the proportion of variation in the size of each DNA region that was exclusively associated with cell volume, uniquely associated with *Q*_minP_ and *Q*_minN_, or shared between *Q*_min_ and cell volume (i.e., variation not attributable solely to *Q*_min_ or cell volume) according to Legendre and Legendre (*84*). A high proportion of variation explained exclusively by cell volume would suggest the influence of additional factors, independent of *Q*_min_, on the relationship between cell volume and genome size, thereby contradicting the hypothesis that *Q*_min_ is the primary driver of the *Q*_min_–genome size relationship. In contrast, a high proportion of shared variation between *Q*_min_ and cell volume would support the hypothesis that *Q*_min_ plays a key role in the genome size–cell volume relationship. However, this would not rule out a possible direct influence of cell volume on genome size. The partial regression analysis required models including *Q*_minN_, *Q*_minP_, and cell volume as predictors, which increased model complexity and reduced sample size compared with regressions performed for each variable separately. Consequently, this analysis was restricted to pooled eukaryotic species, resulting in 30 observations for genome size and 21 observations for the other DNA regions.

The relationships between the lengths of genomic traits and both *Q*_min_ and cell volume were analyzed using log_10_-transformed variables. All reported slope values and statistical results are derived from log-log regressions. Standardized major axis (SMA) regression was used instead of ordinary least squares as SMA provides symmetric regression lines that do not assume a strict dependent-independent relationship. This approach is particularly appropriate when analyzing the relationships between the lengths of genomic traits and *Q*_min_ as the causal relationship may operate in both directions: *Q*_min_ may influence genomic trait length, and vice versa. Therefore, either variable can be treated as dependent or independent. In addition, both *Q*_min_ and genomic traits were treated as random variables; i.e., they were not fixed by experimental design (*85*). Relationships were analyzed separately for prokaryotes and eukaryotes by including domain as a factor in the regression models. We also tested for differences in slopes between domains. Similarly, relationships were analyzed separately for diatoms and chlorophytes as these groups had more than four observations. The same approach was applied to analyze the relationship between genome size and the size of genome compartments.

All data analyses and visualizations were performed in R version 4.1.0 using the packages dplyr, tidyr, stringr, ggplot2, ggforce, ggh4x, patchwork, and sp (*75*, *86*–*93*). SMA regression models were fitted using the smatr R package (*94*). Figures were edited with Inkscape as needed for minor adjustments.

### Comparison of the nutrient and bioenergetic cost estimations of indels

Lynch and Marinov (*7*) quantified the fraction of the cellular energy budget required to replicate an average-sized gene in diverse prokaryotic and eukaryotic species. To compare these values with our estimates of fractional costs in terms of N and P, we extracted the fitted values from figure 4 of (*7*). These fitted values were estimated from maximum values of fractional energy replication cost, which occur when cellular maintenance energy requirements are minimal, i.e., under conditions of maximal growth rate and minimal cell-division time (*7*). For average-sized genes, these estimates include the costs of synthesizing nucleotides and, in eukaryotes, the amino acids in histone proteins incorporated into nucleosomes. These fractional costs are similar for noncoding indels of equivalent size, because transcription and translation costs are not considered. Analogously, we estimated the fractional P and N costs of average-sized genes relative to *Q*_minP_ and *Q*_minN_ for species with available GFF3 annotation files and *Q*_min_ values. Equations 4 to 6 were used to estimate the P and N content of average-sized genes, assuming *c*_mRNA_ = 0 and using the GC content of the entire genome. The resulting values were divided by the *Q*_min_ of the corresponding species. This approach relies on two assumptions: (i) Cells are strongly nutrient limited (*Q* close to *Q*_min_), which leads to maximal estimations of fractional nutrient costs; and (ii) maintenance costs in terms of N and P are either negligible or similar in DNA and other biomolecules, leaving the ratio unaffected. Low maintenance costs are supported by the low cellular release of growth-limiting nutrients in phytoplankton. For nitrogen, this release is below 10% of the nitrogen taken up (*95*). Unlike energy, cells recycle nutrients (i.e., materials), reallocating them into various biomolecules throughout their life span. For the fractional N cost, estimates were made considering either only the N in the nucleotides or both the N in nucleotides and histones, using the same methodology used in *s*_N_ and *s*_Nh_ calculations. Subsequently, SMA regressions were fitted to investigate the relationship between fractional N and P costs and cell volume.

Last, the fitted values for nutrient and energetic fractional costs of average-sized genes, which in both cases correspond to maximum values, were compared for eukaryotic species of similar volumes (fig. S15). This comparison was used to assess the relative strength of natural selection mediated by energy limitation (light in photoautotrophs) and nutrient constraints acting on indels in noncoding regions of phytoplankton genomes. Given that these analyses are based on maximum fractional energetic costs, the inferred strength of selection mediated by nutrients relative to energy is likely to be conservative under resource-limiting conditions and suboptimal growth rates.

### Testing the relationship between intragenomic noncoding region lengths and transcription rates

To test the relationship between expression level and noncoding region length within genomes, available transcriptomes were mapped against the *B. prasinos* and *O. tauri* genomes with bwa-mem2 and bam files sorted and analyzed using samtools (*96*, *97*). The NCBI accession numbers are SRR14396982 for *B. prasinos* (*98*) and SRR5986283 to SRR5986292 for *O. tauri* (*99*). These transcriptomes were obtained from cultures grown in L1 medium at 20°C. To quantify coverage across intergenic regions and exons, we extracted the positions from GFF files and estimated read coverage with samtools depth (using option -a to include sites with zero coverage). Both median and average coverage of sites per interval were imported into R for downstream analyses. Intergenic and intronic regions were grouped in quartiles based on their median read coverage per position, which we used as a proxy for their level of expression. Differences in the sizes of intergenic and intronic regions across quartiles were tested using Wilcoxon rank sum tests.

### Screening metagenomes to extract and analyze indel polymorphisms in natural populations

Raw reads from 52 TARA metagenomes with a higher prevalence of Mamiellales and concomitant environmental nutrient concentrations available (*48*, *49*, *100*, *101*) were mapped to an assemblage of the following reference genomes: *M. commoda* (GCF_000090985.2), *Micromonas pusilla* (GCF_000151265.4), *O. lucimarinus* (GCF_000092065.1), *O. tauri* (GCF_000214015.3), *O. mediterraneus* (GCA_012295225.1), and *B. prasinos* (GCF_002220235.1). Raw reads were aligned to a concatenated reference FASTA file with hisat2 (*102*). Resulting BAM files were processed and sorted with *samtools* and variant calling was performed using HaplotypeCaller from Genome Analysis Toolkit (GATK) (*97*, *103*). Indel variants were extracted from the resulting VCF files using the awk Unix command. Positions with two alternative indel alleles were discarded to eliminate potential polymorphisms resulting from misalignment. Indels supported by only 1 read were excluded to avoid sequencing errors. The remaining 210,675 indels were annotated with SnpEff (*104*).

The resulting file was further processed to select relevant lines and columns and imported into R for downstream statistical analyses. Briefly, the MAF at each indel position was estimated from the read coverage. The higher frequency variant was considered the ancestral allele, and the classification of the variant as a deletion or an insertion was determined by comparing the lengths of the two alleles: Deletions correspond to the MAF allele with the shorter length, whereas insertions correspond to the MAF allele with the longer length. This is as expected for neutral polymorphisms in a panmictic population of constant size, assuming free recombination, an infinite sites model, generated by de novo mutations. In a sample of *n* (*n* ≥ 3) individuals, the proportion of polymorphisms segregating at frequencies equal or lower than 50% is given by $\sum_{i=1}^{\left(n-1\right)/2}\frac{1}{i}$/$\sum_{i=1}^{n-1}\frac{1}{i},$ and this proportion constitutes the majority of cases (*46*).

We restricted the analysis to polymorphisms (i) with read coverage equal to or higher than 20, (ii) present in only one metagenome, and (iii) located in intergenic regions (data S4). The threshold of 20 reads was chosen to increase the precision of the MAF estimates. By focusing on polymorphisms present in only one metagenome, we avoided statistical issues arising from multiple inclusion of the same observation in comparisons.

Indels were categorized into two groups based on length: short, <10 bp; and long, ≥10 bp. Environmental phosphate or nitrate plus nitrite concentrations for each metagenome were used to classify indels into “low” and “high” nutrient availability categories in such a way that the number of indels was as balanced as possible while ensuring that indels from any single metagenome were not split into different categories. Statistical analyses based on phosphate and nitrate plus nitrite concentrations were performed separately using R. Differences in MAF between categories based on indel type, indel length, or nutrient level (i.e., simple effects) were tested using Wilcoxon rank sum tests. Data were analyzed by pooling indels from all species together, as well as for *B. prasinos* and *O. lucimarinus* separately. Analyses were not performed separately for the other species due to the low number of long indels. We repeated the analyses using median MAF estimated for each metagenome, which were grouped into low and high nutrient categories. Note that, in this case, an equal number of metagenomes were classified into low and high nutrients categories. The number of observations was lower in this analysis as it was constrained by the number of metagenomes.

## Supplementary Materials

### This PDF file includes:

Figs. S1 to S15 Tables S1 to S9 Data S1 to S4 References
