# Supplementary material for: Effect of cellular nutrient economy on the evolution of genome size in phytoplankton
Source: Sci Adv. 2026 Jul 8;12(28):eaee2207. doi: 10.1126/sciadv.aee2207 (PMC13344279; doi:10.1126/sciadv.aee2207)
Supplement: Supplementary file 1 — Figs. S1 to S15 Tables S1 to S9 Data S1 to S4 References [file sciadv.aee2207_sm.pdf]

Science Advances
AAAS

Supplementary Materials for

Effect of cellular nutrient economy on the evolution of genome size in phytoplankton

Carlos Caceres *et al.*

Corresponding author: Carlos Caceres, carlos.caceres-perez@univ-perp.fr;
Gwenael Piganeau, gwenael.piganeau@obs-banyuls.fr

Sci. Adv. **12**, eaee2207 (2026)
DOI: 10.1126/sciadv.aee2207

This PDF file includes:

Figs. S1 to S15
Tables S1 to S9
Data S1 to S4
References

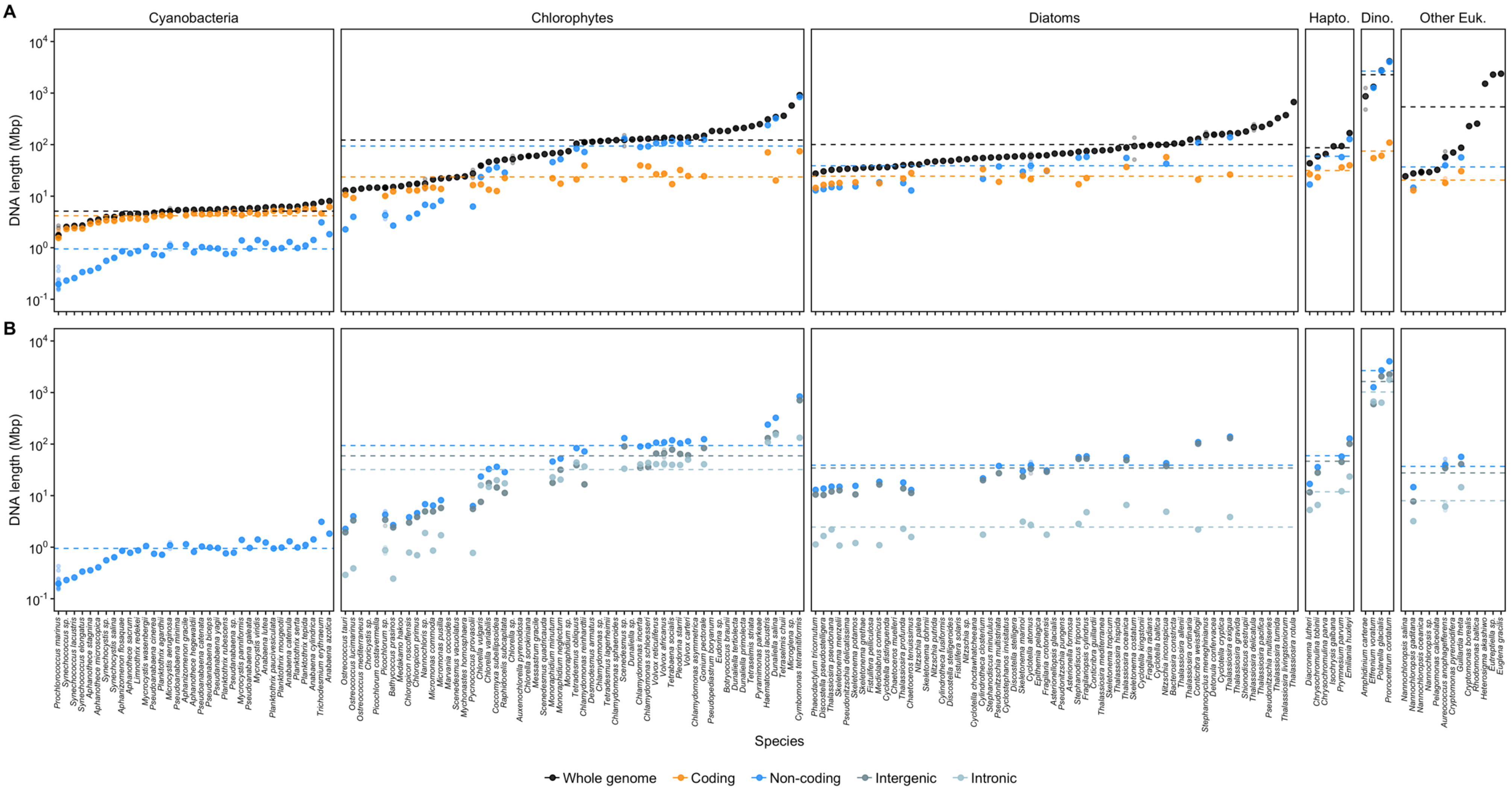

Fig. S1. Total DNA length of different genomic regions in phytoplankton species. (A) Genome, total coding and total non-coding lengths. **(B)** Total lengths of non-coding regions, subdivided into intergenic and intronic regions. Large points indicate median values per species, while small points indicate values at the strain level. Dashed lines show mean values for each group.

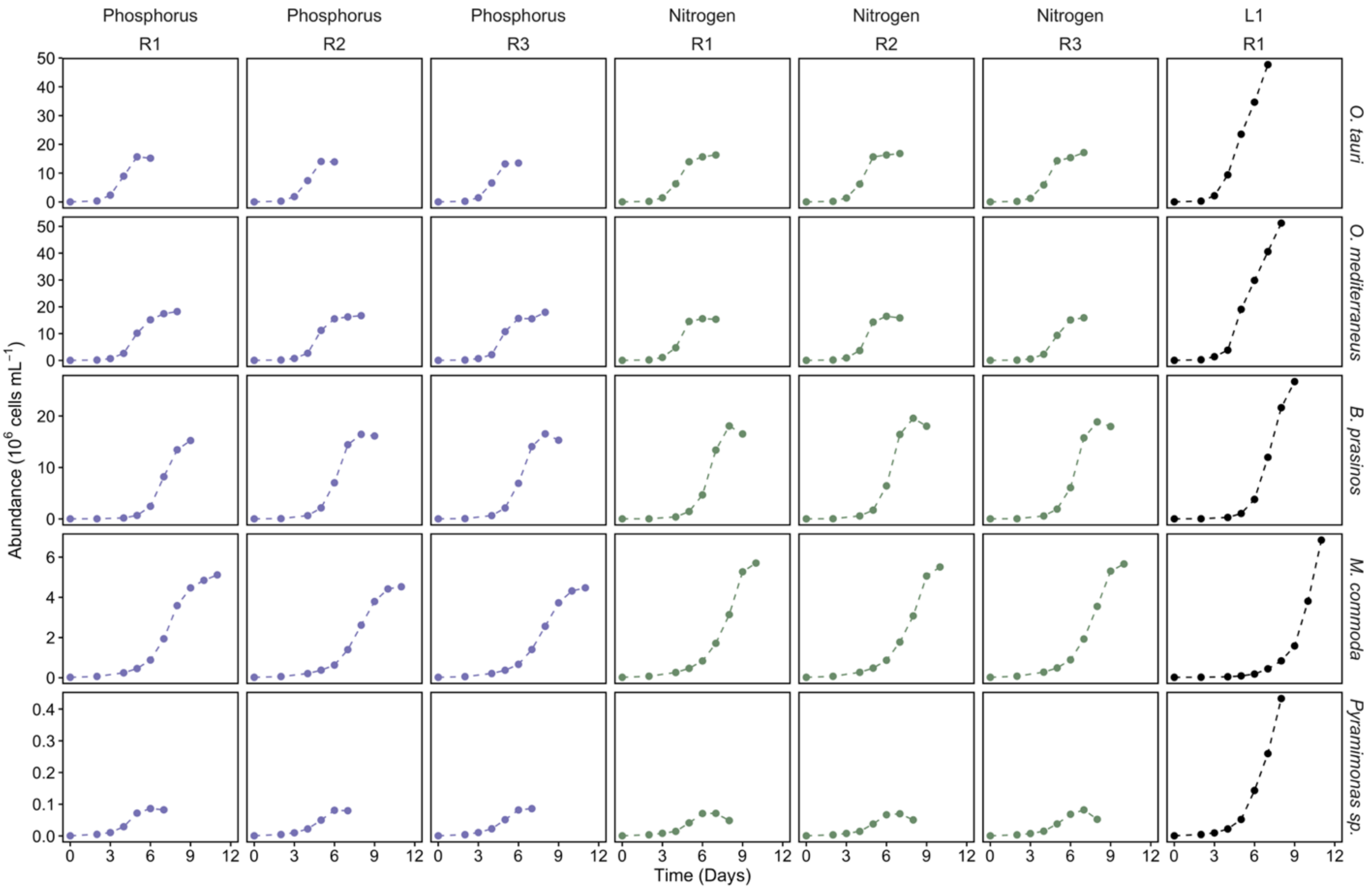

Fig. S2. Cell abundance over time in cultures of five phytoplankton species employed to measure maximum growth rate (μ_{max}) and minimum cellular quotas (Q_{min}) for phosphorus and nitrogen. Each panel shows data for a different species, nutrient treatment, and replicate. The Q_{min} was measured on the final day of cell counts immediately after enumerating. Phosphorus and nitrogen panels correspond to cultures grown under low P (2µM) and low N (40µM) conditions relative to L1 medium, representing P- and N-limited cultures at the stationary phase. Panels labeled 'L1' show cultures grown in complete L1 medium (36.2µM P, 882µM N), included as controls to confirm nutrient limitation in low-P and low-N treatments.

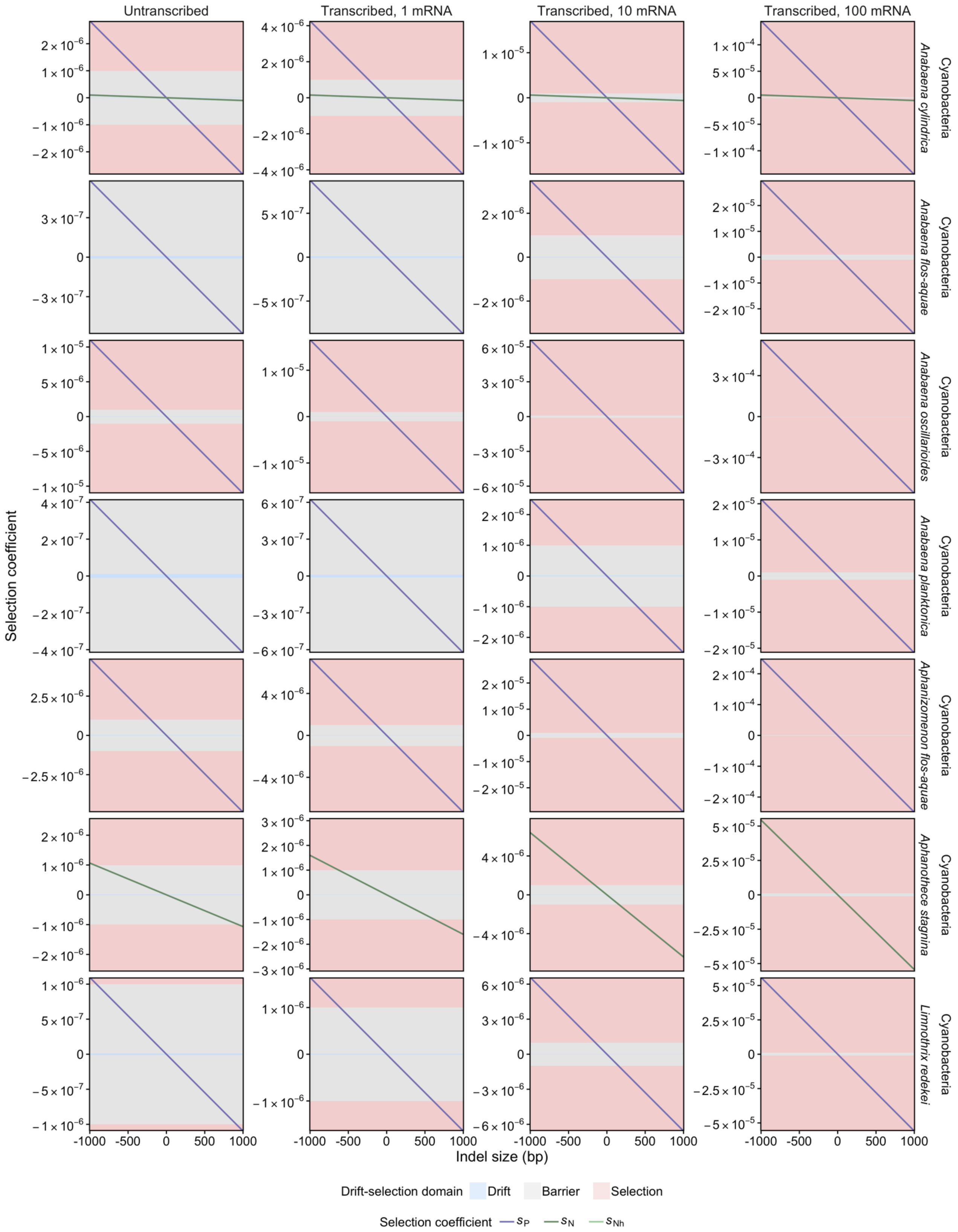

Untranscribed
Transcribed, 1 mRNA
Transcribed, 10 mRNA
Transcribed, 100 mRNA
Cyanobacteria Anabaena cylindrica
Cyanobacteria Anabaena flos-aquae
Cyanobacteria Anabaena oscillarioides
Cyanobacteria Anabaena planktonica
Cyanobacteria Aphanizomenon flos-aquae
Cyanobacteria Aphanothece stagnina
Cyanobacteria Limnothrix redekei
Selection coefficient
Indel size (bp)
Drift-selection domain
Drift
Barrier
Selection
Selection coefficient
s_P
s_N
s_{Nh}

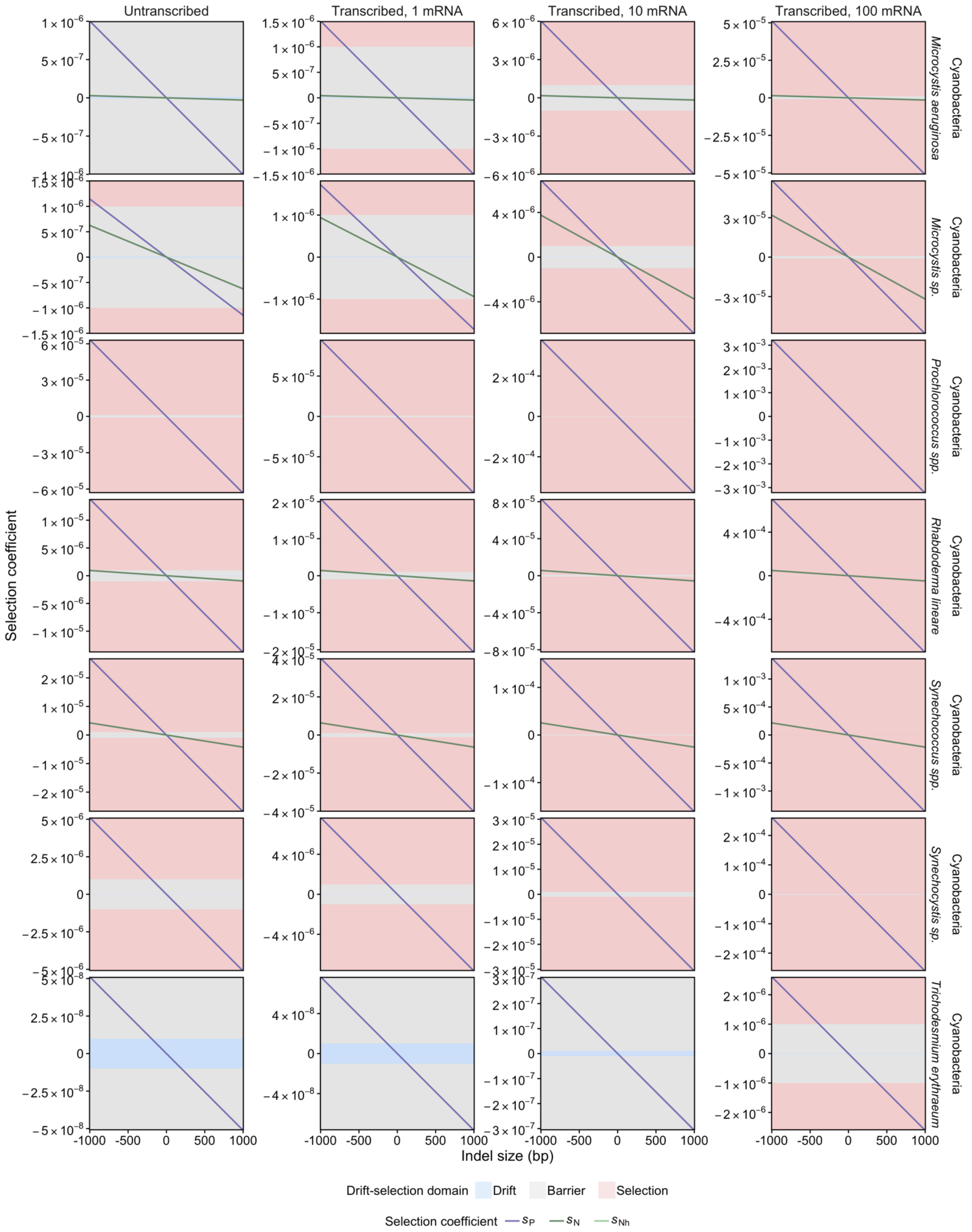

Untranscribed
Transcribed, 1 mRNA
Transcribed, 10 mRNA
Transcribed, 100 mRNA
Cyanobacteria Microcystis aeruginosa
Cyanobacteria Microcystis sp.
Cyanobacteria Prochlorococcus spp.
Cyanobacteria Rhabdoderma lineare
Cyanobacteria Synechococcus spp.
Cyanobacteria Synechocystis sp.
Cyanobacteria Trichodesmium erythraeum
Selection coefficient
Indel size (bp)
Drift-selection domain
Drift
Barrier
Selection
Selection coefficient
s_P
s_N
s_{Nh}

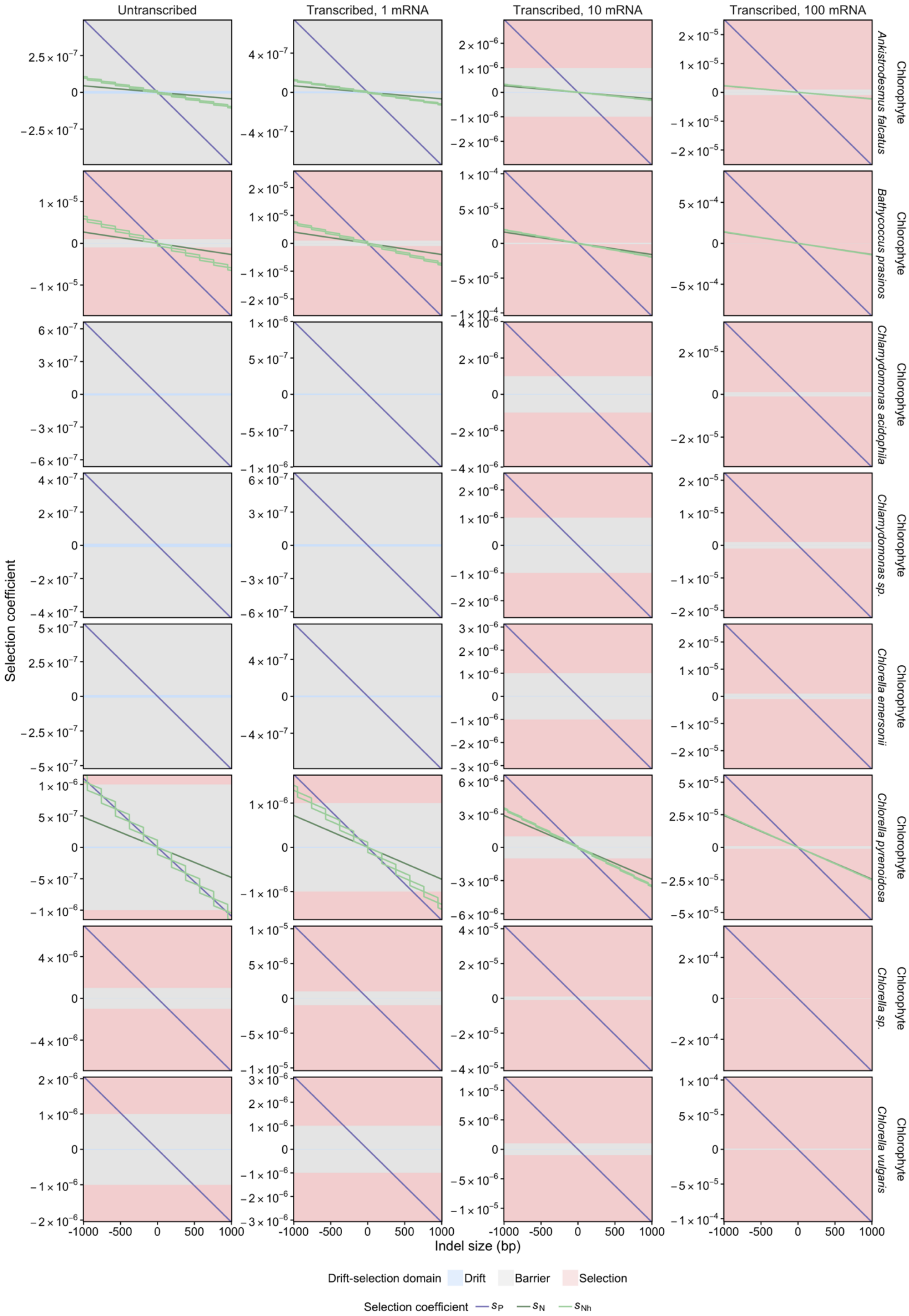

Untranscribed
Transcribed, 1 mRNA
Transcribed, 10 mRNA
Transcribed, 100 mRNA
Chlorophyte
Ankistrodesmus falcatus
Bathycoccus prasinos
Chlamydomonas acidophila
Chlamydomonas sp.
Chlorella emersonii
Chlorella pyrenoidosa
Chlorella sp.
Chlorella vulgaris
Selection coefficient
Indel size (bp)
Drift-selection domain
Drift
Barrier
Selection
Selection coefficient
s_P
s_N
s_{Nh}

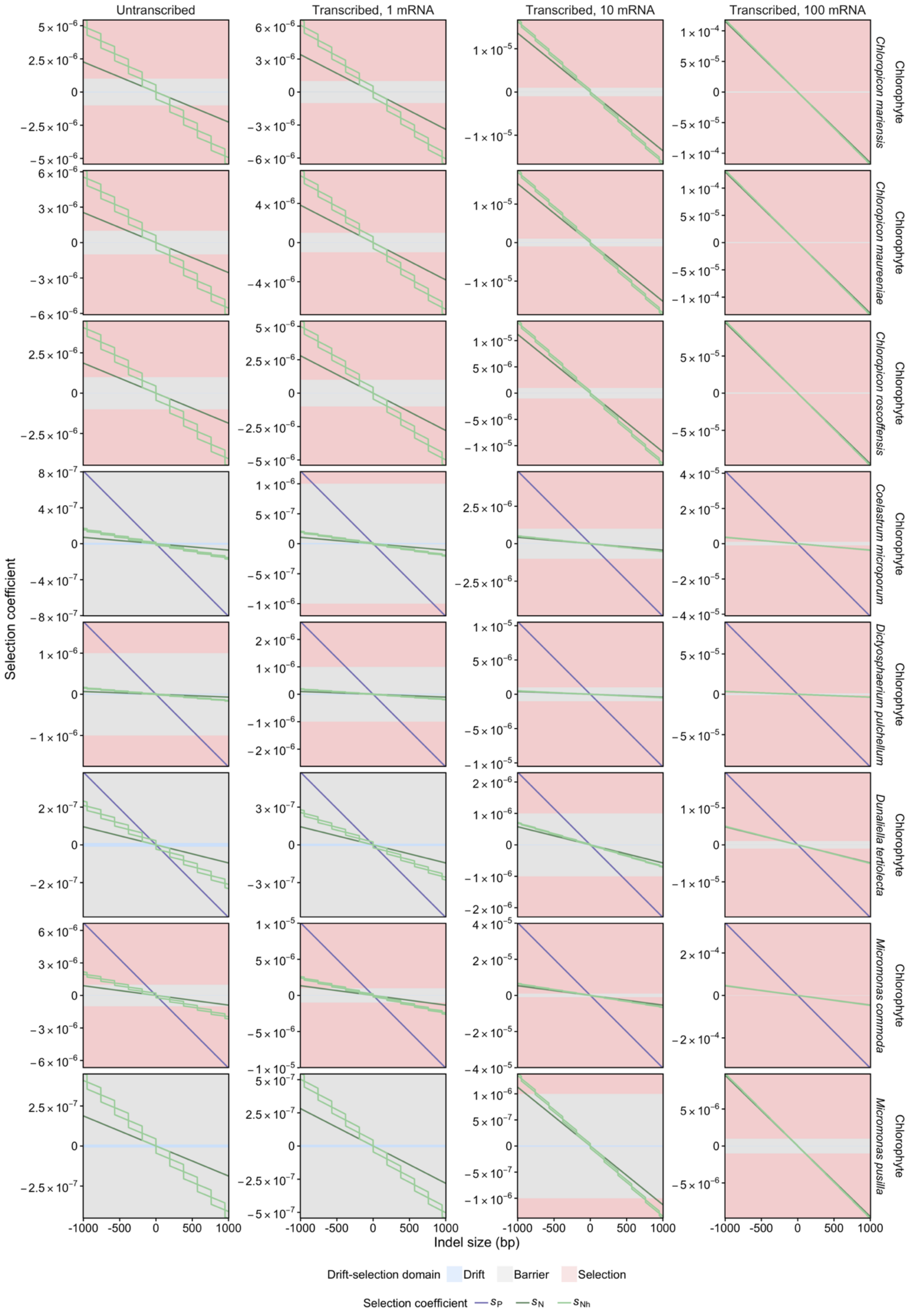

Untranscribed
Transcribed, 1 mRNA
Transcribed, 10 mRNA
Transcribed, 100 mRNA
Chlorophyte
Chloropicon mariensis
Chloropicon maureeniae
Chloropicon roscoffensis
Coelastrum microporum
Dictyosphaerium pulchellum
Dunaliella tertiolecta
Micromonas commoda
Micromonas pusilla
Selection coefficient
Indel size (bp)
Drift-selection domain
Drift
Barrier
Selection
Selection coefficient
s_P
s_N
s_{Nh}

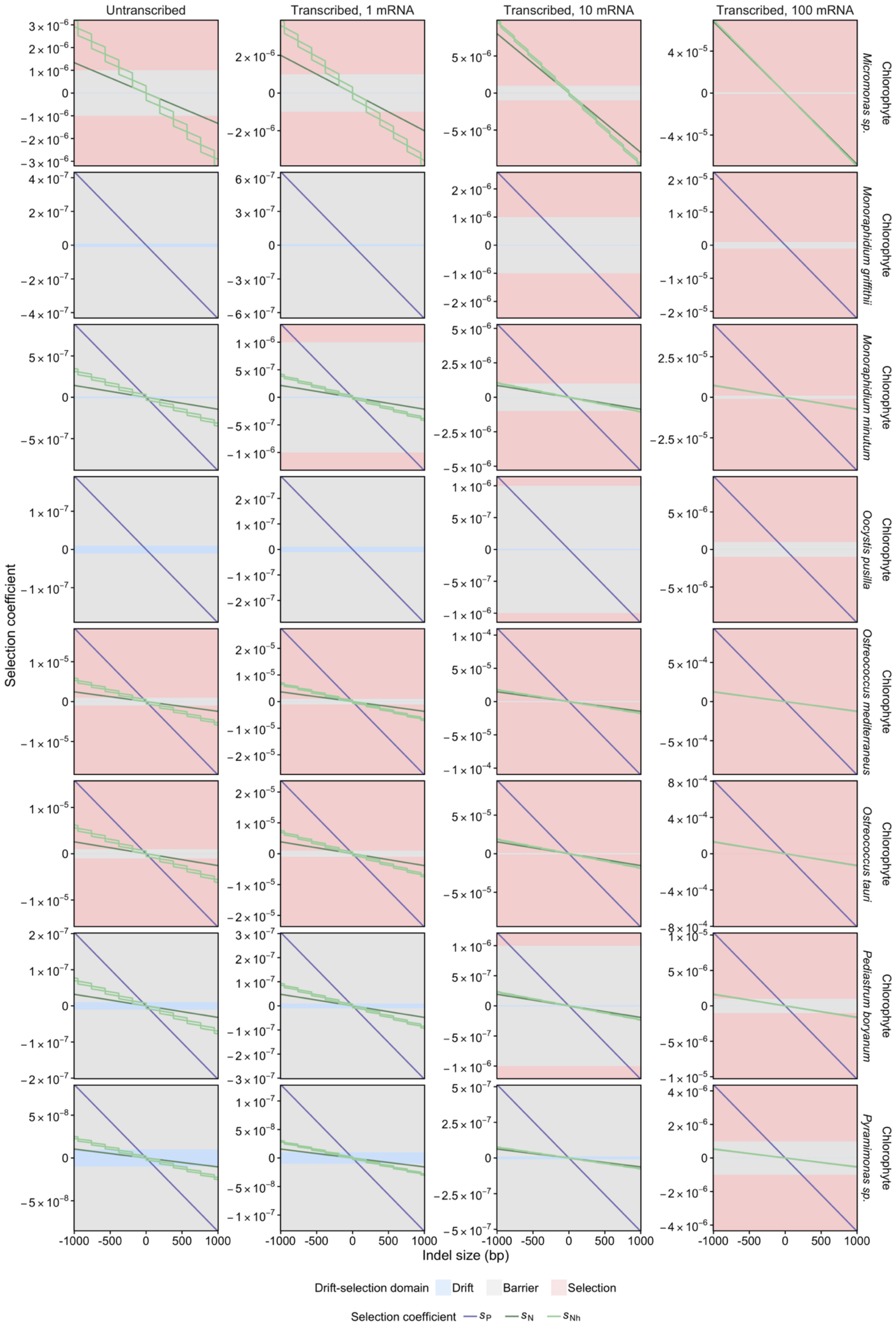

Untranscribed
Transcribed, 1 mRNA
Transcribed, 10 mRNA
Transcribed, 100 mRNA
Chlorophyte
Micromonas sp.
Chlorophyte
Monoraphidium griffithii
Chlorophyte
Monoraphidium minutum
Chlorophyte
Oocystis pusilla
Chlorophyte
Ostreococcus mediterraneus
Chlorophyte
Ostreococcus tauri
Chlorophyte
Pediastrum boryanum
Chlorophyte
Pyramimonas sp.
Selection coefficient
Indel size (bp)
-1000 -500 0 500 1000
Drift-selection domain
Drift
Barrier
Selection
Selection coefficient
s_P
s_N
s_{Nh}

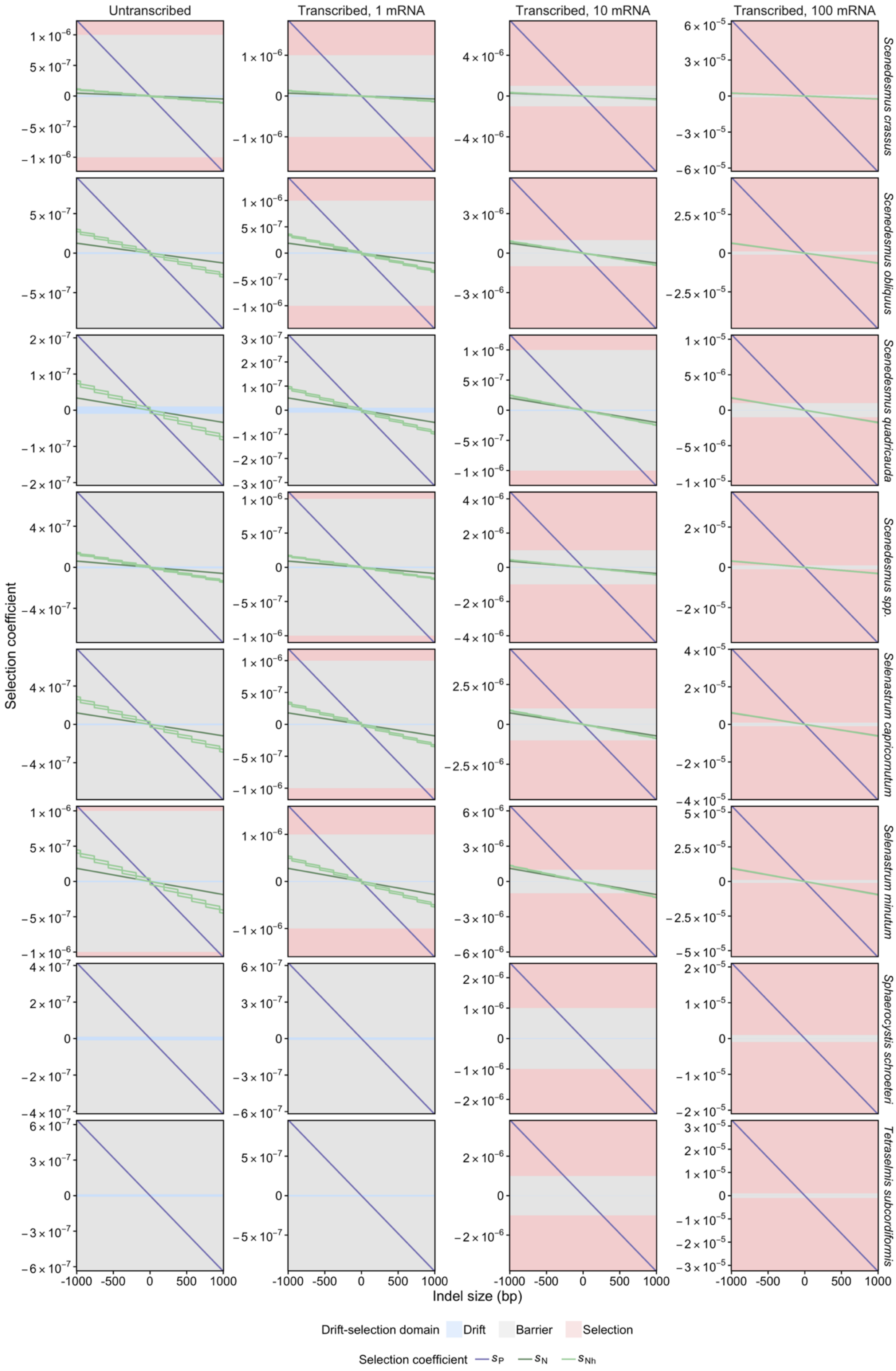

Untranscribed
Transcribed, 1 mRNA
Transcribed, 10 mRNA
Transcribed, 100 mRNA
Scenedesmus crassus
Scenedesmus obliquus
Scenedesmus quadricauda
Scenedesmus spp.
Selenastrum capricornutum
Selenastrum minutum
Sphaerocystis schroeteri
Tetraselmis subcordiformis
Chlorophyte
Selection coefficient
Indel size (bp)
Drift-selection domain
Drift
Barrier
Selection
Selection coefficient
s_P
s_N
s_{Nh}

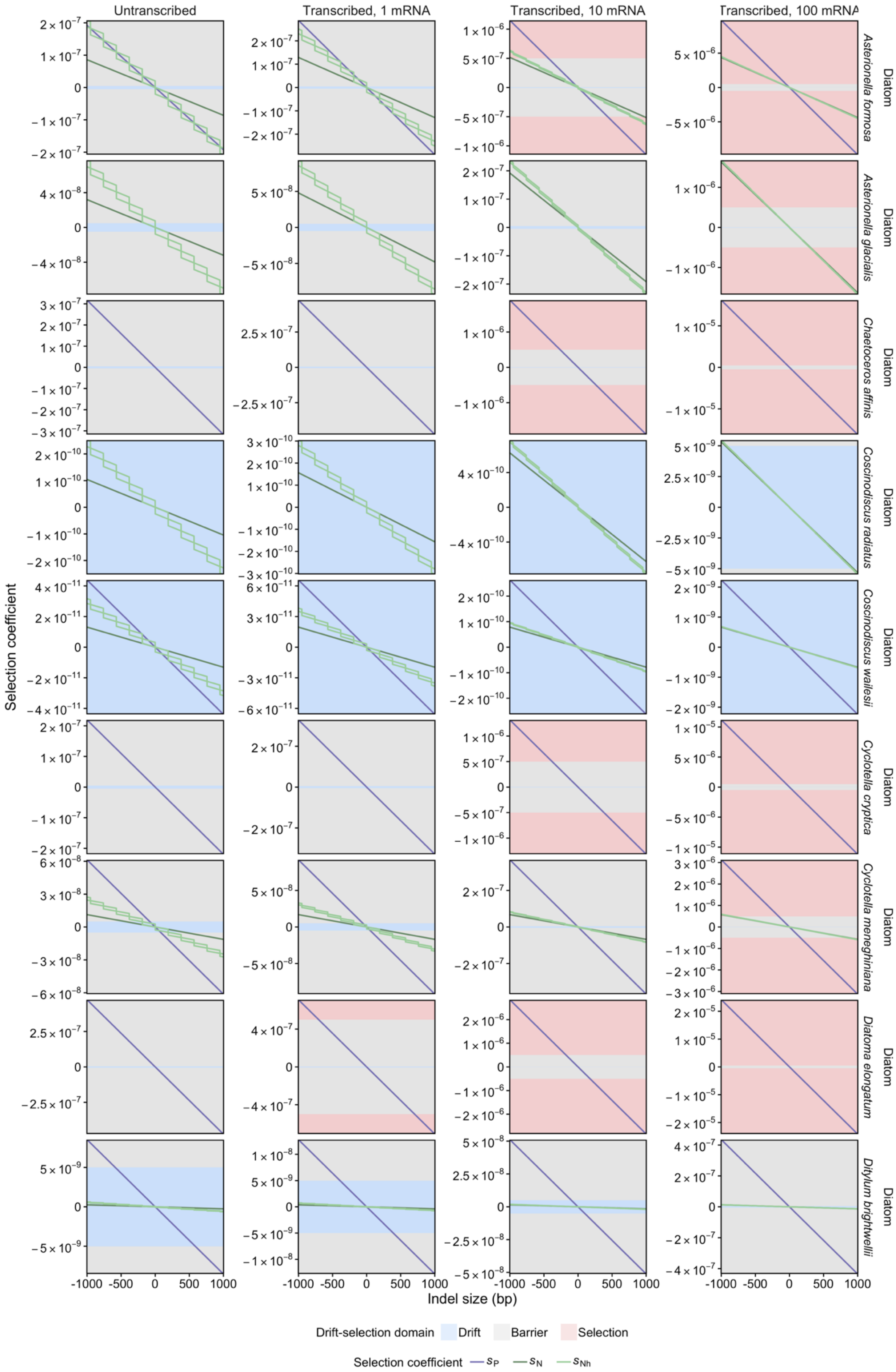
Untranscribed
Transcribed, 1 mRNA
Transcribed, 10 mRNA
Transcribed, 100 mRNA
Diatom
Asterionella formosa
Diatom
Asterionella glacialis
Diatom
Chaetoceros affinis
Diatom
Coscinodiscus radiatus
Diatom
Coscinodiscus wailesii
Diatom
Cyclotella cryptica
Diatom
Cyclotella meneghiniana
Diatom
Diatoma elongatum
Diatom
Ditylum brightwellii
Selection coefficient
Indel size (bp)
Drift-selection domain
Drift
Barrier
Selection
Selection coefficient
s_P
s_N
s_{Nh}

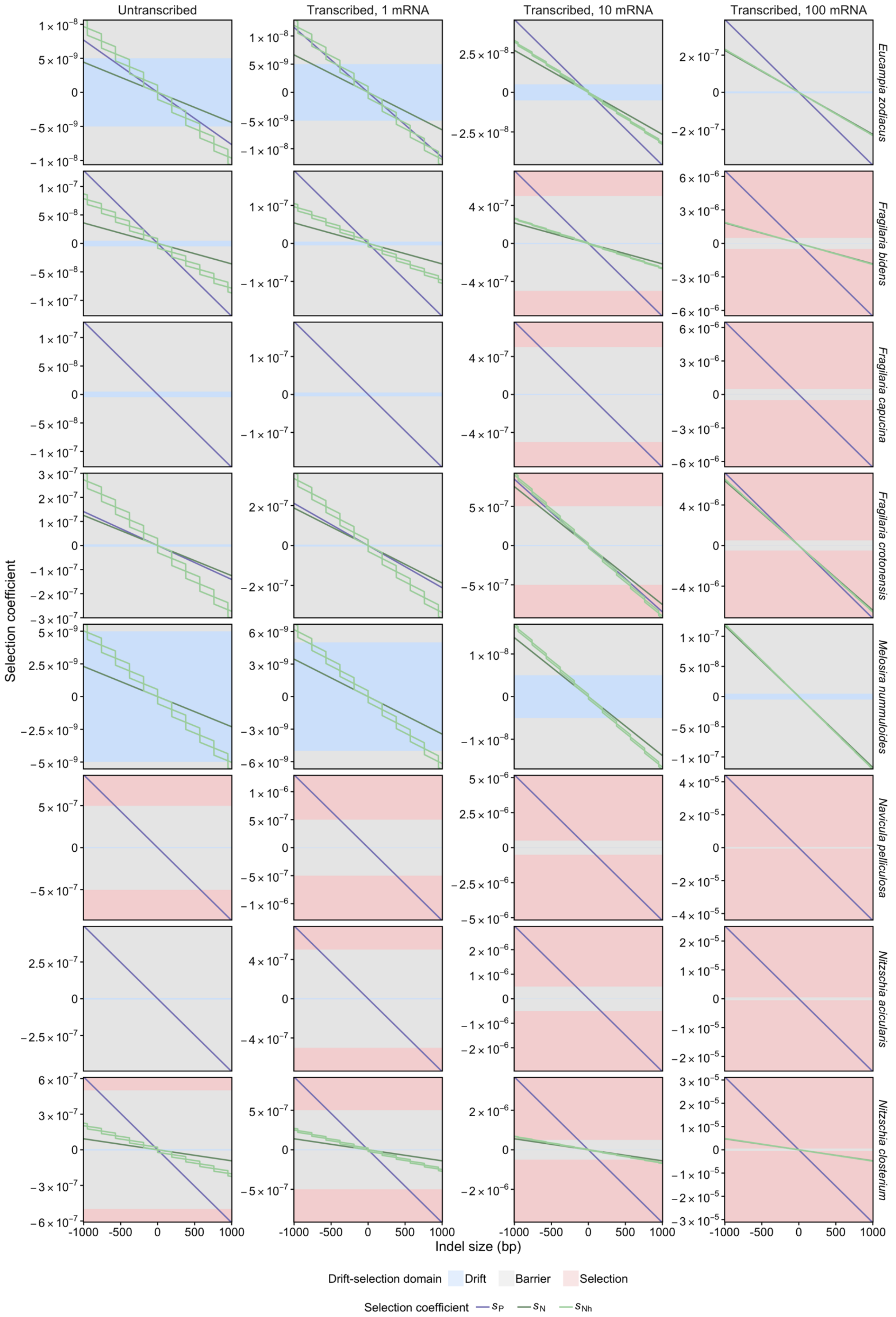

Untranscribed
Transcribed, 1 mRNA
Transcribed, 10 mRNA
Transcribed, 100 mRNA
Diatom Eucampia zodiacus
Diatom Fragilaria bidens
Diatom Fragilaria capucina
Diatom Fragilaria crotonensis
Diatom Melosira nummuloides
Diatom Navicula pelliculosa
Diatom Nitzschia acicularis
Diatom Nitzschia closterium
Selection coefficient
Indel size (bp)
Drift-selection domain
Drift
Barrier
Selection
Selection coefficient
s_P
s_N
s_{Nh}

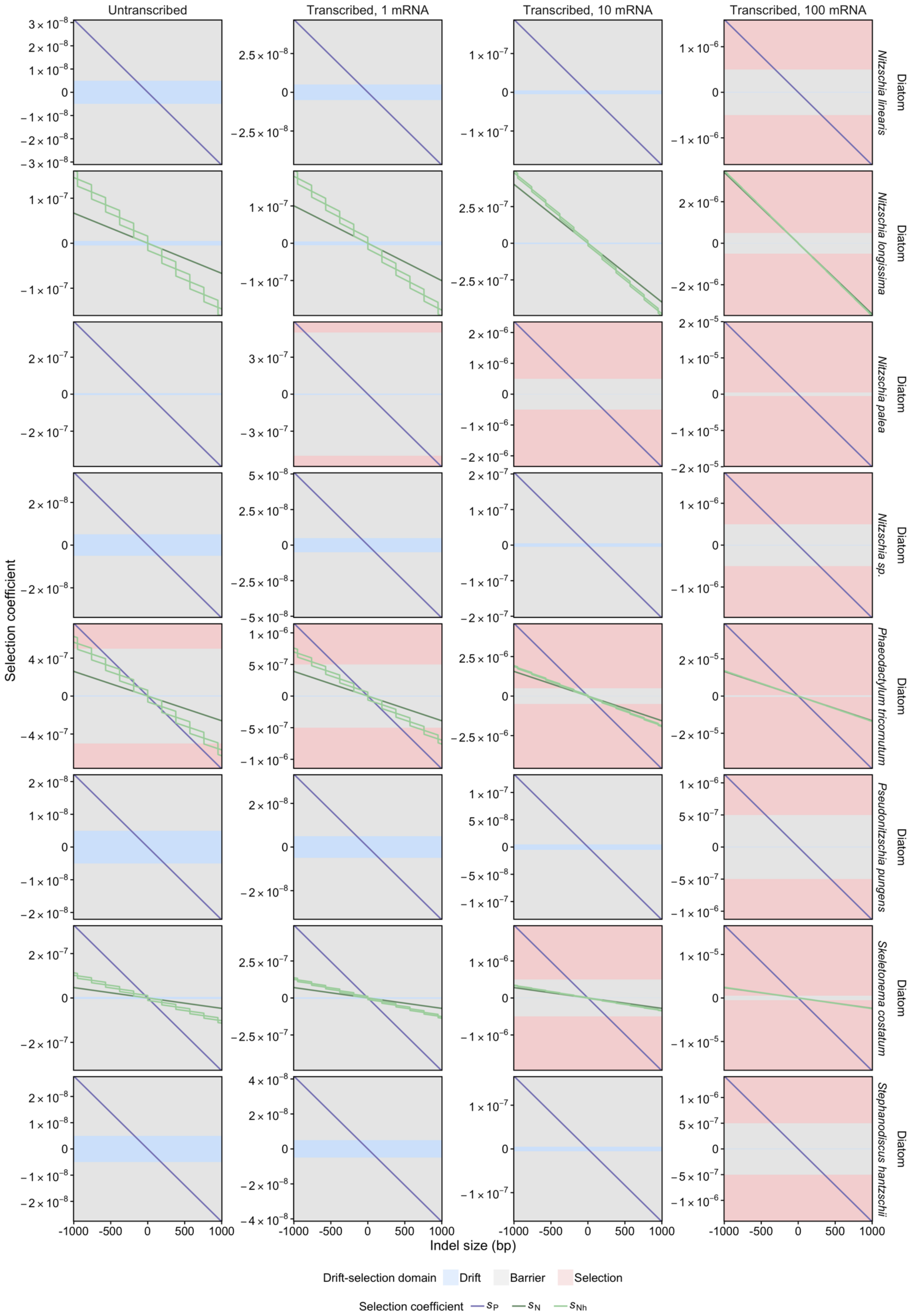

Untranscribed
Transcribed, 1 mRNA
Transcribed, 10 mRNA
Transcribed, 100 mRNA
Diatom
Nitzschia linearis
Nitzschia longissima
Nitzschia palea
Nitzschia sp.
Phaeodactylum tricornutum
Pseudonitzschia pungens
Skeletonema costatum
Stephanodiscus hantzschii
Selection coefficient
Indel size (bp)
-1000
-500
0
500
1000
Drift-selection domain
Drift
Barrier
Selection
Selection coefficient
s_P
s_N
s_{Nh}

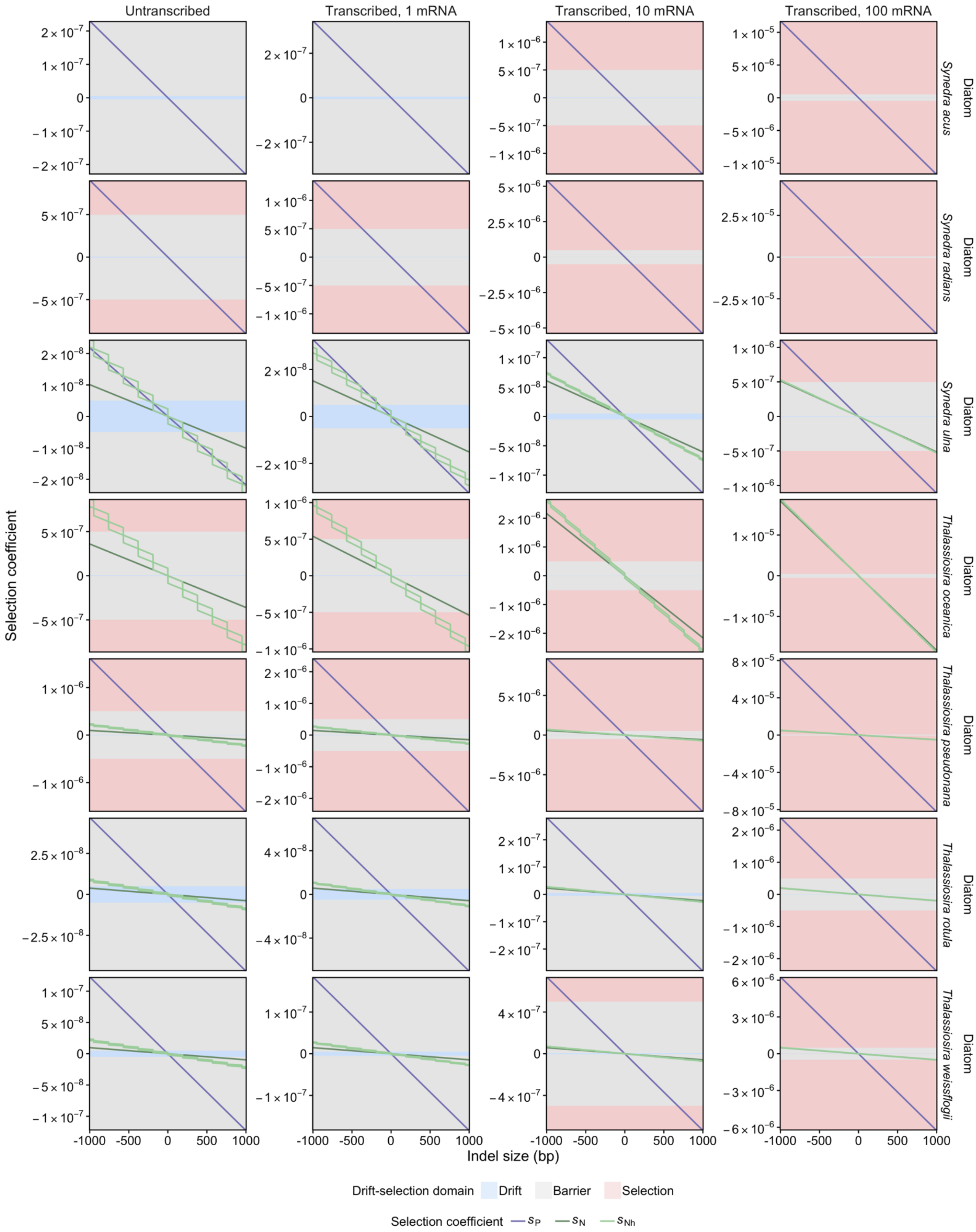
Untranscribed
Transcribed, 1 mRNA
Transcribed, 10 mRNA
Transcribed, 100 mRNA
Diatom Synedra acus
Diatom Synedra radians
Diatom Synedra ulna
Diatom Thalassiosira oceanica
Diatom Thalassiosira pseudonana
Diatom Thalassiosira rotula
Diatom Thalassiosira weissflogii
Selection coefficient
Indel size (bp)
Drift-selection domain
Drift
Barrier
Selection
Selection coefficient
s_P
s_N
s_{Nh}

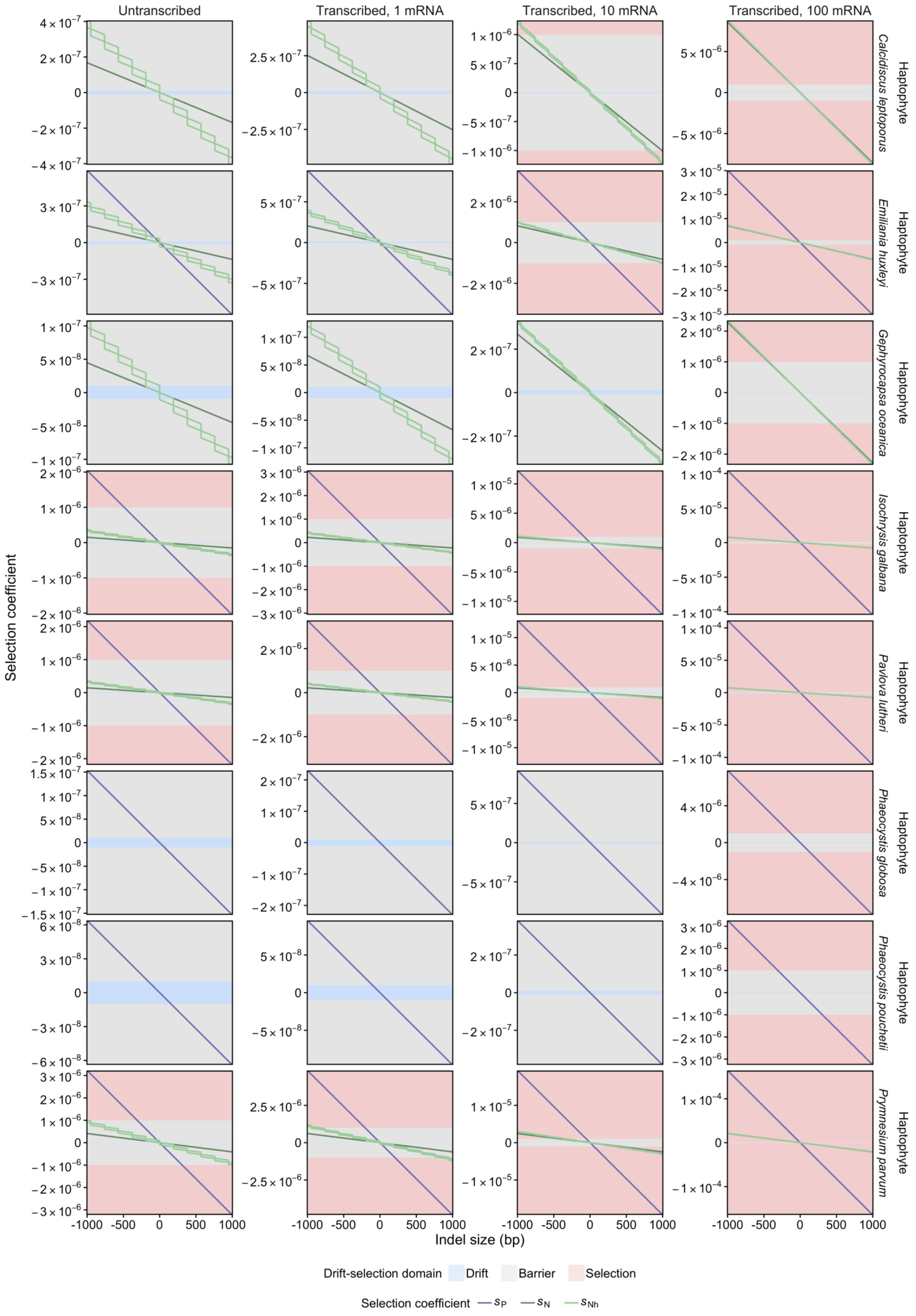

Untranscribed
Transcribed, 1 mRNA
Transcribed, 10 mRNA
Transcribed, 100 mRNA
Haptophyte
Calcidiscus leptoporus
Emiliania huxleyi
Gephyrocapsa oceanica
Isochrysis galbana
Pavlova lutheri
Phaeocystis globosa
Phaeocystis pouchetii
Prymnesium parvum
Selection coefficient
Indel size (bp)
Drift-selection domain
Drift
Barrier
Selection
Selection coefficient
s_P
s_N
s_{Nh}

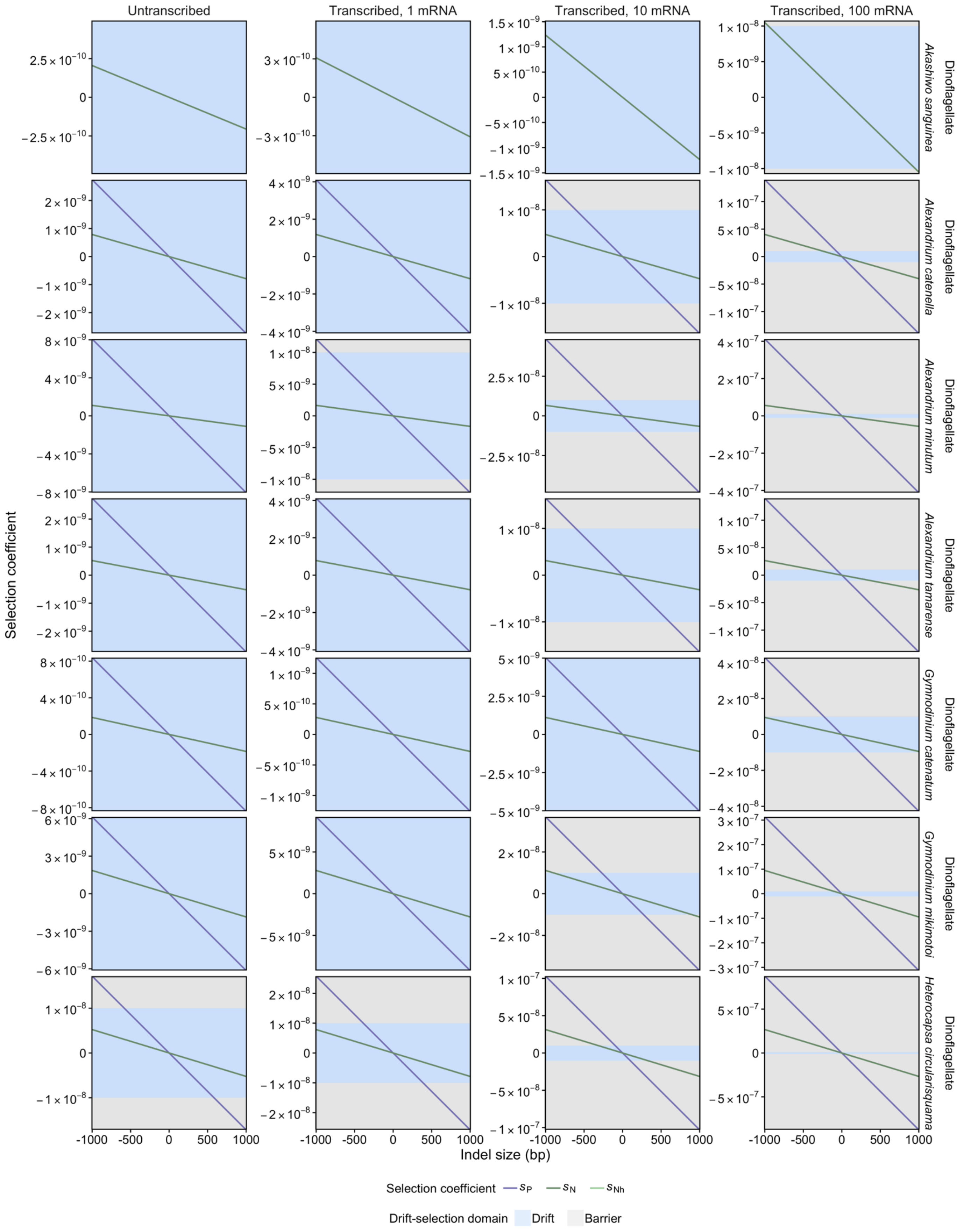

Untranscribed
Transcribed, 1 mRNA
Transcribed, 10 mRNA
Transcribed, 100 mRNA
Dinoflagellate
Akashiwo sanguinea
Dinoflagellate
Alexandrium catenella
Dinoflagellate
Alexandrium minutum
Dinoflagellate
Alexandrium tamarense
Dinoflagellate
Gymnodinium catenatum
Dinoflagellate
Gymnodinium mikimotoi
Dinoflagellate
Heterocapsa circularisquama
Selection coefficient
Indel size (bp)
-1000 -500 0 500 1000
Selection coefficient s_P s_N s_{Nh}
Drift-selection domain Drift Barrier

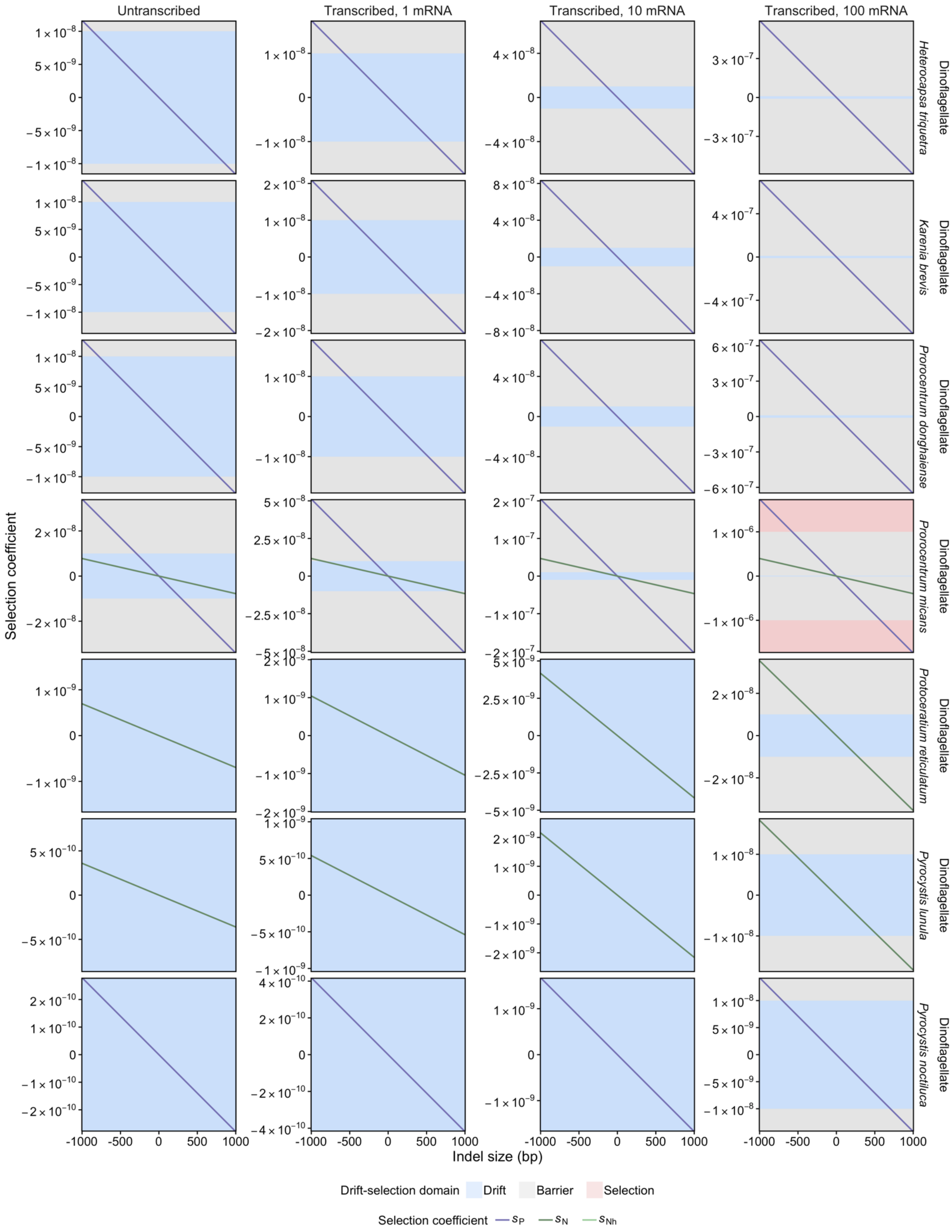

Untranscribed
Transcribed, 1 mRNA
Transcribed, 10 mRNA
Transcribed, 100 mRNA
Dinoflagellate *Heterocapsa triquetra*
Dinoflagellate *Karenia brevis*
Dinoflagellate *Prorocentrum donghaiense*
Dinoflagellate *Prorocentrum micans*
Dinoflagellate *Protoceratium reticulatum*
Dinoflagellate *Pyrocystis lunula*
Dinoflagellate *Pyrocystis noctiluca*
Selection coefficient
Indel size (bp)
-1000 -500 0 500 1000
Drift-selection domain Drift Barrier Selection
Selection coefficient s_P s_N s_{Nh}

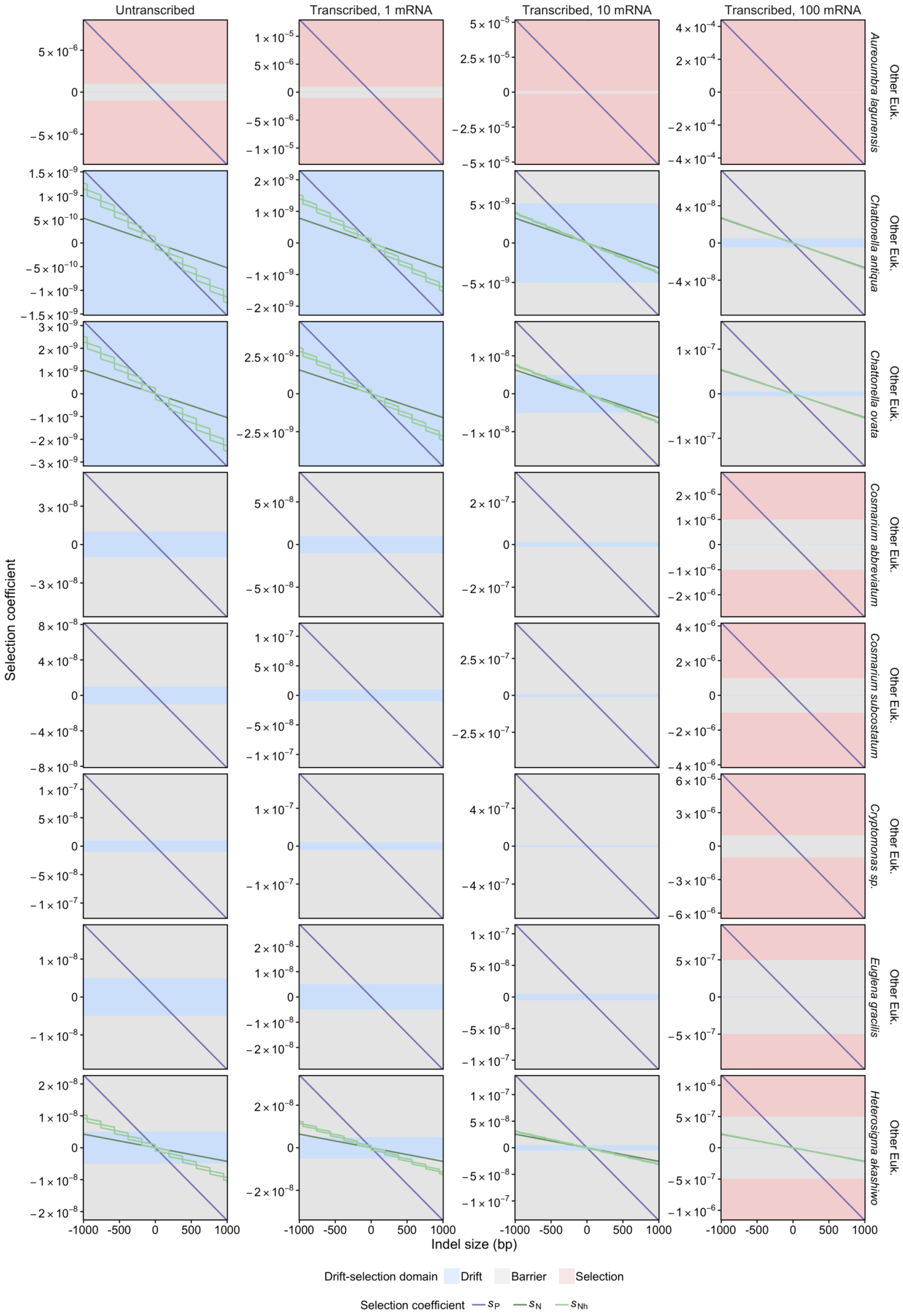

Untranscribed
Transcribed, 1 mRNA
Transcribed, 10 mRNA
Transcribed, 100 mRNA
Other Euk.
Aureoumbra lagunensis
Chattonella antiqua
Chattonella ovata
Cosmarium abbreviatum
Cosmarium subcostatum
Cryptomonas sp.
Euglena gracilis
Heterosigma akashiwo
Selection coefficient
Indel size (bp)
Drift-selection domain
Drift
Barrier
Selection
Selection coefficient
s_P
s_N
s_{Nh}

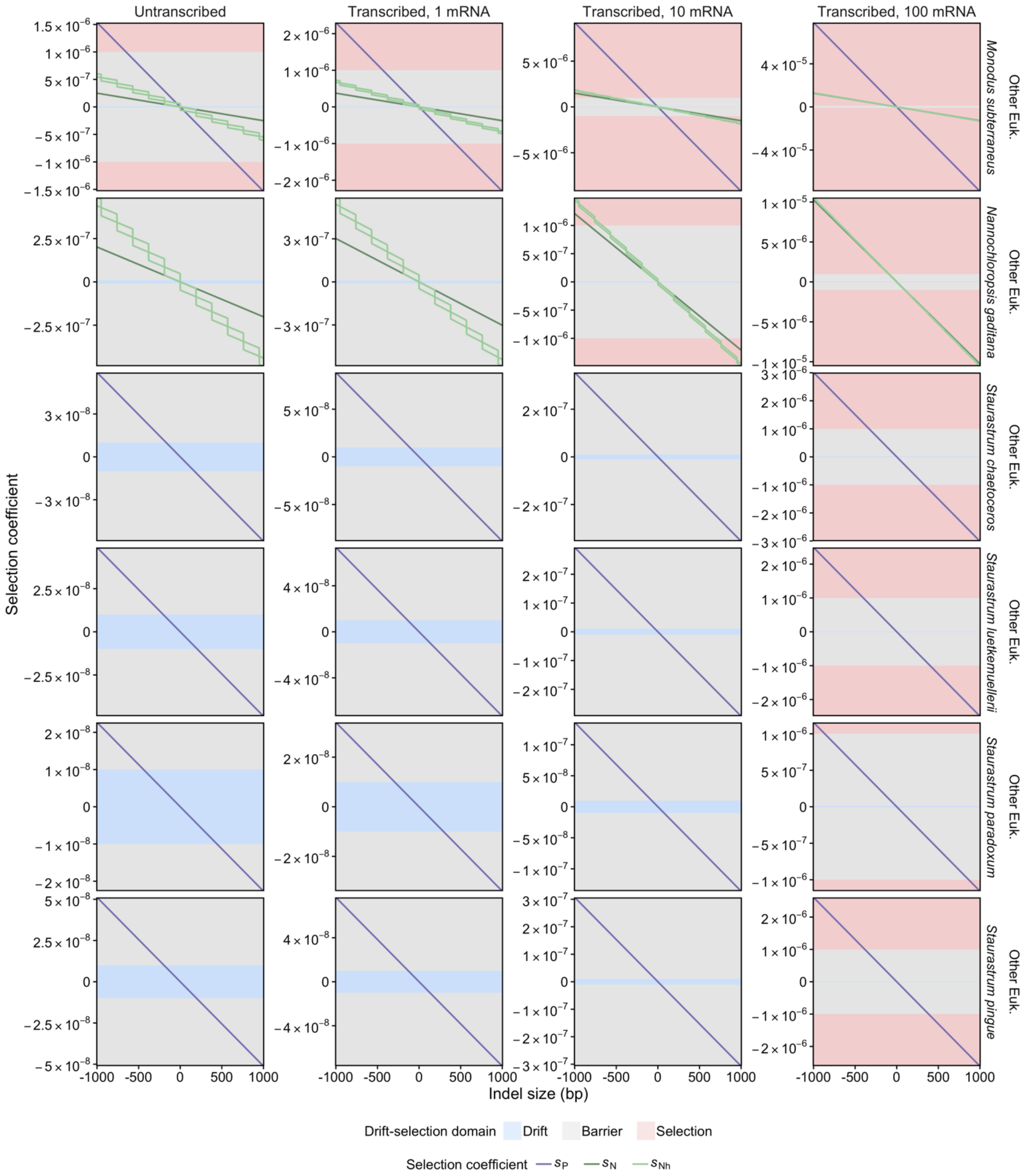

Fig. S3. Relationships between the selection coefficient (*s*) and indel size in untranscribed and transcribed (1, 10 and 100 mRNA copies) regions for each species. Selection coefficients were calculated based on the impact of indels on P (s_P) or N (s_{Nh}: accounting for histone N cost and s_N: excluding histone-associated N costs) cellular requirements under P and N limitation conditions, respectively (see legend). When N in histones is accounted for, two lines are shown because a given indel can result in two different s_{Nh} values depending on whether a new nucleosome is required: the upper line includes one additional nucleosome for insertions and one fewer nucleosome for deletions. Background color indicates the relative importance of drift (blue) versus natural selection (pink) on the probability of indel fixation. Panels are grouped by taxonomic group and ordered by species name. Note that different Y-axis scales are used for each panel

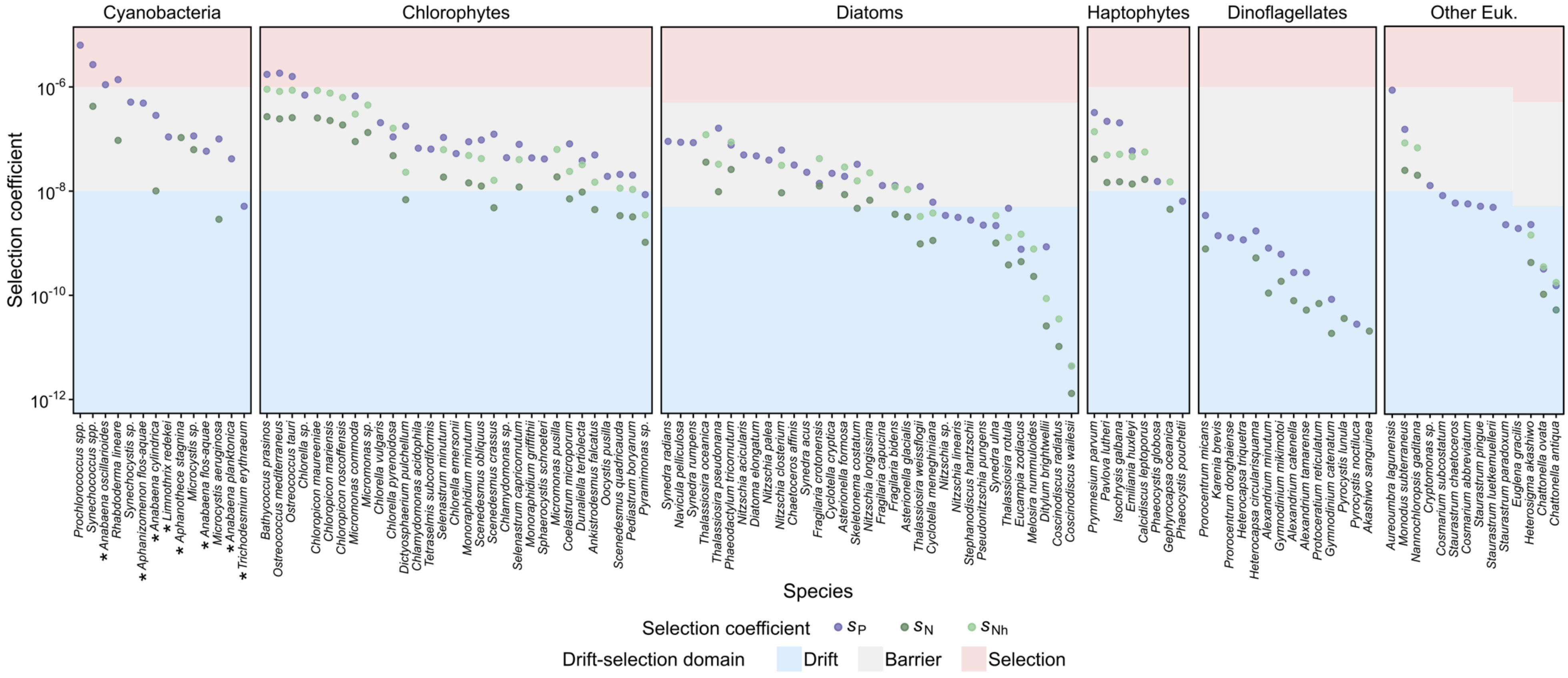

Fig. S4. Selection coefficients associated with a 100 bp deletion in an untranscribed region. Selection coefficients were calculated based on the impact of indels on P (s_P) or N (s_{Nh}: accounting for histone N cost and s_N: excluding histone-associated N costs) cellular requirements under P and N limitation conditions, respectively (see legend). Background color indicates the relative importance of drift (blue) versus natural selection (pink) on the indel fixation probability. * Indicates diazotrophic species.

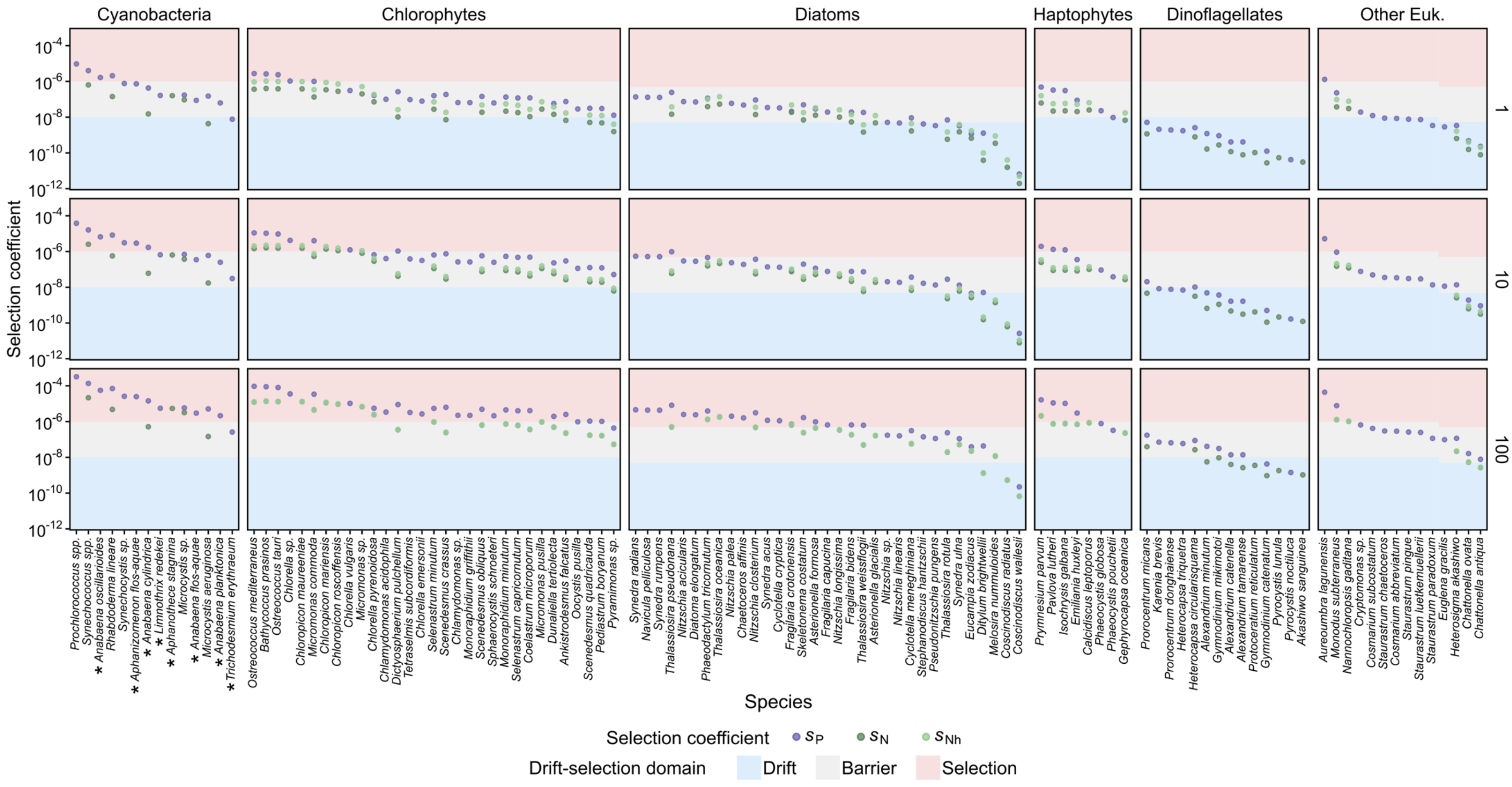

Fig. S5. Selection coefficients associated with the impact of a 100 bp deletion within a transcribed region with 1, 10 or 100 mRNA copies. Selection coefficients were calculated based on the impact of indels on P (s_P) or N (s_{Nh}: accounting for histone N cost and s_N: excluding histone-associated N costs) cellular requirements under P and N limitation conditions, respectively (see legend). Background color indicates the relative importance of drift (blue) versus natural selection (pink) on the indel fixation probability. * Indicates diazotrophic species.

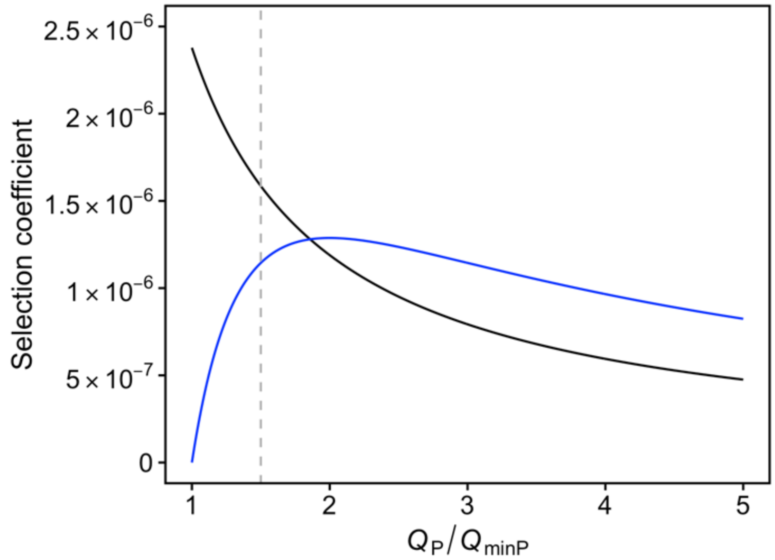

Fig. S6. Selection coefficients for the effect of a 100 bp deletion on phosphorus cellular requirements as a function of phosphorus quota (Q_P) in *Ostreococcus tauri*. Black line represents dimensionless selection coefficient (s). Blue line represents selection coefficient per unit of time (s_{time}). When estimating s_{time} the maximum growth rate (Table S1) was used instead of the growth rate at infinite Q_P, which was not available. The Q_P in x-axis is expressed relative to Q_{minP}. The vertical dashed line indicates $Q_P = 1.5 \times Q_{minP}$, which was used to estimate s values throughout the manuscript. The relationships between the selection coefficients and Q are illustrated for a single species and Q_P only, as their shapes are consistent across species and under nitrogen-limiting conditions.

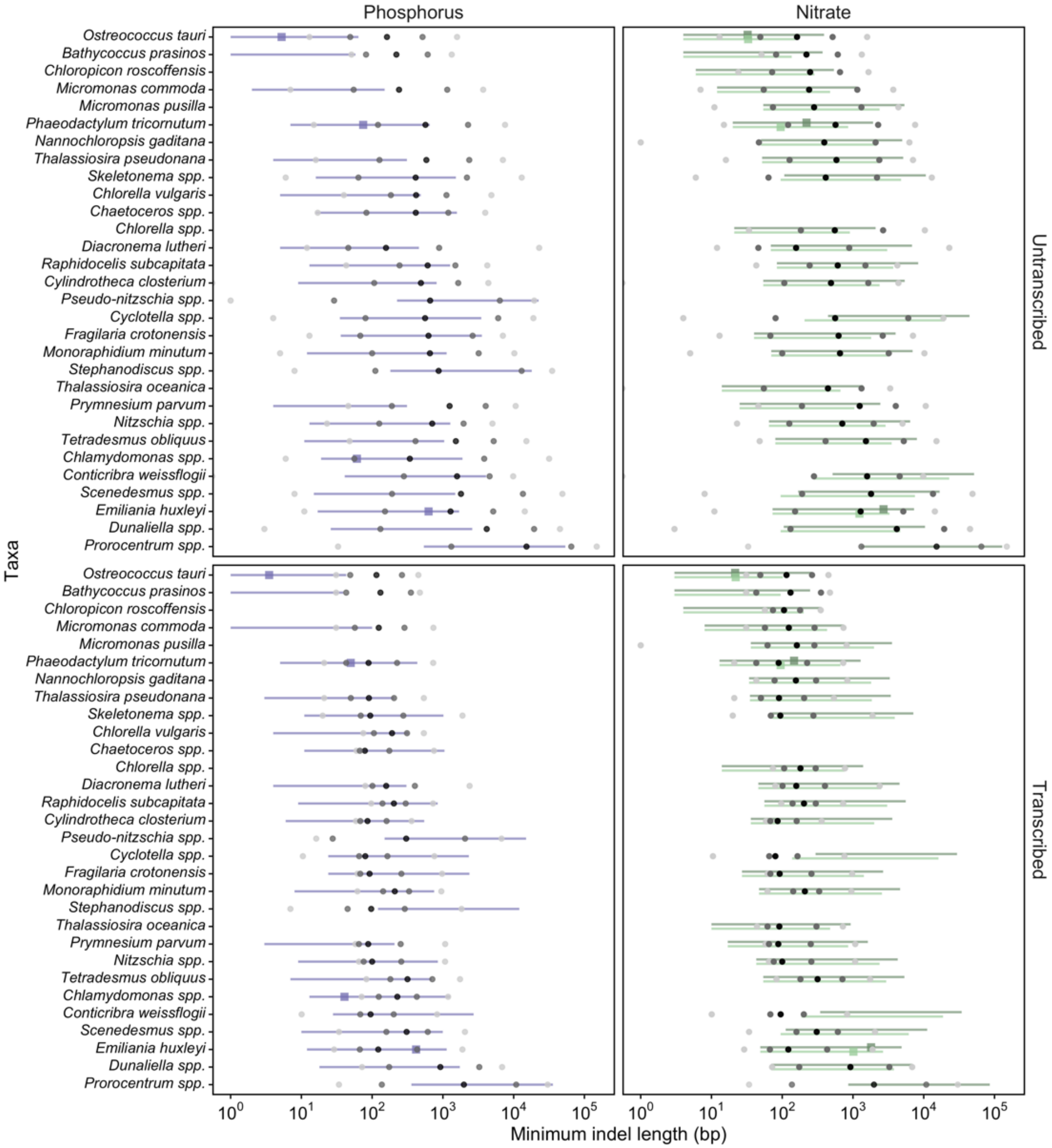

Fig. S7. Minimum length ranges of indels (x-axis) under scenarios of P (purple) and N (green) limitation and size on non-coding regions for eukaryotic taxa. Taxa are ordered based on their genome size. Minimum and maximum values for the minimum length of indels required for selection to be effective ($|s| = 1/N_e$ in haploids or $|s| = 1/2N_e$ diploids) were defined for $N_e = 10^8$ and $N_e = 10^6$, respectively. When available, squares represent minimum lengths estimated from N_e values. Circles represent the size of median (black), 10th and 90th (dark grey), and 1st and 99th (light grey) percentiles of intergenic (upper panels) and intronic regions (bottom panels). Top panels: untranscribed regions. Lower panels: transcribed regions assuming one copy of mRNA. N limitation: Dark and light green colors represent the minimum length ranges excluding histone-associated N costs and accounting for histone N cost. When histone-associated nitrogen costs were included, lower and upper limits were calculated by assuming that nucleosome number was unaffected by indels shorter than 95 bp, and that one additional nucleosome was removed or added for every 190 bp deleted or inserted, respectively.

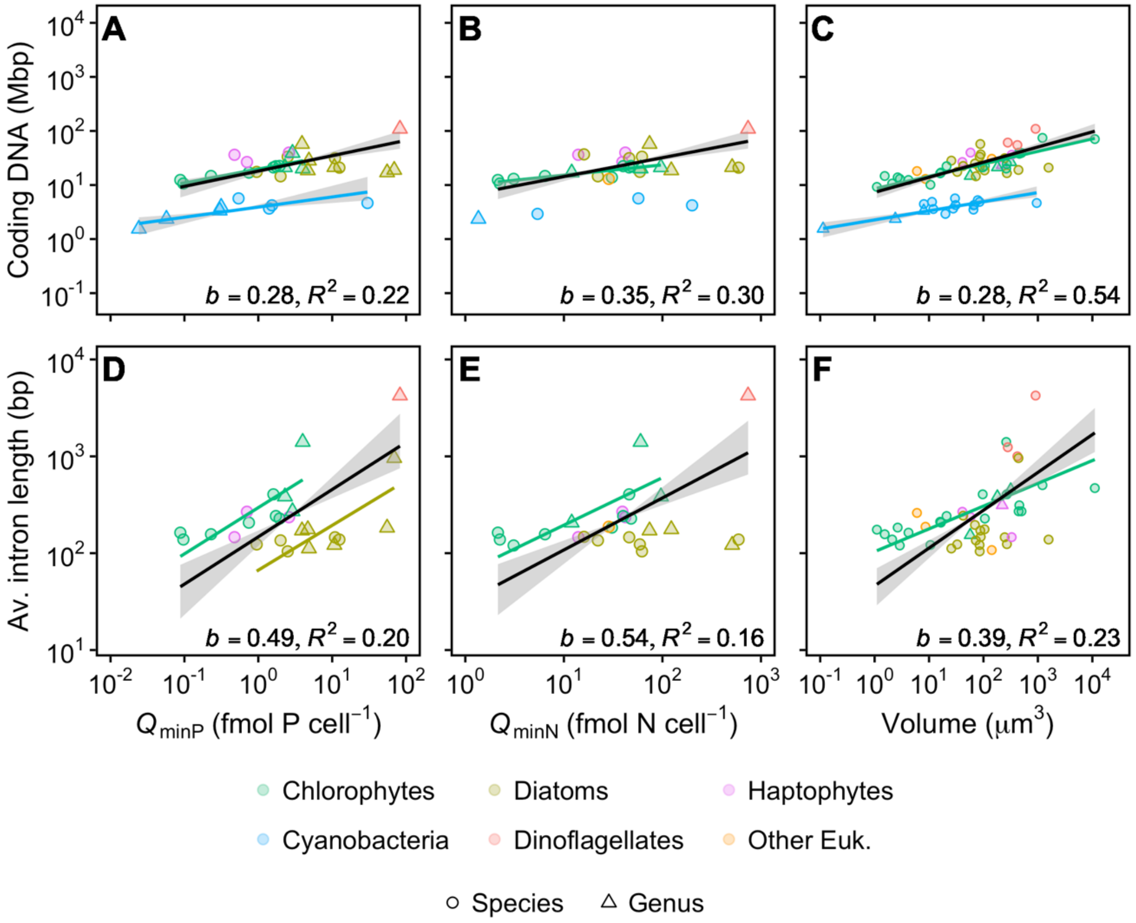

Fig. S8. Log-log relationships between genomic traits and Q_{minP}, Q_{minN} and cell volume. (A, B, C) Total coding DNA. **(D, E, F)** Average intron size. Each point corresponds to a different species or genus. Point color and shape indicate taxonomic affiliation and level, respectively. Note that the set of species and the x-axis scales differ for Q_{minP}, Q_{minN}, and cell volume relationships. Black lines show regression fits when all eukaryotic species were pooled together. The corresponding slope coefficients (b) and explained variances (R^2) are shown at the bottom of each panel. The 95% confidence interval for the regression for cyanobacteria and eukaryotes is shown in grey. Group-specific regression fits are included when slopes differed significantly from 0 and the sample size exceeded four. There is no average intron size data for cyanobacteria.

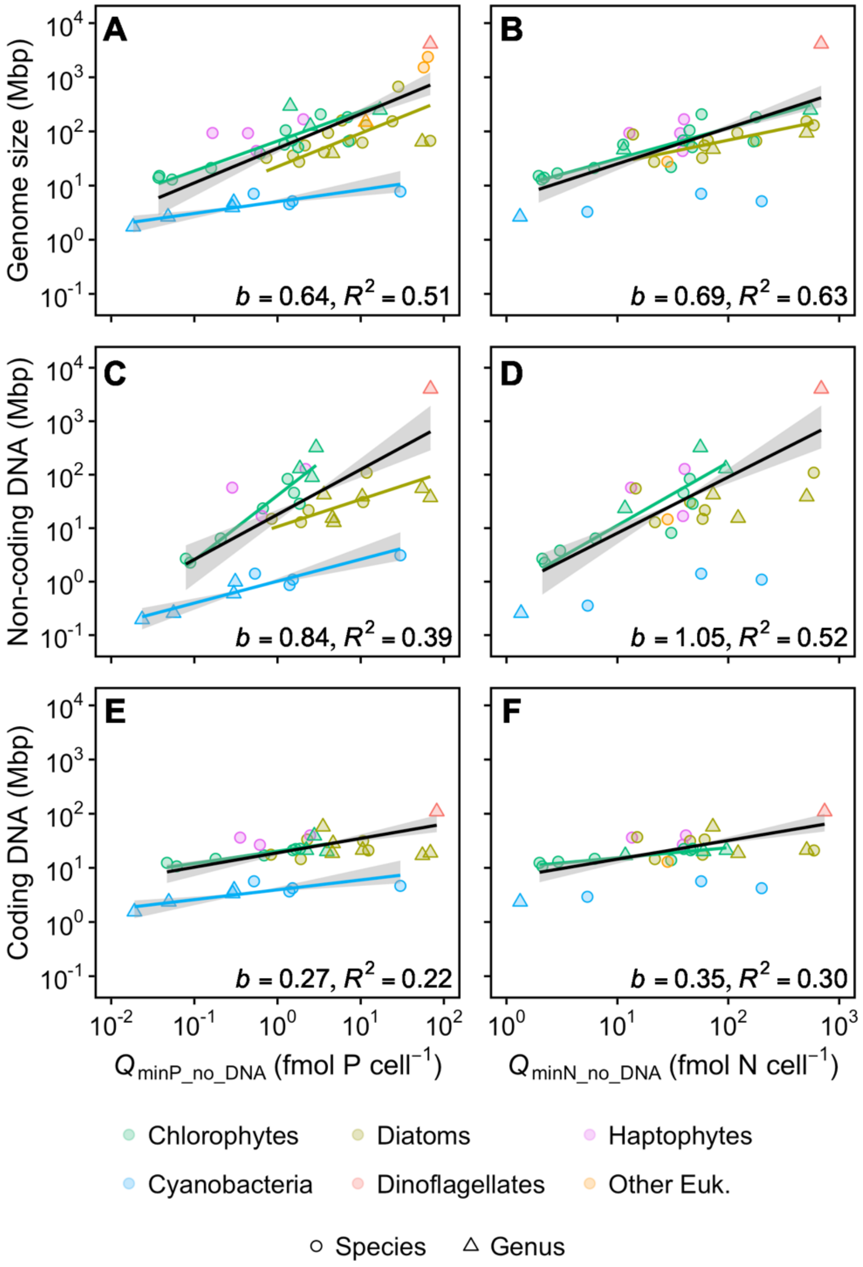

Fig. S9. Log-log relationships between genomic traits and corrected Q_{minP} and Q_{minN}, excluding the N and P content of the corresponding DNA compartment. (A, B) Genome size. **(C, D)** Total non-coding DNA. **(E, F)** Total coding DNA. Point color and shape indicate taxonomic affiliation and level, respectively. Note that the set of species and the x-axis scales differ for Q_{minP} and Q_{minN}. Black lines show regression fits when all eukaryotic species were pooled together. The corresponding slope coefficients (b) and explained variances (R^2) are shown at the bottom of each panel. The 95% confidence interval for the regression for cyanobacteria and eukaryotes is shown in grey. Group-specific regression fits are included when slopes differed significantly from 0 and sample size exceeded four. There is no average intron size data for cyanobacteria.

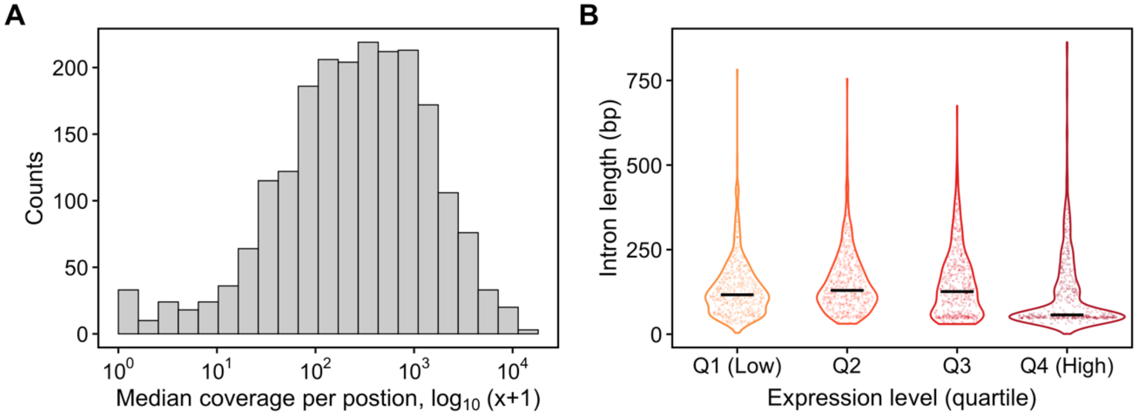

Fig. S10. Transcription rate of introns in *Ostreococcus tauri*. (A) Distribution of median RNA coverage per position of introns. **(B)** Length of introns grouped into quartiles based on their median RNA coverage per position, from lowest Q1 to highest Q4. Black lines show median values. Pairwise Wilcoxon tests showed all comparisons significant but Q1 vs Q3 and Q2 vs Q3 (Table S8).

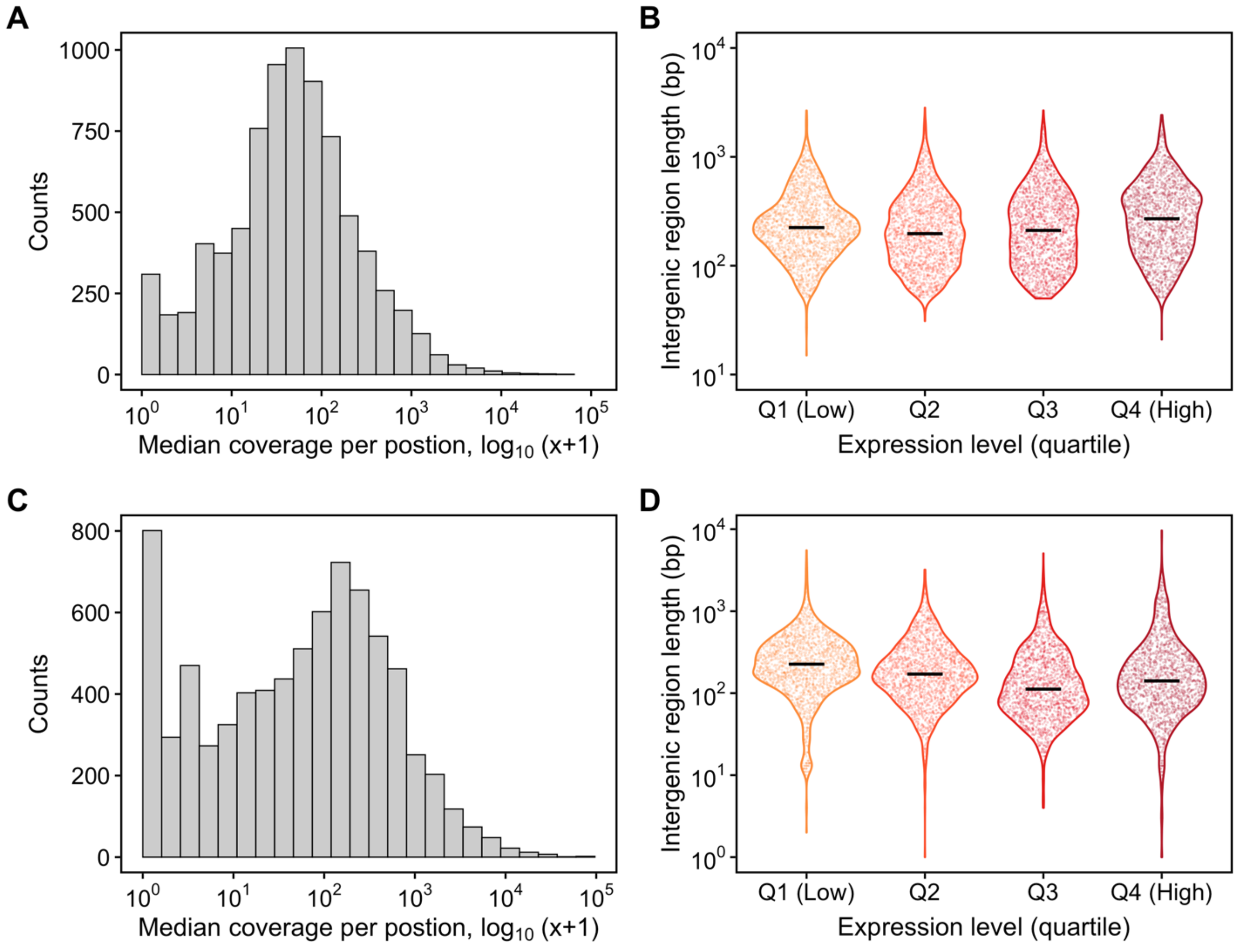

Fig. S11. Transcription rate of intergenic regions in *Bathycoccus prasinos* and *Ostreococcus tauri.* (A) Distribution of median RNA coverage per position of intergenic regions in *B. prasinos*. **(B)** Length of intergenic regions in *B. prasinos* grouped into quartiles based on their median RNA coverage per position, from lowest (Q1) to highest (Q4). Pairwise Wilcoxon tests showed all comparisons significant (Table S8). **(C)** Distribution of median coverage per position of intergenic regions in *O. tauri*. **(D)** Length of intergenic regions in *O. tauri* grouped into quartiles based on their median RNA coverage per position. Pairwise Wilcoxon tests showed all comparisons significant (Table S8). Horizontal black lines show median values.

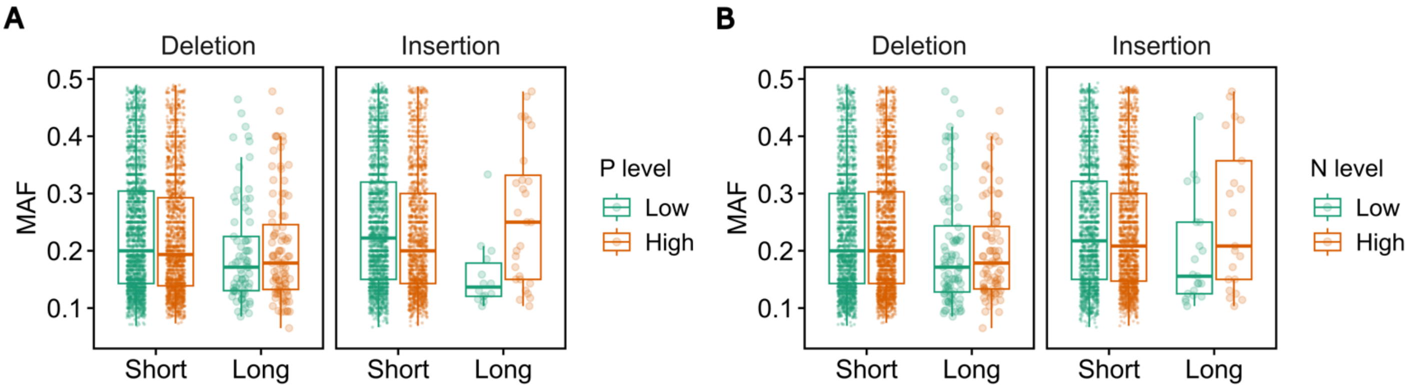

Fig. S12. Minor allele frequency (MAF) of deletions and insertions as a function of environmental nutrient levels for the six target species of Mamiellales. (**A**) MAF of short (<10bp) and long (≥ 10bp) indels in low (green) and high (orange) phosphate environments. (**B**) MAF of short (<10bp) and long (≥ 10bp) indels in low (green) and high (orange) nitrate and nitrite environments. Results of the pairwise comparisons corresponding to panels A and B are provided in Table S9.

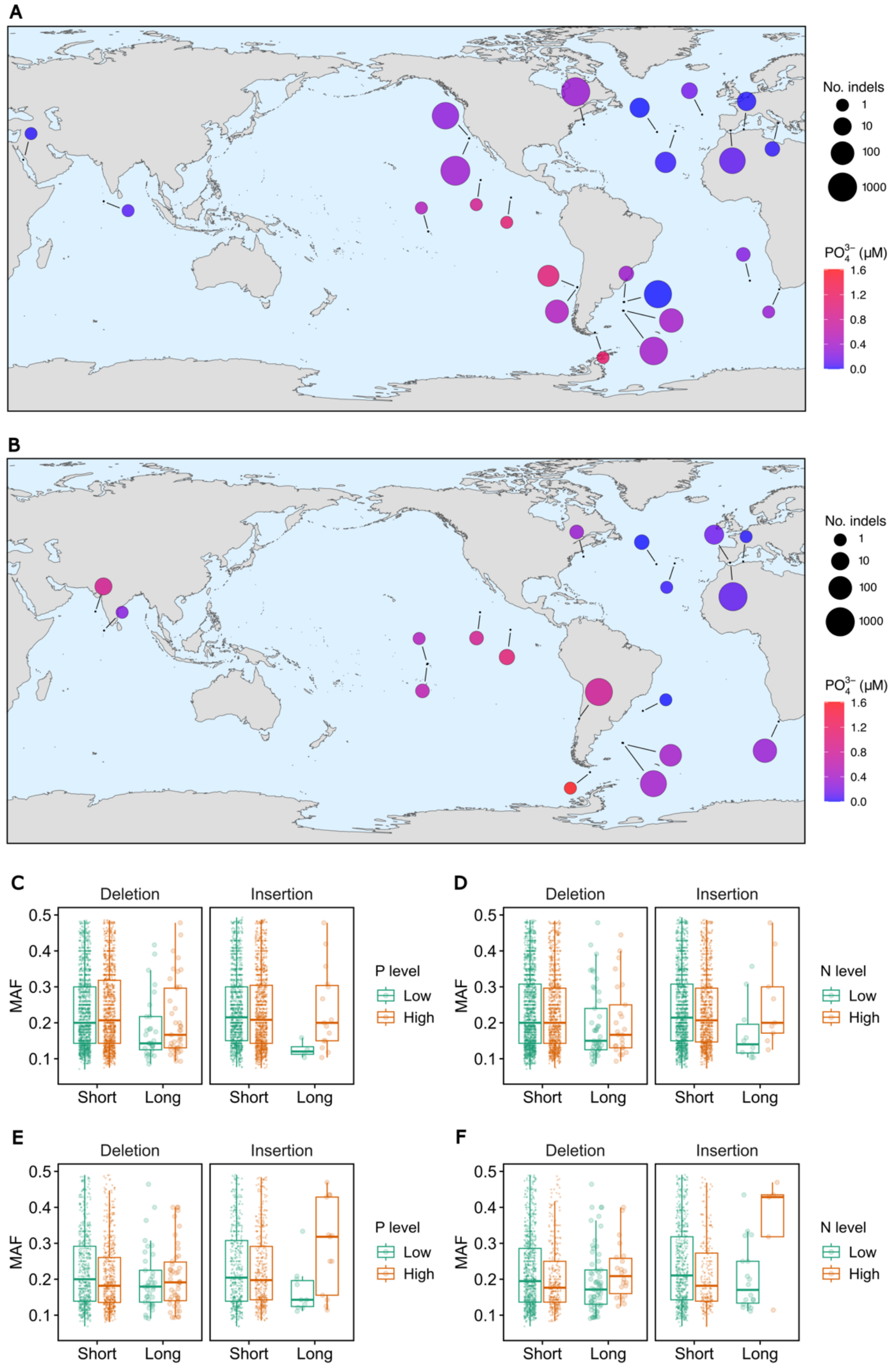

Fig. S13. Worldwide distribution of metagenomes with indel polymorphisms for *Bathycoccus prasinos* and *Ostreococcus lucimarinus* and minor allele frequency (MAF) of indels in low and high nutrient environments. (**A, B**) Worldwide distribution of metagenomes with indel polymorphism for *B. prasinos* (A) and *O. lucimarinus* (B). (**C**) MAF of short (<10bp) and long (≥ 10bp) indels in *B. prasinos* in low (green) and high (orange) phosphate environments. (**D**) MAF of short (<10bp) and long (≥ 10bp) indels in *B. prasinos* in low (green) and high (orange) nitrate and nitrite environments. (**E**) MAF of short (<10bp) and long (≥ 10bp) indels in *O. lucimarinus* in low and high phosphate environments. (**F**) MAF of short (<10bp) and long (≥ 10bp) indels in *O. lucimarinus* in low and high nitrate and nitrite environments. Results of the pairwise comparisons corresponding to panels C-F are provided in Table S9.

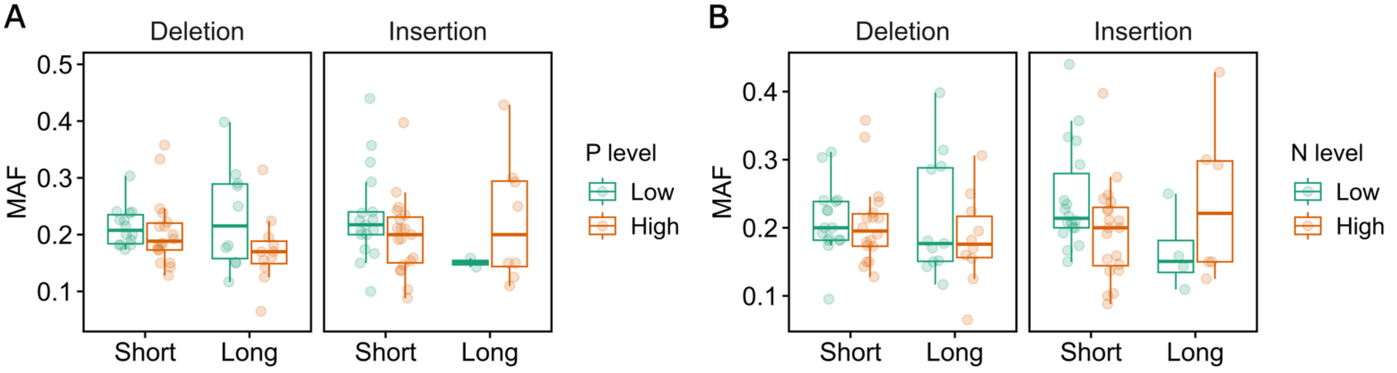

Fig. S14. Median minor allele frequency (MAF) of insertions and deletions per metagenome in the Mamiellales fraction as a function of nutrient levels. (**A**) Median MAF of short (<10bp) and long (≥ 10bp) indels in the six target species of Mamiellales in low (green) and high (orange) phosphate environments. (**B**) Median MAF of short (<10bp) and long (≥ 10bp) indels in Mamiellales in low (green) and high (orange) nitrate and nitrite environments.

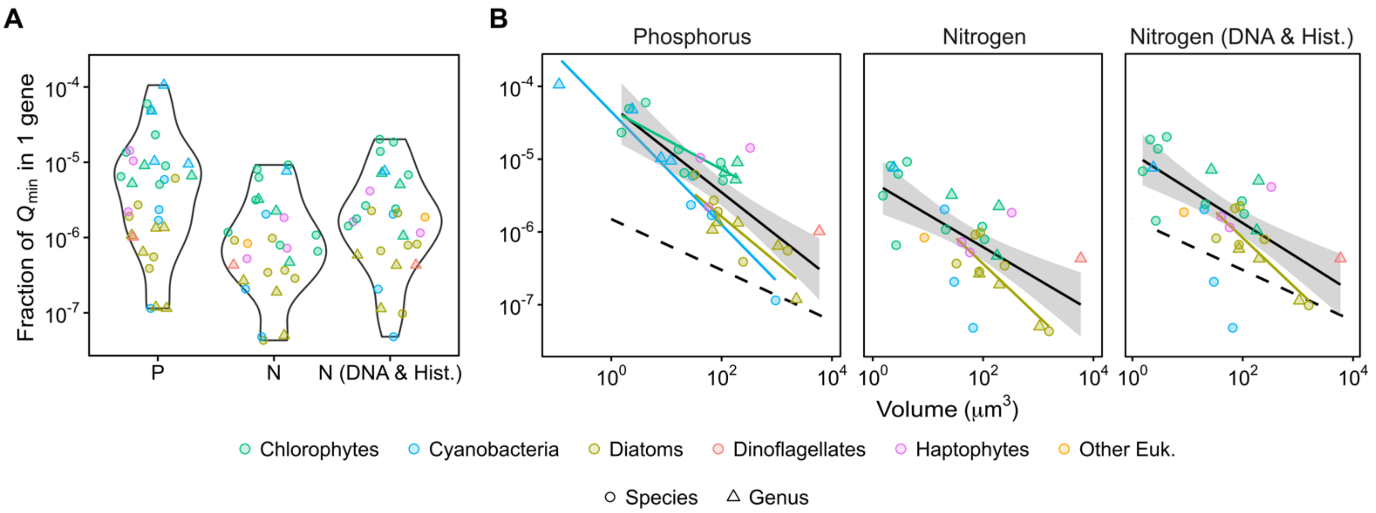

Fig. S15. Nutrient cost of a gene-sized DNA sequence expressed as a fraction of the minimum quota ($Q_{\mathbf{min}}$) for phosphorus (P) or nitrogen (N). (A) Nutrient cost of a DNA sequence with the same length as an average-sized gene, with average GC content of the genome, relative to Q_{minP} and Q_{minN} ($\Delta Q_{\mathrm{min}}/Q_{\mathrm{min}}$) across species and genera. **(B)** Relationship between the fractional nutrient cost of a gene-sized DNA sequence (phosphorus or nitrogen) and cell volume. For nitrogen, fractional cost was estimated either by considering only the nitrogen in nucleotides or by including nitrogen in both nucleotides and histone proteins, assuming chromatosomes occur every 190 bp. Point color indicates taxonomic affiliation. Black solid lines show regression fits when all eukaryotic species were pooled. The 95% confidence interval is shown in grey. Black dashed lines show the regression fit for eukaryotes based on the fractional energetic cost of gene replication —estimated considering histone costs— reported in Lynch and Marinov (*7*). Note that the fractional cost is identical for transcribed and untranscribed regions of similar size, since only the phosphorus and nitrogen in DNA nucleotides (and in histones for nitrogen) are considered.

Table S1. Maximum growth rate (μ_{max}) and minimum quota (Q_{min}) for P and N estimated in five species for this study.

Nutrient: limiting nutrient in the culture medium for which traits were measured. μ_{max} Rep.: maximum growth rate in each replicate, estimated as the slope coefficient (± standard error) of the regression between the natural logarithm of abundances and time during the exponential growth phase. R^2 and p-values from the regression analysis to estimate μ_{max} Rep. are provided in separate columns. Q_{min} Rep.: Q_{min} measured for each replicate. μ_{max} and Q_{min}: average values ± standard error across the three replicates.

Species	Nutrient	Replicate	μ_{max} Rep. (d^{-1})	R^2	p-value	Q_{min} Rep. (fmol $cell^{-1}$)	μ_{max} (d^{-1})	Q_{min} (fmol $cell^{-1}$)
O. tauri	Phosphorus	R1	1.55 ± 0.07	0.996	0.002	0.092		
O. tauri	Phosphorus	R2	1.50 ± 0.08	0.994	0.003	0.095	1.50 ± 0.03	0.097 ± 0.003
O. tauri	Phosphorus	R3	1.46 ± 0.10	0.991	0.004	0.103		
O. tauri	Nitrogen	R1	1.45 ± 0.12	0.986	0.007	2.361		
O. tauri	Nitrogen	R2	1.44 ± 0.11	0.988	0.006	2.215	1.44 ± 0.01	2.243 ± 0.062
O. tauri	Nitrogen	R3	1.43 ± 0.12	0.985	0.007	2.153		
O. mediterraneus	Phosphorus	R1	1.26 ± 0.07	0.991	<0.001	0.080		
O. mediterraneus	Phosphorus	R2	1.28 ± 0.06	0.993	<0.001	0.088	1.27 ± 0.01	0.084 ± 0.002
O. mediterraneus	Phosphorus	R3	1.25 ± 0.07	0.991	<0.001	0.083		
O. mediterraneus	Nitrogen	R1	1.36 ± 0.07	0.992	<0.001	2.432		
O. mediterraneus	Nitrogen	R2	1.34 ± 0.07	0.991	<0.001	2.293	1.32 ± 0.03	2.362 ± 0.040
O. mediterraneus	Nitrogen	R3	1.25 ± 0.10	0.982	0.001	2.362		
B. prasinos	Phosphorus	R1	1.12 ± 0.06	0.992	<0.001	0.091		
B. prasinos	Phosphorus	R2	1.10 ± 0.04	0.995	<0.001	0.085	1.11 ± 0.01	0.088 ± 0.002
B. prasinos	Phosphorus	R3	1.10 ± 0.05	0.995	<0.001	0.088		
B. prasinos	Nitrogen	R1	1.24 ± 0.03	0.998	<0.001	2.243		
B. prasinos	Nitrogen	R2	1.21 ± 0.04	0.997	<0.001	2.063	1.21 ± 0.02	2.141 ± 0.053
B. prasinos	Nitrogen	R3	1.19 ± 0.04	0.997	<0.001	2.117		
M. commoda	Phosphorus	R1	0.66 ± 0.01	0.999	<0.001	0.219		
M. commoda	Phosphorus	R2	0.61 ± 0.01	0.998	<0.001	0.237	0.63 ± 0.01	0.229 ± 0.005
M. commoda	Phosphorus	R3	0.62 ± 0.01	0.998	<0.001	0.230		
M. commoda	Nitrogen	R1	0.63 ± 0.01	1.000	<0.001	6.375		
M. commoda	Nitrogen	R2	0.63 ± 0.01	1.000	<0.001	6.647	0.64 ± 0.00	6.429 ± 0.114
M. commoda	Nitrogen	R3	0.64 ± 0.01	0.999	<0.001	6.264		
Pyramimonas sp.	Phosphorus	R1	0.98 ± 0.04	0.995	<0.001	17.475		
Pyramimonas sp.	Phosphorus	R2	0.91 ± 0.05	0.992	<0.001	18.927	0.94 ± 0.02	17.880 ± 0.528
Pyramimonas sp.	Phosphorus	R3	0.93 ± 0.04	0.994	<0.001	17.238		
Pyramimonas sp.	Nitrogen	R1	0.82 ± 0.04	0.991	<0.001	572.539		
Pyramimonas sp.	Nitrogen	R2	0.81 ± 0.03	0.993	<0.001	547.869	0.82 ± 0.00	552.664 ± 10.372
Pyramimonas sp.	Nitrogen	R3	0.82 ± 0.03	0.995	<0.001	537.585		

Table S2. **Available effective population (N_e) size estimates for different phytoplankton species.**

Species	N_e	Reference
Prochlorococcus marinus	$1.7\ 10^7$	(*105*)
Ostreococcus tauri	$1.2\ 10^7$	(*106*)
Phaeodactylum tricornutum	$8.7\ 10^6$	(*107*)
Chlamydomonas reinhardtii	$3.1\ 10^7$	(*108*)
Emiliania huxleyi	$2.7\ 10^6$	(*109*)

Table S3. Coefficients and summary statistics for the SMA regression analysis between genomic traits and genome length.

Regressions were fitted only when the number of observations (n) was greater than 4. The 95% confidence intervals for intercepts and slopes are shown in brackets. Likelihood ratio test (LR) and corresponding p-values (p_{LR}) are provided for comparisons of slopes between prokaryotes and eukaryotes, and between chlorophytes and diatoms.

Variable	Taxon	Intercept	Slope	R^2	n	p-value	LR	p_{LR}
Av. intergenic length	Cyanobacteria	1.59 (1.41 , 1.77)	1.03 (0.80 , 1.32)	0.51	34	<0.001		
	Eukaryotes	1.61 (1.43 , 1.80)	0.85 (0.76 , 0.95)	0.83	59	<0.001	1.795	0.180
	Chlorophytes	1.42 (1.18 , 1.67)	0.94 (0.84 , 1.11)	0.87	30	<0.001	0.095	0.757
	Diatoms	1.29 (0.45 , 2.14)	1.04 (0.66 , 1.62)	0.18	19	0.068		
Av. intron length	Eukaryotes	1.26 (1.06 , 1.45)	0.61 (0.52 , 0.72)	0.62	59	<0.001		
	Chlorophytes	1.54 (1.37 , 1.71)	0.50 (0.42 , 0.60)	0.79	30	<0.001	5.553	0.018
	Diatoms	0.58 (-0.25 , 1.41)	0.93 (0.58 , 1.52)	0.03	19	0.49		
gene number	Cyanobacteria	3.11 (3.04 , 3.17)	0.79 (0.70 , 0.89)	0.89	34	<0.001		
	Eukaryotes	3.39 (3.27 , 3.50)	0.43 (0.38 , 0.50)	0.72	59	<0.001	35.56	<0.001
	Chlorophytes	3.42 (3.31 , 3.53)	0.39 (0.33 , 0.46)	0.83	30	<0.001	9.673	0.002
	Diatoms	2.82 (2.24 , 3.40)	0.79 (0.53 , 1.19)	0.34	19	0.009		
Av. introns per gene	Eukaryotes	-8.75 (-11.31 , -6.19)	6.56 (5.37 , 8.01)	0.43	59	<0.001		
	Chlorophytes	-6.80 (-9.83 , -3.77)	6.33 (4.92 , 8.16)	0.56	30	<0.001	17.35	<0.001
	Diatoms	-2.31 (-3.99 , -0.63)	1.88 (1.16 , 3.06)	0.03	10	0.504		

Table S4. Coefficients and summary statistics for the SMA regression analysis between the size of different DNA regions and Q_{min} for phosphorus (P) and nitrogen (N).
Regressions were fitted only when the number of observations (n) was greater than 4. The 95% confidence intervals for slopes are shown in brackets. p-slope$_0$ and p-slope$_1$ indicate p-values for testing whether slope coefficients are equal to 0 and 1, respectively. Likelihood ratio test (LR) and corresponding p-values (p_{LR}) are provided for comparing slopes between prokaryotes and eukaryotes, and between chlorophytes and diatoms.

DNA region	Nutrient	Taxon	R^2	n	Intercept	Slope	p-slope$_0$	p-slope$_1$	LR	p_{LR}
Genome	Phosphorus	Cyanobacteria	0.73	8	0.70	0.23 (0.14 , 0.37)	0.007	<0.001		
		Eukaryotes	0.56	38	1.59	0.70 (0.56 , 0.87)	<0.001	0.002	11.184	0.001
		Chlorophytes	0.80	15	1.74	0.63 (0.48 , 0.82)	<0.001	0.002	0.005	0.942
		Diatoms	0.35	15	1.30	0.64 (0.40 , 1.02)	0.020	0.059		
	Nitrogen	Eukaryotes	0.64	35	0.67	0.70 (0.57 , 0.86)	<0.001	0.001		
		Chlorophytes	0.79	15	0.90	0.58 (0.44 , 0.76)	<0.001	0.001	0.003	0.959
		Diatoms	0.61	13	0.69	0.58 (0.39 , 0.87)	0.002	0.012		
Non-coding	Phosphorus	Cyanobacteria	0.87	8	0.01	0.41 (0.29 , 0.59)	0.001	<0.001		
		Eukaryotes	0.43	25	1.21	0.86 (0.62 , 1.18)	<0.001	0.336	8.459	0.004
		Chlorophytes	0.93	10	1.54	1.18 (0.95 , 1.47)	<0.001	0.120	7.028	0.008
		Diatoms	0.41	11	1.01	0.52 (0.30 , 0.90)	0.033	0.022		
	Nitrogen	Eukaryotes	0.54	25	-0.15	1.05 (0.78 , 1.40)	<0.001	0.746		
		Chlorophytes	0.80	11	-0.13	1.17 (0.85 , 1.63)	<0.001	0.301	3.328	0.068
		Diatoms	0.20	9	0.39	0.57 (0.27 , 1.18)	0.227	0.120		
Coding	Phosphorus	Cyanobacteria	0.57	8	0.59	0.19 (0.10 , 0.35)	0.030	<0.001		
		Eukaryotes	0.22	25	1.26	0.28 (0.20 , 0.41)	0.019	<0.001	1.477	0.224
		Chlorophytes	0.73	10	1.29	0.26 (0.17 , 0.39)	0.002	<0.001	0.155	0.694
		Diatoms	0.02	11	1.63	-0.30 (-0.60 , -0.15)	0.658	0.001		
	Nitrogen	Eukaryotes	0.30	25	0.81	0.35 (0.25 , 0.50)	0.004	<0.001		
		Chlorophytes	0.79	11	0.99	0.19 (0.14 , 0.27)	<0.001	<0.001	2.111	0.146
		Diatoms	0.04	9	2.08	-0.35 (-0.78 , -0.16)	0.613	0.012		
Intergenic	Phosphorus	Cyanobacteria	0.87	8	0.00	0.41 (0.29 , 0.58)	0.001	<0.001		
		Eukaryotes	0.50	25	1.06	0.82 (0.61 , 1.11)	<0.001	0.184	7.955	0.005
		Chlorophytes	0.82	10	1.26	1.05 (0.74 , 1.48)	<0.001	0.763	3.968	0.046
		Diatoms	0.37	11	0.92	0.55 (0.31 , 0.96)	0.047	0.038		
	Nitrogen	Eukaryotes	0.56	25	-0.26	1.01 (0.76 , 1.34)	<0.001	0.928		
		Chlorophytes	0.76	11	-0.20	1.04 (0.72 , 1.50)	0.001	0.820	1.860	0.173
		Diatoms	0.19	9	0.27	0.60 (0.29 , 1.26)	0.240	0.163		
Intron	Phosphorus	Eukaryotes	0.03	25	0.14	1.31 (0.87 , 1.98)	0.433	0.199		
		Chlorophytes	0.96	10	1.06	1.60 (1.36 , 1.89)	<0.001	<0.001	1.230	0.267
		Diatoms	0.23	11	1.08	-1.13 (-2.10 , -0.61)	0.133	0.682		
	Nitrogen	Eukaryotes	0.27	25	-1.33	1.31 (0.92 , 1.88)	0.007	0.136		
		Chlorophytes	0.79	11	-1.17	1.58 (1.13 , 2.21)	<0.001	0.012	7.099	0.008
		Diatoms	0.01	9	1.27	-0.47 (-1.05 , -0.21)	0.766	0.066		
Av. Intergenic	Phosphorus	Cyanobacteria	0.87	8	2.42	0.26 (0.18 , 0.36)	0.001	<0.001		
		Eukaryotes	0.49	25	2.92	0.57 (0.42 , 0.78)	<0.001	0.001	9.678	0.002
		Chlorophytes	0.78	10	3.14	0.81 (0.56 , 1.19)	0.001	0.252	4.573	0.032
		Diatoms	0.60	11	2.75	0.43 (0.27 , 0.69)	0.005	0.002		

	Nitrogen	Eukaryotes	0.55	25	2.02	0.68 (0.51 , 0.9)	<0.001	0.009	9.430	0.002
		Chlorophytes	0.73	11	2.00	0.81 (0.56 , 1.19)	0.001	0.255		
		Diatoms	0.79	9	2.40	0.33 (0.22 , 0.5)	0.001	<0.001		
Av. Intron	Phosphorus	Eukaryotes	0.20	25	2.17	0.49 (0.34 , 0.71)	0.024	<0.001	0.008	0.928
		Chlorophytes	0.53	10	2.47	0.48 (0.28 , 0.81)	0.017	0.010		
		Diatoms	0.44	11	1.82	0.46 (0.27 , 0.79)	0.026	0.008		
	Nitrogen	Eukaryotes	0.16	25	1.50	0.54 (0.36 , 0.79)	0.051	0.002	7.080	0.008
		Chlorophytes	0.50	11	1.80	0.49 (0.29 , 0.82)	0.015	0.010		
		Diatoms	0.01	9	2.40	-0.13 (-0.30 , -0.06)	0.813	<0.001		

Table S5. Coefficients and summary statistics for the SMA regression analysis between the size of different DNA regions and corrected Q_{min} for phosphorus (P) and nitrogen (N), excluding the N and P cost of the corresponding DNA.

Regressions were fitted only when the number of observations (n) was greater than 4. The 95% confidence intervals for slopes are shown in brackets. P-slope$_0$ and p-slope$_1$ indicate p-values for testing whether slope coefficients are equal to 0 and 1, respectively. Likelihood ratio test (LR) and corresponding p-values (p_{LR}) are provided for comparing slopes between prokaryotes and eukaryotes, and between chlorophytes and diatoms.

DNA region	Nutrient	Taxon	R^2	n	Intercept	Slope	p-slope$_0$	p-slope$_1$	LR	p_{LR}
Genome	Phosphorus	Cyanobacteria	0.75	8	0.70	0.22 (0.13 , 0.35)	0.006	<0.001		
		Eukaryotes	0.51	38	1.69	0.64 (0.50 , 0.81)	<0.001	<0.001	10.714	0.001
		Chlorophytes	0.76	15	1.82	0.55 (0.41 , 0.73)	<0.001	<0.001	0.167	0.683
		Diatoms	0.32	15	1.35	0.62 (0.38 , 0.99)	0.027	0.045		
	Nitrogen	Eukaryotes	0.63	35	0.70	0.69 (0.56 , 0.86)	<0.001	0.001		
		Chlorophytes	0.78	15	0.93	0.57 (0.43 , 0.75)	<0.001	<0.001	0.002	0.966
		Diatoms	0.60	13	0.71	0.58 (0.38 , 0.87)	0.002	0.011		
Non-coding	Phosphorus	Cyanobacteria	0.87	8	0.01	0.41 (0.29 , 0.58)	0.001	<0.001		
		Eukaryotes	0.39	25	1.26	0.84 (0.60 , 1.17)	0.001	0.298	8.095	0.004
		Chlorophytes	0.90	10	1.61	1.21 (0.94 , 1.56)	<0.001	0.123	7.376	0.007
		Diatoms	0.40	11	1.03	0.51 (0.29 , 0.89)	0.036	0.020		
	Nitrogen	Eukaryotes	0.52	25	-0.14	1.05 (0.78 , 1.41)	<0.001	0.748		
		Chlorophytes	0.80	11	-0.13	1.18 (0.84 , 1.65)	<0.001	0.302	3.421	0.064
		Diatoms	0.19	9	0.41	0.56 (0.27 , 1.17)	0.242	0.114		
Coding	Phosphorus	Cyanobacteria	0.59	8	0.59	0.18 (0.10 , 0.33)	0.027	<0.001		
		Eukaryotes	0.22	25	1.28	0.27 (0.18 , 0.39)	0.019	<0.001	1.286	0.257
		Chlorophytes	0.73	10	1.31	0.23 (0.15 , 0.34)	0.002	<0.001	0.439	0.508
		Diatoms	0.03	11	1.61	-0.29 (-0.58 , -0.15)	0.641	0.001		
	Nitrogen	Eukaryotes	0.30	25	0.82	0.35 (0.24 , 0.49)	0.004	<0.001		
		Chlorophytes	0.79	11	1.00	0.19 (0.13 , 0.26)	<0.001	<0.001	2.161	0.142
		Diatoms	0.04	9	2.07	-0.35 (-0.77 , -0.16)	0.604	0.012		
Intergenic	Phosphorus	Cyanobacteria	0.87	8	0.00	0.41 (0.29 , 0.58)	0.001	<0.001		
		Eukaryotes	0.47	25	1.09	0.81 (0.59 , 1.10)	<0.001	0.166	7.693	0.006
		Chlorophytes	0.79	10	1.29	1.05 (0.73 , 1.51)	0.001	0.770	4.002	0.045
		Diatoms	0.36	11	0.94	0.54 (0.30 , 0.95)	0.051	0.035		
	Nitrogen	Eukaryotes	0.55	25	-0.25	1.01 (0.76 , 1.34)	<0.001	0.935		
		Chlorophytes	0.75	11	-0.20	1.04 (0.72 , 1.5)	0.001	0.820	1.918	0.166
		Diatoms	0.18	9	0.28	0.59 (0.28 , 1.25)	0.253	0.156		
Intron	Phosphorus	Eukaryotes	0.02	25	0.16	1.31 (0.86 , 1.98)	0.479	0.204		
		Chlorophytes	0.95	10	1.10	1.63 (1.37 , 1.95)	<0.001	<0.001	1.375	0.241
		Diatoms	0.23	11	1.08	-1.13 (-2.10 , -0.61)	0.133	0.689		
	Nitrogen	Eukaryotes	0.27	25	-1.32	1.31 (0.92 , 1.88)	0.008	0.135		
		Chlorophytes	0.79	11	-1.17	1.58 (1.13 , 2.22)	<0.001	0.013	7.131	0.008
		Diatoms	0.01	9	1.27	-0.47 (-1.05 , -0.21)	0.763	0.065		

Table S6. Coefficients and summary statistics for the SMA regression analysis between the size of different DNA regions and the cell volume.

Regressions were fitted only when the number of observations (n) was greater than 4. The 95% confidence intervals for slopes are shown in brackets. P-slope$_0$ and p-slope$_1$ indicate p-values for testing whether slope coefficients are equal to 0 and 1, respectively. Likelihood ratio test (LR) and corresponding p-values (p_{LR}) are provided for comparing slopes between prokaryotes and eukaryotes, and between chlorophytes and diatoms.

DNA region	Taxon	R^2	n	Intercept	Slope	p-slope$_0$	p-slope$_1$	LR	p_{LR}
Genome	Cyanobacteria	0.71	16	0.41	0.19 (0.14 , 0.26)	<0.001	<0.001		
	Eukaryotes	0.46	93	0.67	0.58 (0.50 , 0.68)	<0.001	<0.001	25.601	<0.001
	Chlorophytes	0.81	35	0.94	0.50 (0.43 , 0.58)	<0.001	<0.001	1.891	0.169
	Diatoms	0.40	38	0.83	0.41 (0.31 , 0.53)	<0.001	<0.001		
Non-coding	Cyanobacteria	0.67	16	-0.55	0.34 (0.25 , 0.47)	<0.001	<0.001		
	Eukaryotes	0.56	46	0.02	0.85 (0.69 , 1.04)	<0.001	0.106	17.788	<0.001
	Chlorophytes	0.78	22	0.27	0.70 (0.57 , 0.87)	<0.001	0.003	0.270	0.603
	Diatoms	0.25	14	0.21	0.61 (0.36 , 1.02)	0.069	0.061		
Coding	Cyanobacteria	0.60	16	0.35	0.17 (0.12 , 0.24)	<0.001	<0.001		
	Eukaryotes	0.54	46	0.86	0.28 (0.23 , 0.34)	<0.001	<0.001	5.917	0.015
	Chlorophytes	0.82	22	0.93	0.23 (0.19 , 0.28)	<0.001	<0.001	1.554	0.212
	Diatoms	0.02	14	2.08	-0.34 (-0.61 , -0.19)	0.633	0.001		
Intergenic	Cyanobacteria	0.68	16	-0.56	0.34 (0.25 , 0.47)	<0.001	<0.001		
	Eukaryotes	0.52	46	-0.10	0.82 (0.66 , 1.01)	<0.001	0.056	16.523	<0.001
	Chlorophytes	0.72	22	0.13	0.65 (0.51 , 0.83)	<0.001	0.001	0.003	0.960
	Diatoms	0.21	14	0.03	0.66 (0.39 , 1.12)	0.097	0.117		
Intron	Eukaryotes	0.32	46	-1.30	1.14 (0.89 , 1.46)	<0.001	0.303		
	Chlorophytes	0.78	22	-0.58	0.88 (0.71 , 1.09)	<0.001	0.227	1.727	0.189
	Diatoms	0.14	14	2.88	-1.30 (-2.26 , -0.75)	0.183	0.345		
Av. Intergenic	Cyanobacteria	0.47	16	2.02	0.22 (0.15 , 0.33)	0.003	<0.001		
	Eukaryotes	0.47	46	2.16	0.54 (0.44 , 0.68)	<0.001	<0.001	13.374	<0.001
	Chlorophytes	0.68	22	2.33	0.45 (0.35 , 0.59)	<0.001	<0.001	0.002	0.961
	Diatoms	0.13	14	2.14	0.46 (0.26 , 0.8)	0.212	0.008		
Av. Intron	Eukaryotes	0.23	46	1.66	0.39 (0.30 , 0.51)	0.001	<0.001		
	Chlorophytes	0.53	22	2.02	0.23 (0.17 , 0.32)	<0.001	<0.001	5.424	0.020
	Diatoms	0.09	14	1.18	0.51 (0.29 , 0.90)	0.296	0.021		

Table S7. Partition of the variation between Q_{min} (including both Q_{minP} and Q_{minN}) and cell volume for each DNA compartment in eukaryotes.

Q_{min}: Pure Q_{min} effect. Volume: Pure cell volume effect. Q_{min} & Vol.: shared variation between Q_{min} and cell volume. n: Number of observations.

DNA region	Q_{min}	Volume	Q_{min} & Vol.	n
Genome	0.08	0.01	0.62	30
Non-coding	0.04	0.04	0.58	21
Coding	0.06	0.02	0.38	21
Intergenic	0.03	0.05	0.63	21
Intron	0.03	0.03	0.29	21
Av. Intergenic	0.02	0.06	0.54	21
Av. Intron	0.07	0.01	0.17	21

Table S8. Pairwise comparisons of the lengths of intergenic and intronic regions between quartiles defined according to their median expression level per position. Comparisons were performed separately for intergenic and intronic regions of *Bathycoccus prasinos* and *Ostreococcus tauri*. Quartiles Q1 and Q4 group the regions with the lowest and highest expression levels, respectively. W: Wilcoxon statistic. n: number of observations per group.

Comparison between levels of expression	Intergenic regions			Introns			
	W	p-value	n	W	p-value	n	
Q1 vs Q2	2117625.5	<0.001	1963; 1963	72140	0.626	376; 376	*B. prasinos*
Q1 vs Q3	2023449.5	0.006	1963; 1963	88457.5	<0.001	376; 375	
Q1 vs Q4	1703790.5	<0.001	1963; 1962	104597.5	<0.001	376; 375	
Q2 vs Q3	1847582	0.026	1963; 1963	84678	<0.001	376; 375	
Q2 vs Q4	1544672	<0.001	1963; 1962	99880.5	<0.001	376; 375	
Q3 vs Q4	1636947	<0.001	1963; 1962	85207	<0.001	375; 375	
Q1 vs Q2	2129520	<0.001	1912; 1911	120281	0.001	524; 524	*O. tauri*
Q1 vs Q3	2506165	<0.001	1912; 1911	132012.5	0.282	524; 524	
Q1 vs Q4	2301037.5	<0.001	1912; 1911	174541.5	<0.001	524; 524	
Q2 vs Q3	2259931	<0.001	1911; 1911	146727	0.054	524; 524	
Q2 vs Q4	2030718	<0.001	1911; 1911	187242	<0.001	524; 524	
Q3 vs Q4	1601868.5	<0.001	1911; 1911	171826.5	<0.001	524; 524	

Table S9. Pairwise comparisons of minor allele frequency (MAF) between low and high nutrient levels, short (<10bp) and long (>=10bp) indels, and insertions and deletions. Comparisons between nutrient levels were performed from environmental phosphate concentration and from nitrate and nitrite concentration. Results pooling indels from all the species considered and separately for *Bathycoccus prasinos* and *Ostreococcus lucimarinus* are shown. W: Wilcoxon statistic. n: number of observations per group.

				Phosphate			Nitrate and nitrite			
Indel type	Indel length	Nutrient level	Comparison	W	p-value	n	W	p-value	n	
Insertion	Short	-	Low vs High	1794929	<0.001	2153; 1535	1812474.5	0.012	1794; 1929	Mamiellales
Insertion	Long	-	Low vs High	91.5	0.006	14; 28	160	0.131	21; 21	
Deletion	Short	-	Low vs High	2384072	0.001	2445; 1845	2320131.5	0.637	2122; 2205	
Deletion	Long	-	Low vs High	3278.5	0.546	73; 95	3374	0.644	88; 80	
Insertion	-	Low	Short vs Long	23069	<0.001	2153; 14	24242.5	0.024	1794; 21	
Insertion	-	High	Short vs Long	18467.5	0.202	1535; 28	18685	0.541	1929; 21	
Deletion	-	Low	Short vs Long	108633.5	0.002	2445; 73	109412.5	0.006	2122; 88	
Deletion	-	High	Short vs Long	97166.5	0.074	1845; 95	102273.5	0.015	2205; 80	
-	Short	Low	Ins. vs Del.	2470752.5	<0.001	2153; 2445	1753047	<0.001	1794; 2122	
-	Short	High	Ins. vs Del.	1369266	0.098	1535; 1845	2072868.5	0.159	1929; 2205	
-	Long	Low	Ins. vs Del.	649	0.112	14; 73	949	0.851	21; 88	
-	Long	High	Ins. vs Del.	966.5	0.029	28; 95	642	0.098	21; 80	
Insertion	Short	-	Low vs High	673283.5	0.032	1170; 1094	636972.5	0.124	1365; 899	*B. prasinos*
Insertion	Long	-	Low vs High	8.5	0.036	4; 15	23.5	0.086	10; 9	
Deletion	Short	-	Low vs High	773863.5	0.375	1330; 1188	779406	0.235	1521; 997	
Deletion	Long	-	Low vs High	511.5	0.325	29; 41	506.5	0.496	45; 25	
Insertion	-	Low	Short vs Long	4152.5	0.007	1170; 4	9557	0.029	1365; 10	
Insertion	-	High	Short vs Long	8081	0.920	1094; 15	3591.5	0.562	899; 9	
Deletion	-	Low	Short vs Long	25168.5	0.005	1330; 29	43030	0.003	1521; 45	
Deletion	-	High	Short vs Long	28235	0.082	1188; 41	14287	0.211	997; 25	
-	Short	Low	Ins. vs Del.	725617.5	0.004	1174; 1330	1003079.5	0.117	1365; 1521	
-	Short	High	Ins. vs Del.	655067	0.739	1094; 1188	437237	0.359	899; 997	
-	Long	Low	Ins. vs Del.	84	0.159	4; 29	252.5	0.556	10; 45	
-	Long	High	Ins. vs Del.	252.5	0.313	15; 41	80	0.211	9; 25	
Insertion	Short	-	Low vs High	89564	0.440	441; 394	75868.5	0.023	609; 226	*O. lucimarinus*
Insertion	Long	-	Low vs High	29.5	0.030	10; 13	23	0.109	18; 5	
Deletion	Short	-	Low vs High	182687	0.009	593; 566	153400	0.021	810; 349	
Deletion	Long	-	Low vs High	797	0.624	37; 46	501.5	0.081	61; 22	
Insertion	-	Low	Short vs Long	2954.5	0.066	441; 10	6319	0.269	609; 18	
Insertion	-	High	Short vs Long	1831.5	0.081	394; 13	266	0.043	226; 5	
Deletion	-	Low	Short vs Long	12369	0.193	593; 37	28128.5	0.071	810; 61	
Deletion	-	High	Short vs Long	12750	0.817	566; 46	3028.5	0.097	349; 22	
-	Short	Low	Ins. vs Del.	125055	0.230	441; 593	228570	0.018	609; 810	
-	Short	High	Ins. vs Del.	100296	0.008	394; 566	37224	0.255	226; 349	
-	Long	Low	Ins. vs Del.	224.5	0.311	10; 37	519.5	0.735	18; 61	
-	Long	High	Ins. vs Del.	183	0.035	13; 46	25	0.066	5; 22	

Data S1 (separate file).
Genomic traits.

Data S2 (separate file).
Minimum quotas for phosphorus and nitrogen.

Data S3 (separate file).
Cell volumes.

Data S4 (separate file).
List of mutant indel alleles from metagenomes.

REFERENCES

1. S. J. Giovannoni, H. J. Tripp, S. Givan, M. Podar, K. L. Vergin, D. Baptista, L. Bibbs, J. Eads, T. H. Richardson, M. Noordewier, M. S. Rappé, J. M. Short, J. C. Carrington, E. J. Mathur, Genome streamlining in a cosmopolitan oceanic bacterium. *Science* **309**, 1242–1245 (2005).

2. P. Fernández, R. Amice, D. Bruy, M. J. M. Christenhusz, I. J. Leitch, A. L. Leitch, L. Pokorny, O. Hidalgo, J. Pellicer, A 160 Gbp fork fern genome shatters size record for eukaryotes. *iScience* **27**, 109889 (2024).

3. D. A. Petrov, Evolution of genome size: New approaches to an old problem. *Trends Genet.* **17**, 23–28 (2001).

4. M. Lynch, J. S. Conery, The origins of genome complexity. *Science* **302**, 1401–1404 (2003).

5. T. R. Gregory, Coincidence, coevolution, or causation? DNA content, cell size, and the C-value enigma. *Biol. Rev.* **76**, 65–101 (2001).

6. J. Blommaert, Genome size evolution: Towards new model systems for old questions. *Proc. Biol. Sci.* **287**, 20201441 (2020).

7. M. Lynch, G. K. Marinov, The bioenergetic costs of a gene. *Proc. Natl. Acad. Sci. U.S.A.* **112**, 15690–15695 (2015).

8. M. R. Lynch, *Evolutionary Cell Biology: The Origins of Cellular Architecture* (Oxford Univ. Press, 2024).

9. B. K. Swan, B. Tupper, A. Sczyrba, F. M. Lauro, M. Martinez-Garcia, J. M. Gonźalez, H. Luo, J. J. Wright, Z. C. Landry, N. W. Hanson, B. P. Thompson, N. J. Poulton, P. Schwientek, S. G. Acinas, S. J. Giovannoni, M. A. Moran, S. J. Hallam, R. Cavicchioli, T. Woyke, R. Stepanauskas, Prevalent genome streamlining and latitudinal divergence of planktonic bacteria in the surface ocean. *Proc. Natl. Acad. Sci. U.S.A.* **110**, 11463–11468 (2013).

10. S. J. Giovannoni, J. Cameron Thrash, B. Temperton, Implications of streamlining theory for microbial ecology. *ISME J.* **8**, 1553–1565 (2014).

11. F. M. Lauro, D. Mcdougald, T. Thomas, T. J. Williams, S. Egan, S. Rice, M. Z. Demaere, L. Ting, H. Ertan, J. Johnson, S. Ferriera, A. Lapidus, I. Anderson, N. Kyrpides, A. C. Munk, C. Detter, C. S. Han, M. V. Brown, F. T. Robb, S. Kjelleberg, R. Cavicchioli, The genomic basis of trophic strategy in marine bacteria. *Proc. Natl. Acad. Sci. U.S.A.* **106**, 15527–15533 (2009).

12. J. G. Okie, A. T. Poret-Peterson, Z. M. P. Lee, A. Richter, L. D. Alcaraz, L. E. Eguiarte, J. L. Siefert, V. Souza, C. L. Dupont, J. J. Elser, Genomic adaptations in information processing underpin trophic strategy in a whole-ecosystem nutrient enrichment experiment. *eLife* **9**, e49816 (2020).

13. P. Šmarda, M. Hejcman, A. Březinová, L. Horová, H. Steigerová, F. Zedek, P. Bureš, P. Hejcmanová, J. Schellberg, Effect of phosphorus availability on the selection of species with different ploidy levels and genome sizes in a long-term grassland fertilization experiment. *New Phytol.* **200**, 911–921 (2013).

14. M. S. Guignard, R. A. Nichols, R. J. Knell, A. Macdonald, C. Romila, M. Trimmer, I. J. Leitch, A. R. Leitch, Genome size and ploidy influence angiosperm species' biomass under nitrogen and phosphorus limitation. *New Phytol.* **210**, 1195–1206 (2016).

15. L. Naselli-Flores, J. Padisák, Ecosystem services provided by marine and freshwater phytoplankton. *Hydrobiologia* **850**, 2691–2706 (2023).

16. A. Dufresne, L. Garczarek, F. Partensky, Accelerated evolution associated with genome reduction in a free-living prokaryote. *Genome Biol.* **6**, 1–10 (2005).

17. G. Rocap, F. W. Larimer, J. Lamerdin, S. Malfatti, P. Chain, N. A. Ahlgren, A. Arellano, M. Coleman, L. Hauser, W. R. Hess, Z. I. Johnson, M. Land, D. Lindell, A. F. Post, W. Regala, M. Shah, S. L. Shaw, C. Steglich, M. B. Sullivan, C. S. Ting, A. Tolonen, E. A. Webb, E. R. Zinser, S. W. Chisholm, Genome divergence in two *Prochlorococcus* ecotypes reflects oceanic niche differentiation. *Nature* **424**, 1042–1047 (2003).

18. E. Derelle, C. Ferraz, S. Rombauts, P. Rouzé, A. Z. Worden, S. Robbens, F. Dé Ric Partensky, S. Degroeve, S. Echeynié, R. Cooke, Y. Saeys, J. Wuyts, K. Jabbari, C. Bowler, O. Panaud,

B. Benoı̂t, P. Gu, S. G. Ball, J.-P. Ral, F.-Y. Bouget, G. Piganeau, B. De Baets, A. Picard, M. Delseny, J. Demaille, Y. Van De Peer, H. Moreau, Genome analysis of the smallest free-living eukaryote *Ostreococcus tauri* unveils many unique features. *Proc. Natl. Acad. Sci. U.S.A.* **103**, 11647–11652 (2006).

19. B. Palenik, J. Grimwood, A. Aerts, P. Rouzé, A. Salamov, N. Putnam, C. Dupont, R. Jorgensen, E. Derelle, S. Rombauts, K. Zhou, R. Otillar, S. S. Merchant, S. Podell, T. Gaasterland, C. Napoli, K. Gendler, A. Manuell, V. Tai, O. Vallon, G. Piganeau, S. Verine Jancek, M. Heijde, K. Jabbari, C. Bowler, M. Lohr, S. Robbens, G. Werner, I. Dubchak, G. J. Pazour, Q. Ren, I. Paulsen, C. Delwiche, J. Schmutz, D. Rokhsar, Y. Van De Peer, H. Moreau, I. V. Grigoriev, The tiny eukaryote *Ostreococcus* provides genomic insights into the paradox of plankton speciation. *Proc. Natl. Acad. Sci. U.S.A.* **104**, 7705–7710 (2007).

20. S. Yau, M. Krasovec, L. F. Benites, S. Rombauts, M. Groussin, E. Vancaester, J.-M. Aury, E. Derelle, Y. Desdevises, M.-L. Escande, N. Grimsley, J. Guy, H. Moreau, S. Sanchez-Brosseau, Y. Van de Peer, K. Vandepoele, S. Gourbiere, G. Piganeau, Virus-host coexistence in phytoplankton through the genomic lens. *Sci. Adv.* **6**, eaay2587 (2020).

21. T. J. Browning, C. M. Moore, Global analysis of ocean phytoplankton nutrient limitation reveals high prevalence of co-limitation. *Nat. Commun.* **14**, 5014 (2023).

22. A. C. Martiny, M. L. Coleman, S. W. Chisholm, Phosphate acquisition genes in *Prochlorococcus* ecotypes: Evidence for genome-wide adaptation. *Proc. Natl. Acad. Sci. U.S.A.* **103**, 12552–12557 (2006).

23. S. Lin, R. W. Litaker, W. G. Sunda, Phosphorus physiological ecology and molecular mechanisms in marine phytoplankton. *J. Phycol.* **52**, 10–36 (2016).

24. L. J. Ustick, A. A. Larkin, C. A. Garcia, N. S. Garcia, M. L. Brock, J. A. Lee, N. A. Wiseman, J. K. Moore, A. C. Martiny, Metagenomic analysis reveals global-scale patterns of ocean nutrient limitation. *Science* **372**, 287–291 (2021).

25. M. R. Droop, Vitamin B12 and marine ecology. IV. The kinetics of uptake, growth and inhibition in *Monochrysis lutheri*. *J. Mar. Biol. Assoc. U. K.* **48**, 689–733 (1968).

26. K. F. Edwards, M. K. Thomas, C. A. Klausmeier, E. Litchman, Allometric scaling and taxonomic variation in nutrient utilization traits and maximum growth rate of phytoplankton. *Limnol. Oceanogr.* **57**, 554–566 (2012).

27. S. J. Sibbald, J. M. Archibald, Genomic insights into plastid evolution. *Genome Biol. Evol.* **12**, 978–990 (2020).

28. T. R. Gregory, The C-value enigma in plants and animals: A review of parallels and an appeal for partnership. *Ann. Bot.* **95**, 133–146 (2005).

29. T. P. Michael, Plant genome size variation: Bloating and purging DNA. *Brief. Funct. Genomic. Proteomic.* **13**, 308–317 (2014).

30. J. Pellicer, O. Hidalgo, S. Dodsworth, I. J. Leitch, Genome size diversity and its impact on the evolution of land plants. *Genes.* **9**, 88 (2018).

31. V. Ebenezer, Y. Hu, O. Carnicer, A. J. Irwin, M. J. Follows, Z. V. Finkel, Elemental and macromolecular composition of the marine Chloropicophyceae, a major group of oceanic photosynthetic picoeukaryotes. *Limnol. Oceanogr.* **67**, 540–551 (2022).

32. J. D. Liefer, A. Garg, M. H. Fyfe, A. J. Irwin, I. Benner, C. M. Brown, M. J. Follows, A. W. Omta, Z. V. Finkel, The macromolecular basis of phytoplankton C:N:P under nitrogen starvation. *Front. Microbiol.* **10**, 763 (2019).

33. E. Marañón, P. Cermeño, D. C. López-Sandoval, T. Rodríguez-Ramos, C. Sobrino, M. Huete-Ortega, J. M. Blanco, J. Rodríguez, Unimodal size scaling of phytoplankton growth and the size dependence of nutrient uptake and use. *Ecol. Lett.* **16**, 371–379 (2013).

34. L. M. Chevin, On measuring selection in experimental evolution. *Biol. Lett.* **7**, 210–213 (2011).

35. L. M. Almassalha, M. Carignano, E. Pujadas Liwag, W. Shun Li, R. Gong, N. Acosta, C. L. Dunton, P. Carrillo Gonzalez, L. M. Carter, R. Kakkaramadam, M. Kröger, K. L. MacQuarrie, J. Frederick, I. Chae Ye, P. Su, T. Kuo, K. I. Medina, J. A. Pritchard, A. Skol, R. Nap, M. Kanemaki, V. Dravid, I. Szleifer, V. Backman, Chromatin conformation, gene

transcription, and nucleosome remodeling as an emergent system. *Sci. Adv.* **11**, eadq6652 (2025).

36. M. Kimura, *The Neutral Theory of Molecular Evolution* (Cambridge Univ. Press, 1983).

37. T. Y. Ho, A. Quigg, Z. V. Finkel, A. J. Milligan, K. Wyman, P. G. Falkowski, F. M. M. Morel, The elemental composition of some marine phytoplankton. *J. Phycol.* **39**, 1145–1159 (2003).

38. T. Cavalier-Smith, Economy, speed and size matter: Evolutionary forces driving nuclear genome miniaturization and expansion. *Ann. Bot.* **95**, 147–175 (2005).

39. J. A. Connolly, M. J. Oliver, J. M. Beaulieu, C. A. Knight, L. Tomanek, M. A. Moline, Correlated evolution of genome size and cell volume in diatoms (Bacillariophyceae). *J. Phycol.* **44**, 124–131 (2008).

40. H. Wang, P. Wu, L. Xiong, H. S. Kim, J. H. Kim, J. S. Ki, Nuclear genome of dinoflagellates: Size variation and insights into evolutionary mechanisms. *Eur. J. Protistol.* **93**, 126061 (2024).

41. W. R. Roberts, A. M. Siepielski, A. J. Alverson, Diatom abundance in the polar oceans is predicted by genome size. *PLOS Biol.* **22**, e3002733 (2024).

42. D. Čertnerová, P. Škaloud, I. Jadrná, M. Čertner, Large genomes are associated with greater cell size and ecological shift towards more nitrogen-rich and higher-latitude environments in microalgae of the genus *Synura*. *J. Euk. Microbiol.* **72**, e70026 (2025).

43. E. Litchman, C. A. Klausmeier, O. M. Schofield, P. G. Falkowski, The role of functional traits and trade-offs in structuring phytoplankton communities: Scaling from cellular to ecosystem level. *Ecol. Lett.* **10**, 1170–1181 (2007).

44. C. I. Castillo-Davis, S. L. Mekhedov, D. L. Hartl, E. V. Koonin, F. A. Kondrashov, Selection for short introns in highly expressed genes. *Nat. Genet.* **31**, 415–418 (2002).

45. L. Shenhav, D. Zeevi, Resource conservation manifests in the genetic code. *Science* **370**, 683–687 (2020).

46. S. A. Sawyer, D. L. Hartlt, Population genetics of polymorphism and divergence. *Genetics* **132**, 1161–1176 (1992).

47. G. Sommeria-Klein, R. Watteaux, F. M. Ibarbalz, J. J. P. Karlusich, D. Iudicone, C. Bowler, H. Morlon, Global drivers of eukaryotic plankton biogeography in the sunlit ocean. *Science* **374**, 594–599 (2021).

48. S. Sunagawa, S. G. Acinas, P. Bork, C. Bowler, M. Babin, E. Boss, G. Cochrane, C. de Vargas, M. Follows, G. Gorsky, N. Grimsley, L. Guidi, P. Hingamp, D. Iudicone, O. Jaillon, S. Kandels, L. Karp-Boss, E. Karsenti, M. Lescot, F. Not, H. Ogata, S. Pesant, N. Poulton, J. Raes, C. Sardet, M. Sieracki, S. Speich, L. Stemmann, M. B. Sullivan, P. Wincker, D. Eveillard, F. Lombard, Tara Oceans: Towards global ocean ecosystems biology. *Nat. Rev. Microbiol.* **18**, 428–445 (2020).

49. J. Leconte, L. F. Benites, T. Vannier, P. Wincker, G. Piganeau, O. Jaillon, Genome resolved biogeography of Mamiellales. *Genes.* **11**, 66 (2020).

50. F. Partensky, L. Garczarek, *Prochlorococcus*: Advantages and limits of minimalism. *Ann. Rev. Mar. Sci.* **2**, 305–331 (2010).

51. A. Longhurst, *Ecological Geography of the Sea* (Academic Press, 2007).

52. D. Karl, A. Michaels, B. Bergman, D. Capone, E. Carpenter, R. Letelier, F. Lipschultz, H. Paerl, D. Sigman, L. Stal, Dinitrogen fixation in the world's oceans. *Biogeochemistry* **57-58**, 47–98 (2002).

53. R. E. Tuerena, C. Mahaffey, S. F. Henley, C. de la Vega, L. Norman, T. Brand, T. Sanders, M. Debyser, K. Dähnke, J. Braun, C. März, Nutrient pathways and their susceptibility to past and future change in the Eurasian Arctic Ocean. *Ambio* **51**, 355–369 (2022).

54. D. M. Karl, Microbially mediated transformations of phosphorus in the sea: New views of an old cycle. *Ann. Rev. Mar. Sci.* **6**, 279–337 (2014).

55. S. Duhamel, J. M. Diaz, J. C. Adams, K. Djaoudi, V. Steck, E. M. Waggoner, Phosphorus as an integral component of global marine biogeochemistry. *Nat. Geosci.* **14**, 359–368 (2021).

56. S. D. Gerace, J. Yu, J. K. Moore, A. C. Martiny, Observed declines in upper ocean phosphate-to-nitrate availability. *Proc. Natl. Acad. Sci. U.S.A.* **122**, e2411835122 (2025).

57. R. W. Sterner, J. J. Elser, *Ecological Stoichiometry: The Biology of Elements from Molecules to the Biosphere* (Princeton Univ. Press, 2003).

58. K. R. Arrigo, Marine microorganisms and global nutrient cycles. *Nature* **437**, 349–355 (2005).

59. R. M. Greene, R. J. Geider, P. G. Falkowski, Effect of iron limitation on photosynthesis in a marine diatom. *Limnol. Oceanogr.* **36**, 1772–1782 (1991).

60. J. S. P. McCain, A. Tagliabue, E. Susko, E. P. Achterberg, A. E. Allen, E. M. Bertrand, Cellular costs underpin micronutrient limitation in phytoplankton. *Sci. Adv.* **7**, eabg6501 (2021).

61. N. J. Hawco, A. Tagliabue, B. S. Twining, Manganese limitation of phytoplankton physiology and productivity in the Southern Ocean. *Global Biogeochem. Cycles* **36**, e2022GB007382 (2022).

62. A. Tagliabue, A. R. Bowie, P. W. Boyd, K. N. Buck, K. S. Johnson, M. A. Saito, The integral role of iron in ocean biogeochemistry. *Nature* **543**, 51–59 (2017).

63. M. R. Droop, Some thoughts on nutrient limitation in algae. *J. Phycol.* **9**, 264–272 (1973).

64. C. L. Woodcock, A. I. Skoultchi, Y. Fan, Role of linker histone in chromatin structure and function: H1 stoichiometry and nucleosome repeat length. *Chromosome Res.* **14**, 17–25 (2006).

65. M. Herzog, M. O. Soyer, Distinctive features of dinoflagellate chromatin. Absence of nucleosomes in a primitive species *Prorocentrum micans*. *Eur. J. Cell Biol.* **23**, 295–302 (1981).

66. S. Roy, D. Morse, A full suite of histone and histone modifying genes are transcribed in the dinoflagellate *Lingulodinium*. *PLOS ONE* **7**, e34340 (2012).

67. E. Litchman, C. A. Klausmeier, Trait-based community ecology of phytoplankton. *Annu. Rev. Ecol. Evol. Syst.* **39**, 615–639 (2008).

68. S. Kumar, S. Kaur, K. Seem, S. Kumar, T. Mohapatra, Understanding 3D genome organization and its effect on transcriptional gene regulation under environmental stress in plant: A chromatin perspective. *Front. Cell Dev. Biol.* **9**, 774719 (2021).

69. E. Harrison, Y. Meeda, T. Gaikwad, G. Wheeler, K. Helliwell, Nitrogen status exerts dynamic control over phosphorus sensing and acquisition via PSR1 in colimited marine diatoms. *Sci. Adv.* **11**, eadw8260 (2025).

70. M. Kimura, On the probability of fixation of mutant genes in a population. *Genetics* **47**, 713–719 (1962).

71. S. Wright, Evolution in Mendelian populations. *Genetics* **16**, 97–159 (1931).

72. R. I. Figueroa, M. Estrada, E. Garcés, Life histories of microalgal species causing harmful blooms: Haploids, diploids and the relevance of benthic stages. *Harmful Algae* **73**, 44–57 (2018).

73. J. R. Rawson, The characterization of *Euglena gracilis* DNA by its reassociation kinetics. *Biochim. Biophys. Acta* **402**, 171–178 (1975).

74. S. Marguerat, A. Schmidt, S. Codlin, W. Chen, R. Aebersold, J. Bähler, Quantitative analysis of fission yeast transcriptomes and proteomes in proliferating and quiescent cells. *Cell* **151**, 671–683 (2012).

75. R Core Team, R: A language and environment for statistical computing (2021).

76. E. Paradis, K. Schliep, ape 5.0: An environment for modern phylogenetics and evolutionary analyses in R. *Bioinformatics* **35**, 526–528 (2019).

77. D. Charif, J. R. Lobry, “SeqinR 1.0-2: A contributed package to the R project for statistical computing devoted to biological sequences retrieval and analysis,” in *Structural Approaches to Sequence Evolution: Molecules*, *Networks*, *Populations* (Springer, 2007), pp. 207–232.

78. M. Lawrence, W. Huber, H. Pagès, P. Aboyoun, M. Carlson, R. Gentleman, M. T. Morgan, V. J. Carey, Software for computing and annotating genomic ranges. *PLOS Comput. Biol.* **9**, e1003118 (2013).

79. C. T. Ahlmann-Eltze, tidygenomics: Tidy verbs for dealing with genomic data frames, R package version 0.1.2 (2019).

80. Z. V. Finkel, M. J. Follows, A. J. Irwin, Size-scaling of macromolecules and chemical energy content in the eukaryotic microalgae. *J. Plankton Res.* **38**, 1151–1162 (2016).

81. R. Marcellin-Gros, G. Piganeau, D. Stien, Metabolomic insights into marine phytoplankton diversity. *Mar. Drugs* **18**, 78 (2020).

82. M. Pujo-Pay, P. Raimbault, improvement of the wet-oxidation procedure for simultaneous determination of particulate organic nitrogen and phosphorus collected on filters. *Mar. Ecol. Prog. Ser.* **105**, 203–207 (1994).

83. M. D. Guiry, G. M. Guiry, AlgaeBase, University of Galway (2025). www.algaebase.org.

84. P. Legendre, L. Legendre, *Numerical Ecology* (Elsevier, ed. 2, 1998).

85. G. P. Quinn, M. J. Keough, *Experimental Design and Data Analysis for Biologists* (Cambridge Univ. Press, 2012).

86. H. Wickham, R. François, L. Henry, K. Müller, D. Vaughan, dplyr: A grammar of data manipulation, R package version 1.1.4 (2023).

87. H. Wickham, D. Vaughan, M. Girlich, tidyr: tidy messy data, R package version 1.3.1 (2024).

88. H. Wickham, stringr: Simple, consistent wrappers for common string operations, R package version 1.5.1 (2023).

89. H. Wickham, *ggplot2: Elegant Graphics for Data Analysis* (Springer, 2016).

90. T. L. Pedersen, ggforce: Accelerating ggplot2, R package version 0.5.0 (2025).

91. T. van den Brand, ggh4x: Hacks for ggplot2, R package version 0.2.8 (2024).

92. T. L. Pedersen, patchwork: the composer of plots, R package version 1.2.0 (2024).

93. R. S. Bivand, E. J. Pebesma, V. Gomez-Rubio, *Applied Spatial Data Analysis with R* (Springer, 2013).

94. D. I. Warton, R. A. Duursma, D. S. Falster, S. Taskinen, smatr 3-an R package for estimation and inference about allometric lines. *Methods Ecol. Evol.* **3**, 257–259 (2012).

95. M. Pujo-Pay, P. Conan, P. Raimbault, Excretion of dissolved organic nitrogen by phytoplankton assessed by wet oxidation and 15N tracer procedures. *Mar. Ecol. Prog. Ser.* **153**, 99–111 (1997).

96. Md. Vasimuddin, S. Misra, H. Li, S. Aluru, "Efficient architecture-aware acceleration of BWA-MEM for multicore systems," in *2019 IEEE International Parallel and Distributed Processing Symposium* (*IPDPS*) (IEEE, 2019), pp. 314–324.

97. H. Li, B. Handsaker, A. Wysoker, T. Fennell, J. Ruan, N. Homer, G. Marth, G. Abecasis, R. Durbin, The sequence alignment/map format and SAMtools. *Bioinformatics* **25**, 2078–2079 (2009).

98. M. Krasovec, R. Merret, F. Sanchez, S. Sanchez-Brosseau, G. Piganeau, A high frequency of chromosomal duplications in unicellular algae is compensated by translational regulation. *Genome Biol. Evol.* **15**, evad086 (2023).

99. E. Derelle, S. Yau, H. Moreau, N. H. Grimsley, Prasinovirus attack of *Ostreococcus* is furtive by day but savage by night. *J. Virol.* **92**, e01703–e01717 (2018).

100. L. Guidi, P. Morin, L. Coppola, J.-É. Tremblay, S. Pesant, Tara Oceans Consortium Coordinators, Tara Oceans Expedition Participants, Environmental context of all samples from the Tara Oceans Expedition (2009-2013), about nutrients in the targeted environmental feature (PANGAEA, 2017); https://doi.org/10.1594/PANGAEA.875575.

101. S. Pesant, F. Not, M. Picheral, S. Kandels-Lewis, N. Le Bescot, G. Gorsky, D. Iudicone, E. Karsenti, S. Speich, R. Troublé, C. Dimier, S. Searson, Tara Oceans Consortium Coordinators, Open science resources for the discovery and analysis of Tara Oceans data. *Sci. Data* **2**, 150023 (2015).

102. D. Kim, J. M. Paggi, C. Park, C. Bennett, S. L. Salzberg, Graph-based genome alignment and genotyping with HISAT2 and HISAT-genotype. *Nat. Biotechnol.* **37**, 907–915 (2019).

103. A. McKenna, M. Hanna, E. Banks, A. Sivachenko, K. Cibulskis, A. Kernytsky, K. Garimella, D. Altshuler, S. Gabriel, M. Daly, M. A. DePristo, The Genome Analysis Toolkit: A MapReduce framework for analyzing next-generation DNA sequencing data. *Genome Res.* **20**, 1297–1303 (2010).

104. P. Cingolani, A. Platts, L. L. Wang, M. Coon, T. Nguyen, L. Wang, S. J. Land, X. Lu, D. M. Ruden, A program for annotating and predicting the effects of single nucleotide polymorphisms, SnpEff. *Fly (Austin)* **6**, 80–92 (2012).

105. Z. Chen, X. Wang, Y. Song, Q. Zeng, Y. Zhang, H. Luo, *Prochlorococcus* have low global mutation rate and small effective population size. *Nat. Ecol. Evol.* **6**, 183–194 (2022).

106. R. Blanc-Mathieu, M. Krasovec, M. Hebrard, S. Yau, E. Desgranges, J. Martin, W. Schackwitz, A. Kuo, G. Salin, C. Donnadieu, Y. Desdevises, S. Sanchez-Ferandin, H. Moreau, E. Rivals, I. V. Grigoriev, N. Grimsley, A. Eyre-Walker, G. Piganeau, Population genomics of picophytoplankton unveils novel chromosome hypervariability. *Sci. Adv.* **3**, e1700239 (2017).

107. M. Krasovec, S. Sanchez-Brosseau, G. Piganeau, First estimation of the spontaneous mutation rate in diatoms. *Genome Biol. Evol.* **11**, 1829–1837 (2019).

108. R. W. Ness, S. A. Kraemer, N. Colegrave, P. D. Keightley, Direct estimate of the spontaneous mutation rate uncovers the effects of drift and recombination in the *Chlamydomonas reinhardtii* plastid genome. *Mol. Biol. Evol.* **33**, 800–808 (2016).

109. M. Krasovec, R. E. M. Rickaby, D. A. Filatov, Evolution of mutation rate in astronomically large phytoplankton populations. *Genome Biol. Evol.* **12**, 1051–1059 (2020).
